# Integrated Analysis of Genome-Wide Copy Number Alterations and Gene Expression Profiling of Lung Cancer in Xuanwei, China

**DOI:** 10.1371/journal.pone.0169098

**Published:** 2017-01-05

**Authors:** Yanliang Zhang, Qiuyue Xue, Guoqing Pan, Qing H. Meng, Xiaoyu Tuo, Xuemei Cai, Zhenghui Chen, Ya Li, Tao Huang, Xincen Duan, Yong Duan

**Affiliations:** 1 Department of Clinical Laboratory, First Affiliated Hospital of Kunming Medical University, Kunming, Yunnan Province, the People's Republic of China; 2 Yunnan Institute of Laboratory Diagnosis, Kunming, Yunnan Province, the People's Republic of China; 3 Yunnan Key Laboratory of Laboratory Medicine, Kunming, Yunnan Province, the People's Republic of China; 4 Department of Pathology, First Affiliated Hospital of Kunming Medical University, Kunming, Yunnan Province, the People's Republic of China; 5 Department of Laboratory Medicine, University of Texas MD Anderson Cancer Center, Houston, Texas, United States of America; 6 Department of Thoracic Surgery, First Affiliated Hospital of Kunming Medical University, Kunming, Yunnan Province, the People's Republic of China; 7 Department of Biological Sciences, University of Wisconsin-Parkside, Somers, Wisconsin, United States of America; Universitatsmedizin Greifswald, GERMANY

## Abstract

**Objectives:**

Lung cancer in Xuanwei (LCXW), China, is known throughout the world for its distinctive characteristics, but little is known about its pathogenesis. The purpose of this study was to screen potential novel “driver genes” in LCXW.

**Methods:**

Genome-wide DNA copy number alterations (CNAs) were detected by array-based comparative genomic hybridization and differentially expressed genes (DEGs) by gene expression microarrays in 8 paired LCXW and non-cancerous lung tissues. Candidate driver genes were screened by integrated analysis of CNAs and DEGs. The candidate genes were further validated by real-time quantitative polymerase chain reaction.

**Results:**

Large numbers of CNAs and DEGs were detected, respectively. Some of the most frequently occurring CNAs included gains at 5p15.33-p15.32, 5p15.1-p14.3, and 5p14.3-p14.2 and losses at 11q24.3, 21q21.1, 21q22.12-q22.13, and 21q22.2. Integrated analysis of CNAs and DEGs identified 24 candidate genes with frequent copy number gains and concordant upregulation, which were considered potential oncogenes, including *CREB3L4*, *TRIP13*, and *CCNE2*. In addition, the analysis identified 19 candidate genes with a negative association between copy number change and expression change, considered potential tumor suppressor genes, including *AHRR*, *NKD2*, and *KLF10*. One of the most studied oncogenes, *MYC*, may not play a carcinogenic role in LCXW.

**Conclusions:**

This integrated analysis of CNAs and DEGs identified several potential novel LCXW-related genes, laying an important foundation for further research on the pathogenesis of LCXW and identification of novel biomarkers or therapeutic targets.

## Introduction

Lung cancer is the leading cause of cancer mortality worldwide. It is the fastest-increasing cancer in China and has been the leading cause of cancer death in China since 2004 [1]. The incidence of lung cancer is particularly high in some regions of the country, such as Xuanwei and Gejiu. Despite recent advances in surgical and chemo/radiation therapies, the prognosis of lung cancer is still very poor, with a 5-year overall survival rate of only ~15%. Thus, the need to combat lung cancer in China is unprecedented and still growing.

Xuanwei City (formerly known as Xuanwei County) is located in the northeast of Yunnan Province, China. It is 102 km from east to west, and 91 km from north to south, with a total area of 6,257 km^2^. The morbidity and mortality rates of lung cancer in Xuanwei are the highest in China and have shown clear upward trends since the mid-1970s [2]. Coal is the major resource in the Xuanwei area. Xuanwei residents traditionally use one or more of three different types of fuel—smoky coal (bituminous coal), smokeless coal (anthracite coal), and wood—in unvented indoor firepits for cooking and heating [3]. When burning smoky coal, the indoor air concentrations of particulate matter and extractable organic matter may reach as high as 24.4 mg/m^3^ and 17.6 mg/m^3^, respectively, and the corresponding benzo[a]pyrene concentration, an indicator of carcinogenic polycyclic aromatic hydrocarbons (PAHs), can reach as high as 19.3 μg/m^3^ which is comparable to exposure levels experienced by coke oven workers [4].

Epidemiological studies have suggested that the high incidence of LCXW is due mainly to the burning of smoky coal indoors without adequate ventilation [3,5–7]. The lung cancers that develop in Xuanwei show distinct characteristics [1] and are referred to as LCXW. In some villages, the mortality rate of female patients is as high as 400 per 100,000. In fact, women in Xuanwei, who are mostly nonsmokers (smoking rate < 1%), have the highest lung cancer rate in China. The sex ratio of lung cancer mortality rates between males and females in Xuanwei is 1.09, which is significantly lower than the national average of 2.09. LCXW incidence peaks at a younger age (41–50 years), more than 10 years younger than the peak incidence of lung cancer in other areas of China. Finally, LCXW mortality is strongly correlated with domestic use of smoky coal.

Most cancers are characterized by differentially expressed genes (DEGs), genes whose expression is significantly different in cancerous cells than in their nearby normal cells. These genes are assumed to play important roles in the occurrence and development of cancers. Gene expression profiling by microarray analysis has been shown to be a powerful tool for identification of cancer-related genes. This analysis, however, usually detects a large number of DEGs, and therefore the key challenge in expression profiling analysis is how to pinpoint which DEGs are critical to cancer formation (“driver genes”) and which are not (“passenger genes”).

Cancer is a genetic disease of altered somatic cells arising from accumulation of genetic changes. DNA copy number alteration (CNA), an important type of genetic alterations in various cancers, can contribute to the development and progression of cancer by altering the expression of genes within the regions of copy number changes [8]. Recent studies have indicated that integrated analysis of DNA CNAs and corresponding DEGs is an effective approach to identify the driver genes in multiple cancer types [9,10].

Previous studies of LCXW are focused mainly on its epidemiology, and little is known about its pathogenesis. Because of its distinctive etiology and characteristics, the pathogenesis of LCXW may be different from that of lung cancers occurring in other geographic areas. LCXW provides us with a unique opportunity to research the pathogenesis of non-tobacco-related lung cancer. Our purpose here was to screen for potential novel driver genes in LCXW through integrated analysis of genome-wide DNA CNAs and DEGs from paired LCXW and non-cancerous lung (NCL) tissues.

## Methods

### Sample Collection

Primary lung adenocarcinoma and paired NCL tissues (> 5 cm from carcinoma tissues) were collected from 84 patients from Xuanwei at the First Affiliated Hospital of Kunming Medical University, Kunming, China. The samples were fresh frozen and stored. The 8 paired samples collected at first were tested by microarrays and the rest samples were used for validation analysis. Written informed consent was obtained from all patients. The study was approved by the Institutional Review Board for the Use of Human Subjects at Kunming Medical University. All samples were assessed by an experienced pathologist to confirm the presence (> 80%) or absence of cancer cells. Clinicopathological characteristics of all patients were collected (Table 1). None of the patients received chemotherapy or radiotherapy treatment prior to surgery.

**Table 1:** Baseline clinicopathologic features of a cohort of lung cancer patients in Xuanwei, China.

Characteristic		No. of patients (%) N = 84
Sex	Male	50 (59%)
	Female	34 (41%)
Age, years	< 55	60 (71%)
	≥ 55	24 (29%)
Smoking, ever	Yes	34 (41%)
	No	50 (59%)
FIGO staging	I + II	64 (76%)
	III	20 (24%)
Lymphatic metastasis	Yes	36 (43%)
	No	48 (57%)

### Isolation of Nucleic Acids

Genomic DNA was extracted by using the DNeasy Blood & Tissue Kit (Qiagen, Hilden, Germany), and RNA was isolated by using the PureLink^®^ RNA Mini Kit (Thermo Fisher Scientific, Waltham, MA, USA), both according to the manufacturers^’^ protocols.

### Array-Based Comparative Genomic Hybridization Analysis

Oligonucleotide array-based comparative genomic hybridization (array-CGH) analysis was carried out on the 8 paired samples using Roche NimbleGen Human CGH 3×720K WG-T v3.0 Array (NimbleGen, Madison, WI, USA) according to the manufacturer’s protocol. All array-CGH coordinates in this study were mapped against the human genome as defined by the UCSC build hg18. The log2 copy-number ratio calculation and CNA calls were determined by using the segMNT algorithm in NimbleScan. Log2 ratio test/control thresholds of 0.25 and –0.25 were defined as copy number gains and losses, respectively. Deviant signal intensity ratios involving 5 or more neighboring probes were considered genomic aberrations.

### Gene Expression Microarray

Gene expression profiling analysis was performed on the same 8 paired samples using the Agilent Oligo Microarray Kit 8×60K according to the Agilent One-Color Microarray-based Gene Expression Analysis Protocol (Agilent Technologies, Santa Clara, CA, USA). The data were analyzed by GeneSpring software GX 12.6 (Agilent Technologies). Significantly DEGs were identified by using the mixed model analysis of variance [11] with a false discovery rate (Benjamini–Hochberg test) adjusted *p* value of ≤ 0.05 and absolute fold-change values ≥ 2 or ≤ 0.5. Hierarchical clustering was generated to visualize patterns of expression using cluster 3.0. Gene ontology (GO) analysis and Pathway analysis were performed using MAS 3.0. Pathway enrichment analysis was performed by using the latest KEGG database (http://www.kegg.jp/).

### Integrated Analysis

Integrated analysis for array-CGH data and gene expression data consisted of 4 steps as follows. In step 1, recurrent CNAs across samples were identified. Recurrent CNAs were defined as genomic segments that were altered in at least 3 samples. In step 2, concordant recurrent CNAs were identified. Three kinds of recurrent CNAs from step 1 were filtered out: the CNAs whose changes were inconsistent among samples, the CNAs that did not include any gene, and the copy number gains that include only partial segments of a gene. In step3, DEGs in CNAs were identified. The DEGs presented in the concordant recurrent CNA regions from step 2 were selected, while unchanged genes were filtered out. In step 4, candidate driver genes were pinpointed by searching the PubMed database (http://www.ncbi.nlm.nih.gov/pubmed) to retrieve current knowledge about DEGs identified from step 3, their function and role in cancer; genes that had a potential role in tumorigenesis and had not previously been reported in lung cancer were screened out for further study.

### Real-Time Quantitative Polymerase Chain Reaction (RT-qPCR) Analysis

Firstly, the candidate genes selected by the integrated analysis were validated in 8 paired samples by real-time quantitative polymerase chain reaction (RT-qPCR). Then, RT-qPCR also was used to determine copy number changes in these genes in the other 76 paired samples and gene expression changes in 50 of the paired samples. Gene expression analysis was not possible for 26 of the paired samples because of sample degradation. *GAPDH* was selected as an internal control. The primer sets were designed using the Primer Premier 5.0 (Primer, Canada) (Table 2). RT-qPCR was performed using SYBR^®^Premix Ex TaqTM SYBR Green I (TaKaRa, Dalian, China) on the ABI 7300 Sequence Detection System (Applied Biosystems, Foster City, CA, USA) and replicated three times. Cycling conditions were 95°C for 15 s followed by 40 cycles of 95°C (5 s), 60°C (15 s) and one cycle of 95°C (15 s), 60°C (60 s), 95°C (15 s). The data were analyzed by the 2^-ΔΔCt^ method. 2^-ΔΔCt^ ≥ 1.5 or ≤ 0.5 was defined as copy number gain or loss, respectively, and 2^-ΔΔCt^ ≥ 2 or ≤ 0.5 was defined as upregulation or downregulation, respectively.

**Table 2:** Primers used for detecting both copy number changes and expression changes in 7 candidate genes.

Analysis	Gene	Primers	Length of products (bp)
Copy number detection	*CREB3L4*	F: 5′-TTCCGTTTGTGGACCCTCAG-3′	296
		R: 5′-CCTCACCTGTCCCCTCGATA-3′	
	*TRIP13*	F: 5′-CCCCAGCACTTCGGTTCA-3′	116
		R: 5′-GCCCTTTCTCCCGCCTTT-3′	
	*CCNE2*	F: 5′-CATGGTCGGATTAACTCACACG-3′	336
		R: 5′-CCTGCATTCTGTCCCACCTTA-3′	
	*AHRR*	F: 5′-CAGACAGGCAGGAATGAACA-3′	116
		R: 5′-TAGGAAGGAAGGGAAGG-3′	
	*KLF10*	F: 5′-TTGTCATCCAAATGACACACAGA-3′	256
		R: 5′-GTGCCTCTCTCCCATGAACG-3′	
	*MYC*	F: 5′-AGAGTTTCATCTGCGACCCG-3′	259
		R: 5′-AGAGGGTAGGGGAAGACCAC-3′	
	*NKD2*	F: 5′-CCTAAACTGGGCATCTGTGG-3′	119
		R: 5′-CTCTCTGGCTCCTGCTGACT-3′	
	*GAPDH*	F: 5′-CCACCACACTGAATCTCCCC-3′	262
		R: 5′-CGAAGCAAGCAAGGCTGTTT-3′	
Gene expression detection	*CREB3L4*	F: 5′-CTGCCCTGTCAAACCCTGTT-3′	142
		R: 5′-GCTTGTTACGGATTTTCCTCCT-3′	
	*TRIP13*	F: 5′-CTGGAGGAAGAGACAGAAAACATAA-3′	134
		R: 5′-GTTGTCATCACATAATCGAGGAGAT-3′	
	*CCNE2*	F: 5′-GGAACCACAGATGAGGTCCAT-3′	237
		R: 5′-CCATCAGTGACGTAAGCAAACT-3′	
	*AHRR*	F: 5′-GCGGACGGTTCTTCCTAATC-3′	116
		R: 5′-GCAGTTTCCTGTGTCTTCTC-3′	
	*KLF10*	F: 5′-CTTCCGGGAACACCTGATTTT-3′	161
		R: 5′-GCAATGTGAGGTTTGGCAGTATC-3′	
	*MYC*	F: 5′-GGCTCCTGGCAAAAGGTCA-3′	119
		R: 5′-CTGCGTAGTTGTGCTGATGT-3′	
	*NKD2*	F: 5′-CCGACAGCAAACAGCAACT-3′	156
		R: 5′-AGCCTTAGAGCCAGGAAACA-3′	
	*GAPDH*	F: 5′-TGTTGCCATCAATGACCCCTT-3′	202
		R: 5′-CTCCACGACGTACTCAGCG-3′	

## Results

### Copy Number Alterations

**Fig 1:**
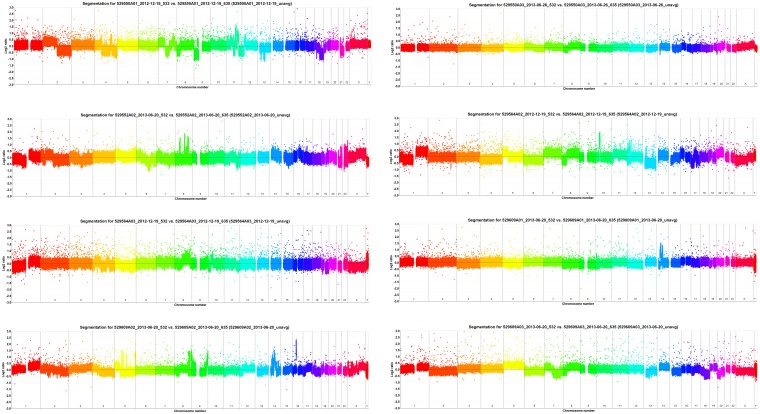
Array-CGH rainbows showed significant copy number heterogeneity across 8 paired LCXW samples.

Array-CGH detected 592 CNAs in the 8 paired LCXW samples (S1 Table). Copy number profiles were very heterogeneous: some cases showed multiple distinctive chromosomal aberrations, whereas others showed few chromosomal aberrations (Fig 1, S1–S8 Files).

### Gene Expression Profiling

**Fig 2:**
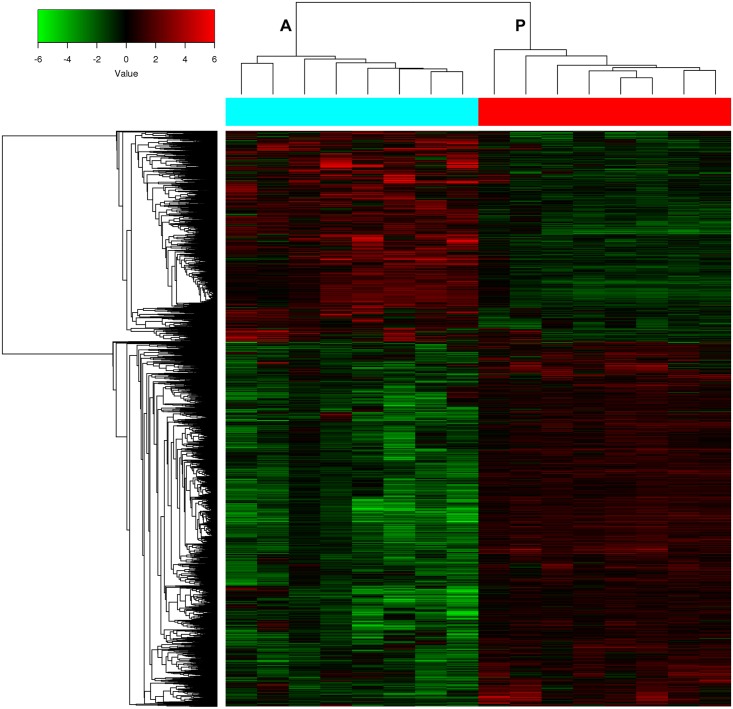
Hierarchical clustering of gene expression data showed a clear separation between the LCXW (A) and NCL tissues (P).

A total of 5,129 genes were identified as DEGs. Of these DEGs, 3,248 were upregulated while the other 1,881 genes were downregulated (S2 Table). Cluster analysis of these DEGs showed a distinct separation between the LCXW and NCL tissues (Fig 2). GO analysis indicated that these DEGs were involved in a wide range of cancer-related processes, including cell division, cell adhesion, cell proliferation and DNA replication. Pathway analysis showed these DEGs were involved in many pathways, such as those regulating p53 signaling, MAPK, Jak-STAT signaling, hedgehog signaling, and non-small cell lung cancer.

### Integrated Analysis of Copy Number Alterations and Gene Expression Profiling

To identify candidate CNAs from the 592 CNAs, sporadic CNAs among samples were removed from the dataset, leaving 95 recurrent CNAs detected in at least 3 samples (S3 Table). Among these 95 recurrent CNAs, 32 CNAs were inconsistent among samples and therefore were removed; thus, 63 concordant recurrent CNAs, comprising 56 gains and 7 losses, were identified (S4 Table). Among these 63 concordant recurrent CNAs, 14 contained no gene and 11 gains contained only partial gene segments; these CNAs were removed, revealing 38 candidate CNAs, including 34 gains and 4 losses (S5 Table). Of these candidate CNAs, the most frequent gains were 5p15.33-p15.32, 5p15.1-p14.3, and 5p14.3-p14.2, and the most frequent losses were 11q24.3, 21q21.1, 21q22.12-q22.13, and 21q22.2. These 38 candidate CNAs affected 246 genes, including protein-coding genes and hypothetical genes (S5 Table).

The integrated analysis of CNAs and gene expression results identified 24 genes (9.6%) that exhibited frequent copy number gains and concordant upregulation in the tumors (Table 3). A negative association between copy number and gene expression level was observed in 19 genes (7.7%) (Table 4), 15 genes (6.1%) that exhibited frequent copy number gains but were downregulated and 4 genes (1.6%) that were frequently deleted but upregulated. Review of the literature on these genes identified 3 genes in the positively correlated set (*CREB3L4*, *TRIP13*, and *CCNE2*) as potential oncogenes and 4 genes in the negatively correlated set (*AHRR*, *NKD2*, *MYC*, and *KLF10*) as potential tumor suppressor genes. KEGG pathway enrichment analysis revealed that the significantly enriched pathways were PI3K-Akt signaling (S1 Fig), prostate cancer (S2 Fig), cell cycle (S3 Fig), Wnt signaling (S4 Fig), and pathways in cancer (S5 Fig); this analysis also showed that *CCNE2*, *MYC*, and *CREB3L4* were the key involved genes. No pathway involving *TRIP13*, *AHRR*, or *KLF10* was found. The remaining 203 genes (82.5%) exhibited copy number changes, but no changes in transcript levels were observed in 49 of genes (19.9%) or detected in the other 154 genes (62.6%).

**Table 3:** Concordantly changed genes located in candidate copy number alterations.

Region	Cytoband	Size (bp)	Gain/Loss	Gene	Gene expression
chr1:152175304,152218160	1q21.3	42,857	gain	*CREB3L4*	up
chr5:98922,1202132	5p15.33	1,103,211	gain	*CEP72*	up
				*TRIP13*	up
chr5:1202132,4534212	5p15.33-p15.32	3,332,081	gain	*SLC6A3*	up
chr5:4534212,10760368	5p15.32-p15.2	6,226,157	gain	*ADAMTS16*	up
				*SRD5A1*	up
				*LOC442132*	up
				*DNAH5*	up
chr5:15167194,19455597	5p15.1-p14.3	4,288,404	gain	*BASP1*	up
chr5:21612069,24442605	5p14.3-p14.2	2,830,537	gain	*PMCHL1*	up
chr5:24442605,27519779	5p14.2-p14.1	3,077,175	gain	*LOC643401*	up
chr8:80437231,80753285	8q21.13	316,055	gain	*STMN2*	up
chr8:95372666,96348526	8q22.1	975,861	gain	*RAD54B*	up
				*INTS8*	up
				*CCNE2*	up
chr8:102462829,103474256	8q22.3	1,011,428	gain	*GRHL2*	up
chr8:104149770,104765560	8q22.3	615,791	gain	*CTHRC1*	up
				*RIMS2*	up
chr8:121617619,122330989	8q24.12	713,371	gain	*SNTB1*	up
chr8:124301834,124488436	8q24.13	186,603	gain	*ATAD2*	up
chr8:124529533,124985146	8q24.13	455,614	gain	*FBXO32*	up
				*ANXA13*	up
				*FAM91A1*	up
chr8:128501258,129666438	8q24.21	1,165,181	gain	*PVT1*	up

**Table 4:** Inconsistently changed genes located in candidate copy number alterations.

Region	Cytoband	Size (bp)	Gain/Loss	Gene	Gene expression
chr5:98922,1202132	5p15.33	1,103,211	gain	*AHRR*	down
				*TPPP*	down
				*NKD2*	down
chr5:4534212,10760368	5p15.32-p15.2	6,226,157	gain	*SEMA5A*	down
				*FAM105A*	down
chr5:15167194,19455597	5p15.1-p14.3	4,288,404	gain	*FBXL7*	down
chr8:79587922,80003750	8q21.12	415,829	gain	*PKIA*	down
chr8:82435662,82717833	8q21.13	282,172	gain	*FABP4*	down
chr8:102462829,103474256	8q22.3	1,011,428	gain	*NCALD*	down
chr8:103630996,103849228	8q22.3	218,233	gain	*KLF10*	down
chr8:107627585,108503880	8q23.1	876,296	gain	*ABRA*	down
				*ANGPT1*	down
chr8:108962975,109534011	8q23.1	571,037	gain	*RSPO2*	down
chr8:110496232,110689673	8q23.1-q23.2	193,442	gain	*PKHD1L1*	down
chr8:128501258,129666438	8q24.21	1,165,181	gain	*MYC*	down
chr21:36356593,36821536	21q22.12-q22.13	464,944	loss	*CBR1*	up
				*CBR3*	up
				*CHAF1B*	up
				*CLDN14*	up

### Validation of the Candidate Genes by RT-qPCR

The results of the RT-qPCR analysis of the 7 candidate genes in the 8 paired samples were consistent with the microarray results (data not shown), indicating that the microarrays were accurate. Further RT-qPCR analysis of copy number changes in the 7 candidate genes in the total paired patient samples showed that each gene had copy number gains in at least 40% (34–58) of the 84 (8+76) LCXW samples. Analysis of gene expression changes in the total patient samples showed that *CREB3L4*, *TRIP13*, and *CCNE2* were upregulated in at least 55% (32–40) of the 58 (8+50) LCXW samples, while *AHRR*, *NKD2*, *MYC*, and *KLF10* were downregulated in at least 48% (28–34) of the 58 LCXW samples (Table 5).

**Table 5:** Validation of copy number changes and expression of 7 candidate genes.

Gene	Copy number validation (N = 84)		Gene expression validation (N = 58)	
	Gain / Loss	n (%)	Upregulation / Downregulation	n (%)
*CREB3L4*	Gain	40 (48%)	Upregulation	32 (55%)
*TRIP13*	Gain	58 (69%)	Upregulation	40 (69%)
*CCNE2*	Gain	46 (55%)	Upregulation	40 (69%)
*AHRR*	Gain	34 (40%)	Downregulation	32 (55%)
*NKD2*	Gain	52 (62%)	Downregulation	28 (48%)
*MYC*	Gain	40 (48%)	Downregulation	30 (52%)
*KLF10*	Gain	42 (50%)	Downregulation	34 (59%)

## Discussion

This integrated analysis of genomic DNA CNAs and gene expression profiling in LCXW and paired normal tissue was designed to screen potential novel “driver genes” in LCXW. Overall, the 38 candidate CNAs featured more gains than losses (34 vs. 4). The recurrent gains were located mainly on chromosomes 5p, 8q, 7p, and 1q, and losses were located on 21q and 11q. Comparison of these results with reported data for lung adenocarcinoma [12–14] identified Amp_5p15.33, Amp_7p11.2, and Amp_8q24.21 as common recurrent CNAs in all the studies (Table 6), suggesting that these regions may be variant hotspots in lung adenocarcinoma. CNAs, such as Del_9p21.3 and Amp_14q13.3 that have been reported to have the highest mutation frequencies in lung adenocarcinoma [12–14] were not identified in our LCXW samples and, similarly, many concordant recurrent CNAs detected in our study did not overlap with the reported data [12–14] (Table 6), suggesting that genomic copy number changes in LCXW may differ from those of other lung cancers. Because of the small sample size in our study, however, further studies are needed to determine the characteristic CNAs in LCXW.

**Table 6:** Comparison of concordant recurrent CNAs with literature.

This study		Staaf et al [12]		The Cancer Genome Atlas Research Network [13]		Barbara et al [14]		Top candidate gene
CNA	Region (bp)	CNA	Region (bp)	CNA	Region (bp)	CNA	Region (Mb)	
Amp_1q21.3	chr1:152175304–152218160			Amp_1q21.3	chr1:120523956–152743148			*CREB3L4*
Amp_5p15.33	chr5:98922–1202132	Amp_5p15.33	chr5:120000–1686000					*AHRR*, *TRIP13*, *NKD2*
Amp_5p15.33-p15.32	chr5:1202132–4534212	Amp_5p15.33	chr5:120000–1686000	Amp_5p15.33	chr5:1288616–1300024	Amp_5p15.33	chr5:0.75–1.62	*TERT*, *SLC6A3*
Amp_5p15.32-p15.2	chr5:4534212–10760368					Amp_5p15.31	chr5:8.88–10.51	
Amp_5p15.1-p14.3	chr5:15167194–19455597							
Amp_5p14.3	chr5:19455597–20320969					Amp_5p14.3	chr5:19.72–23.09	
Amp_5p14.3	chr5:21488497–21612069					Amp_5p14.3	chr5:19.72–23.09	
Amp_5p14.3-p14.2	chr5:21612069–24442605					Amp_5p14.3	chr5:19.72–23.09	
Amp_5p14.2-p14.1	chr5:24442605–27519779							
Amp_7p22.3	chr7:136363–478785							
Amp_7p11.2	chr7:54989787–55769659	Amp_7p11.2	chr7:54795000–55455000	Amp_7p11.2	chr7:54535672–55737616	Amp_7p11.2	chr7:54.65–55.52	*EGFR*
Amp_8q21.12	chr8:79282515–79584188							
Amp_8q21.12	chr8:79587922–80003750							
Amp_8q21.12-q21.13	chr8:80175394–80433401							
Amp_8q21.13	chr8:80437231–80753285					Amp_8q21.13	chr8:80.66–82.55	
Amp_8q21.13	chr8:80756352–81238430					Amp_8q21.13	chr8:80.66–82.55	
Amp_8q21.13	chr8:81592461–81654094					Amp_8q21.13	chr8:80.66–82.55	
Amp_8q21.13	chr8:81718686–82433172					Amp_8q21.13	chr8:80.66–82.55	
Amp_8q21.13	chr8:82435662–82717833					Amp_8q21.13	chr8:80.66–82.55	
Amp_8q22.1	chr8:95372666–96348526							*CCNE2*, *RAD54B*
Amp_8q22.3	chr8:102462829–103474256	Amp_8q22.3	chr8:102908001–103565001					*GRHL2*, *NCALD*
Amp_8q22.3	chr8:103630996–103849228							*KLF10*
Amp_8q22.3	chr8:103856352–104144193							
Amp_8q22.3	chr8:104149770–104765560							*CTHRC1*
Amp_8q23.1	chr8:107627585–108503880							
Amp_8q23.1	chr8:108962975–109534011							
Amp_8q23.1	chr8:110143020–110414628							
Amp_8q23.1-q23.2	chr8:110496232–110689673							
Amp_8q23.2	chr8:110697169–110915327							
Amp_8q24.11	chr8:118584139–118626715							
Amp_8q24.12	chr8:121617619–122330989							
Amp_8q24.13	chr8:124301834–124488436							
Amp_8q24.13	chr8:124529533–124985146							*ANXA13*
Amp_8q24.21	chr8:128501258–129666438	Amp_8q24.21	chr8:128729001–128873001	Amp_8q24.21	chr8:129157821–129195260	Amp_8q24.21	chr8:129.18–129.34	*MYC*, *PVT1*
Del_11q24.3	chr11:129705556–129762130	Del_11q24.3-q25	chr11:127528001–131659001					
Del_21q21.1	chr21:17559651–17823071			Del_21q21.1	chr21:1–32497730			
Del_21q22.12-q22.13	chr21:36356593–36821536							*CHAF1B*, *CBR1*
Del_21q22.2	chr21:39568333–39679987							

In carrying out this analysis, the initial array-CGH detected a large number of CNAs. To screen out the best candidate CNAs, we filtered out the CNAs that were inconsistent between samples, the CNAs without a gene, and the gains containing only a partial gene segment. Through this process of elimination, 38 concordant recurrent CNAs were selected. This approach, on the one hand, may be an effective screening method for vital CNAs; on the other hand, focusing only on concordant recurrent CNAs may exclude important sporadic CNAs that may have a role in the cancerous phenotype of interest.

A total of 246 genes were located in the 38 candidate CNAs. Of these, only 24 genes were upregulated and concordantly increased in copy number, and none was downregulated with loss in copy number. In fact, the change in expression in many genes was inconsistent with the copy number change, in some cases even showing negative correlation. Of the 19 negatively correlated genes, 15 genes located in copy number gains were significantly downregulated, and the other 4 genes located in copy number losses were significantly upregulated. This paradoxical negative relationship between copy number status and gene expression has also been observed in other cancers [10]. It might be attributable to the multiple mechanisms that are responsible for normal and abnormal control of gene expression, including those related to gene mutation, promoter methylation, and non-coding RNA regulation. Overall, the upregulated genes represent potential candidate oncogenes, while the downregulated genes represent potential candidate tumor suppressor genes in LCXW. There were 49 genes with copy number changes, including some known cancer-related genes, such as *TERT* and *EGFR*, that did not show expression changes, suggesting that their expression may be not gene-dose dependent and that they are likely to be passenger genes or play a role in LCXW carcinogenesis in other ways. The remaining 154 genes, including a large number of hypothetical genes, were undetected by microarrays, and thus, they were removed from the analysis.

Integrated analysis of CNAs and corresponding DEGs has been shown to be an effective approach to identify genes with altered copy numbers directly impacting on the expression levels [9,10], however, not all of these genes are cancer-related. In order to further narrowing the scope of candidate genes, the literature on the 43 positively or negatively correlated genes were reviewed, then we selected 7 genes, including 3 positively correlated genes (*CREB3L4*, *TRIP13*, and *CCNE2*) and 4 negatively correlated genes (*AHRR*, *NKD2*, *MYC*, and *KLF10*) as candidate driver genes, which were further validated by RT-qPCR. KEGG pathway enrichment analysis showed that *CCNE2*, *MYC*, *CREB3L4*, and *NKD2* are involved in many tumor-related pathways, suggesting that these genes may play an essential role in cancer development.

*CREB3L4* (cAMP responsive element binding protein 3-like 4) is located on chromosome 1q21.3 and encodes a cAMP responsive element binding protein which functions in a number of processing pathways, such as transcriptional regulation, signal transduction, and cell homeostasis. *CREB3L4* has been shown to be associated with the development of cancers [15, 16]. *CREB3L4* is upregulated in both a prostate cancer cell line (LNCaP) and in primary prostate cancer cells. In addition, the 1q21 amplicon containing *CREB3L4* is frequently detected in hepatocellular carcinoma, and *CREB3L4* is significantly overexpressed in tumor tissues compared with nontumorous tissue counterparts [16]. *TRIP13* (thyroid hormone receptor interactor 13) encodes a protein that is a novel mitotic checkpoint-silencing protein and plays centrally important roles in mitotic checkpoint complex (MCC) disassembly and checkpoint inactivation. *TRIP13* knockdown can delay metaphase-to-anaphase transition, while *TRIP13* overexpression can trigger premature mitotic checkpoint silencing and thereby promote cancer development [17]. Overexpression of *TRIP13* has been shown to result in malignant transformation of non-malignant cells and high expression of *TRIP13* in squamous cell carcinoma of the head and neck can lead to aggressive, treatment-resistant tumors and enhanced repair of DNA damage [18]. *CCNE2* (cyclin E2) specifically interacts with the CIP/KIP family of CDK inhibitors and plays a role in cell cycle G1/S transition. Elevated *CCNE2* level can lead to genomic instability such as increased proportion of abnormal mitoses, micronuclei, and chromosomal aberrations [19]. Significantly increased expression levels of *CCNE2* have been observed in various tumors such as those of the lung, breast, pancreas, and nasopharyngx, and have been shown to play important roles in the proliferation, invasion, metastasis, and poor prognosis of these cancers [20, 21]. The copy number gains and upregulation of expression of *CREB3L4*, *TRIP13* and *CCNE2* in more than 55% of our LCXW samples suggest that their expression might be gene-dose sensitive and that they are potential oncogenes in LCXW. To the best of our knowledge, expression changes in neither *CREB3L4* nor *TRIP13* have been reported in lung cancer, suggesting previously unknown associations with LCXW and lung cancer in general.

*AHRR* (aryl-hydrocarbon receptor repressor) encodes a protein participating in the aryl hydrocarbon receptor (AhR) signaling cascade, which mediates dioxin toxicity and is involved in regulation of cell growth and differentiation. AHRR functions as a feedback modulator by repressing AhR-dependent gene expression. The genetic polymorphisms in *AHRR* have been shown to be risk factors for cancer via ameliorating this AhR repressor activity [22, 23], and DNA methylation change in *AHRR* has been linked to smoking exposure and lung cancer [24]. Thus, *AHRR* has been proposed to function as a putative new tumor suppressor gene in multiple types of human cancers [25]. *NKD2* (naked cuticle homolog 2) encodes a protein that participates in the delivery of transforming growth factor alpha (TGFα)-containing vesicles and functions as a negative regulator of Wnt receptor signaling through interaction with members of the Dishevelled family. Downregulation of *NKD2* is frequently regulated by hypermethylation of the promoter region and can cause Wnt activation and TGFα misdelivery, which often leads to tumorigenesis [26–28]. *KLF10* (Kruppel-like factor 10) encodes a transcriptional repressor that acts as an effector of TGF-β signaling. KLF10 functions as a toggle by differential coupling of Sin3-histone deacetylase and P300/PCB-associated factor to integrate antagonistic signals regulating FOXP3, resulting in immune activation, and it also can directly bind to the TGF-β RII promoter in CD8(+)T cells, leading to enhanced gene expression and tumor immune response. *KLF10* can inhibit breast cancer invasion and metastasis by inhibiting epidermal growth factor receptor (EGFR) transcription and the EGFR signaling pathway [29]. The expression of *KLF10* is inversely correlated with pancreatic cancer stage, prognosis and overall survival [30]. In our study, the copy number of *AHRR*, *NKD2*, and *KLF10* increased in at least 40% of the LCXW samples (34/84), whereas their expression was downregulated in at least 48% (28/58), suggesting that their expression is not gene-dose dependent, and if decreased, might promote the development of LCXW. To the best of our knowledge, expression changes in neither *NKD2* nor *KLF10* have been reported in lung cancer, suggesting that these genes are previously unknown tumor suppressor genes in LCXW and in lung cancer in general.

*MYC* (v-myc avian myelocytomatosis viral oncogene homolog) encodes a multifunctional nuclear phosphoprotein that plays a role in cell cycle progression, apoptosis, and cellular transformation and regulates transcription of specific target genes. *MYC*, one of the most studied oncogenes [31], is typically overexpressed in variety of malignant tumors such as lung cancer, lymphomas, breast cancer, gastric cancer, and colon cancer and is involved in cell proliferation, differentiation, apoptosis and cell cycle [32–37]. Unexpectedly, the expression of *MYC* was significantly decreased in 52% (30/58) of the LCXW samples tested, although its copy number increased in 48% (40/84) of the LCXW samples tested, which indicates that *MYC* may not play a carcinogenic role in LCXW. This might reflect one aspect of the different pathogenesis of LCXW and lung cancers in other geographic areas.

In conclusion, this study provided an integrative analysis of genome-wide DNA CNAs and gene expression to identify candidate driver genes in LCXW. Our findings suggest that *CREB3L4*, *TRIP13*, and *CCNE2* are potential oncogenes, *AHRR*, *NKD2*, and *KLF10* are potential tumor suppressor genes in LCXW, while *MYC*, one of the most studied oncogenes, might not play a carcinogenic role in LCXW. These discoveries will help us understand the pathogenesis and provide novel potential therapeutic targets for LCXW.

## Supporting Information

S1 File Multi_panel array-CGH result of sample 529550A01. (PDF)

S2 File Multi_panel array-CGH result of sample 529550A02. (PDF)

S3 File Multi_panel array-CGH result of sample 529550A03. (PDF)

S4 File Multi_panel array-CGH result of sample 529552A02. (PDF)

S5 File Multi_panel array-CGH result of sample 529564A02. (PDF)

S6 File Multi_panel array-CGH result of sample 529564A03. (PDF)

S7 File Multi_panel array-CGH result of sample 529609A02. (PDF)

S8 File Multi_panel array-CGH result of sample 529609A03. (PDF)

S1 Fig *CCNE2*, *MYC*, and *CREB3L4* were involved in the PI3K-Akt signaling pathway. (TIF)

S2 Fig *CCNE2* and *CREB3L4* were involved in the prostate cancer signaling pathway. (TIF)

S3 Fig *CCNE2* and *MYC* were involved in the cell cycle signaling pathway. (TIF)

S4 Fig *MYC* and *NKD2* were involved in the Wnt signaling pathway. (TIF)

S5 Fig *CCNE2* and *MYC* were involved in the pathways in cancer. (TIF)

S1 Table Copy number alterations in lung cancer in Xuanwei identified by array-CGH analysis. (XLSX)

S2 Table Differentially expressed genes in lung cancer in Xuanwei identified by microarray analysis. (XLSX)

S3 Table The recurrent CNAs presented in at least 3 samples. (XLSX)

S4 Table The concordant recurrent CNAs presented in at least 3 samples. (XLSX)

S5 Table 38 candidate CNAs and the expression changes of genes located in these CNAs. (XLSX)

## Abbreviations

array-CGH: array-based comparative genomic hybridization
CNAs: copy number alterations
DEGs: differentially expressed genes
LCXW: lung cancer in Xuanwei
MCC: mitotic checkpoint complex
NCL: non-cancerous lung
PAHs: polycyclic aromatic hydrocarbons
RT-qPCR: real-time quantitative polymerase chain reaction
