# Supplementary figures and images for: Integrated Analysis of Genome-Wide Copy Number Alterations and Gene Expression Profiling of Lung Cancer in Xuanwei, China
Source: PLoS One. 2017 Jan 5;12(1):e0169098. doi: 10.1371/journal.pone.0169098 (PMC5215791; doi:10.1371/journal.pone.0169098)

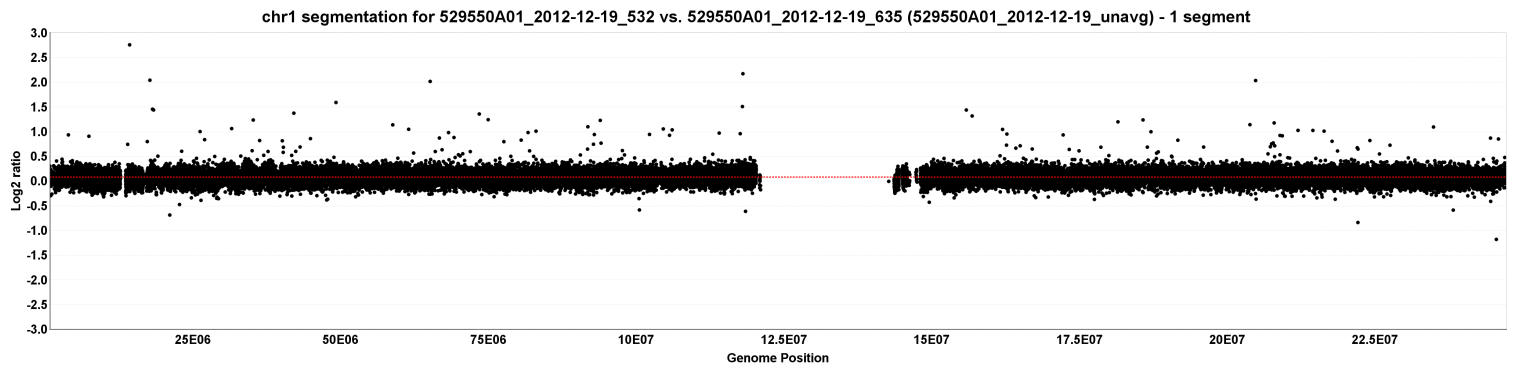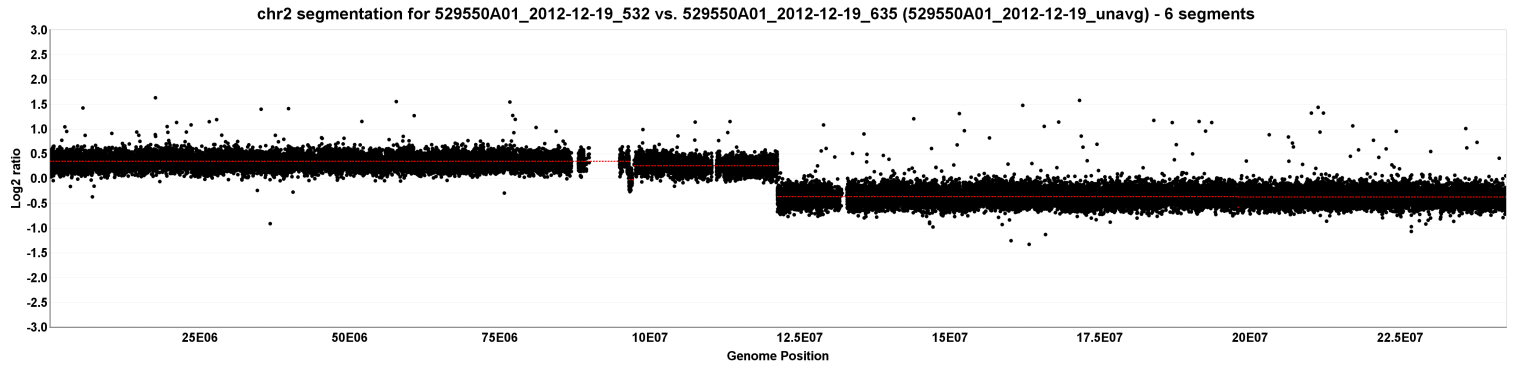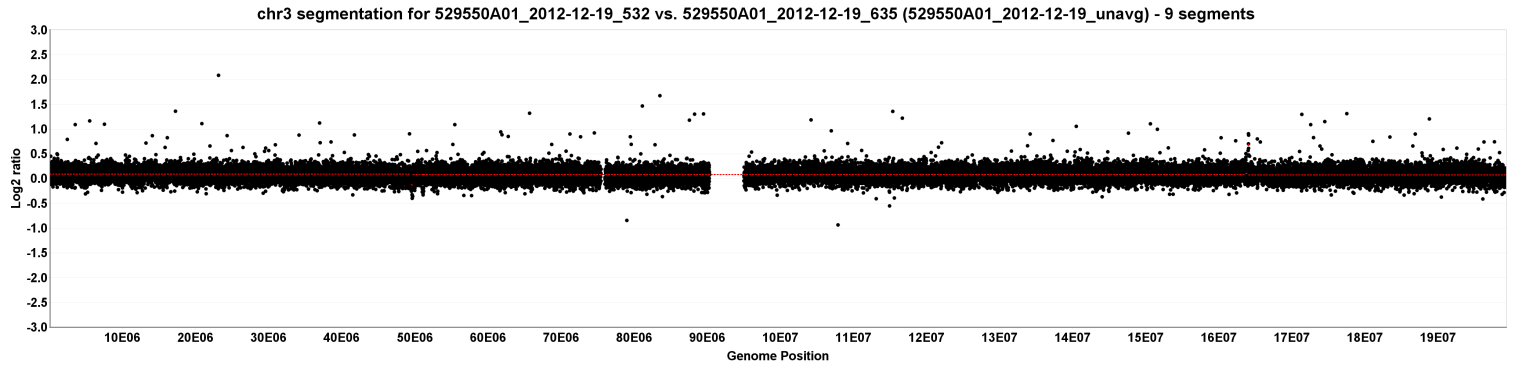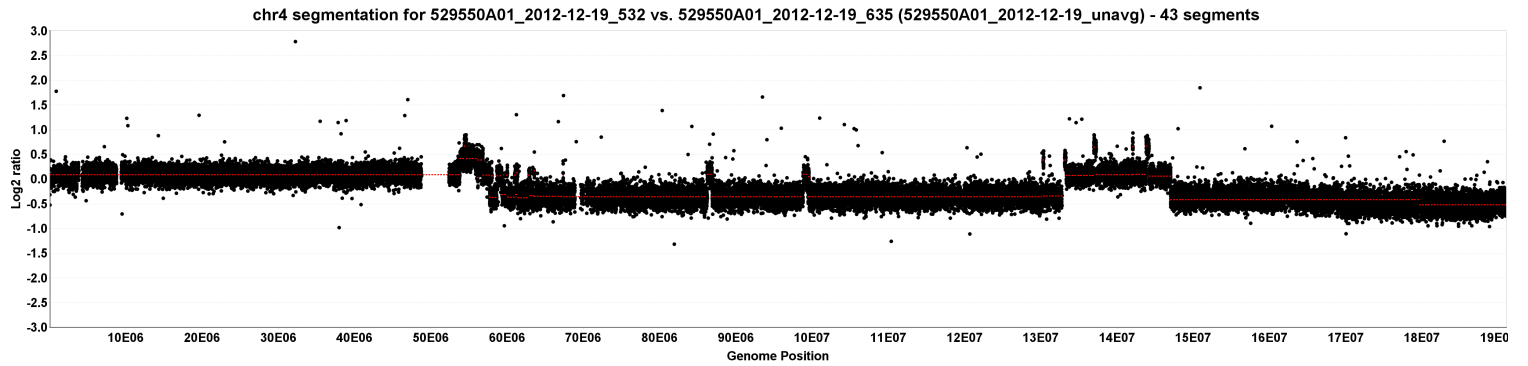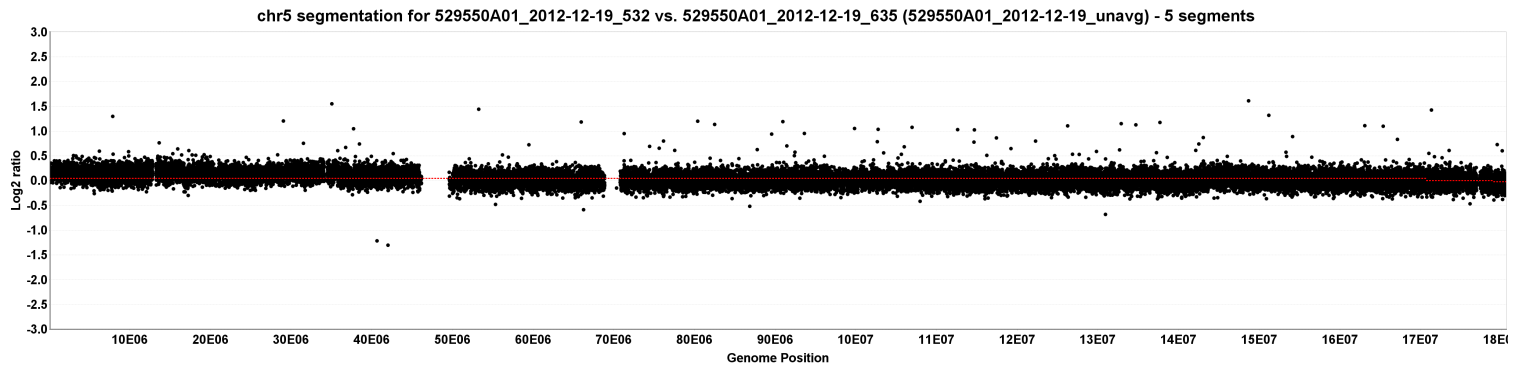

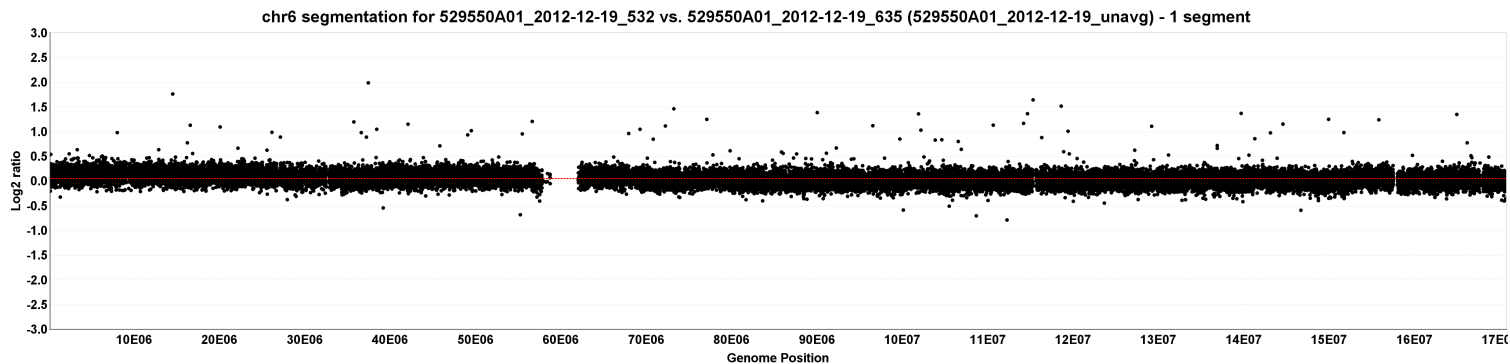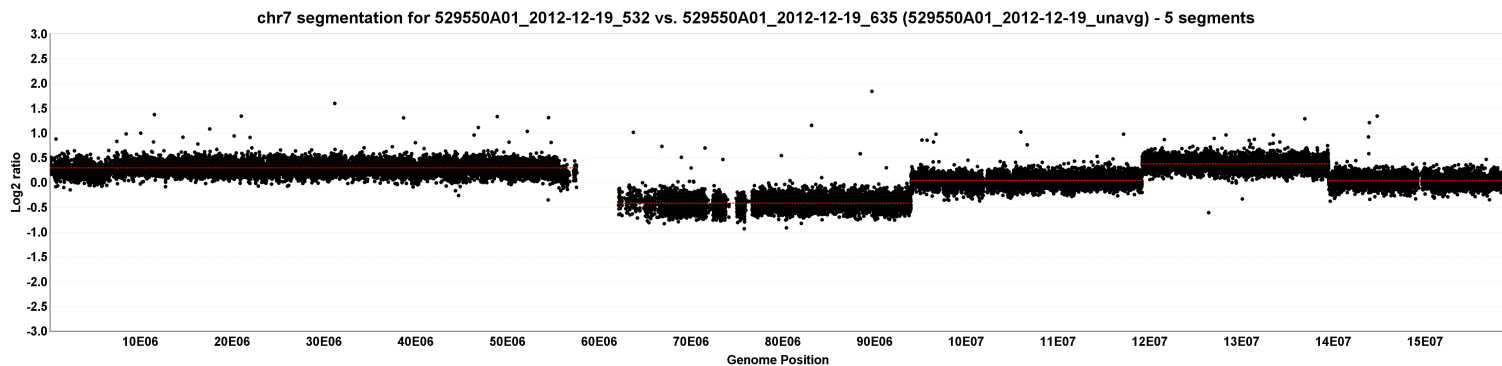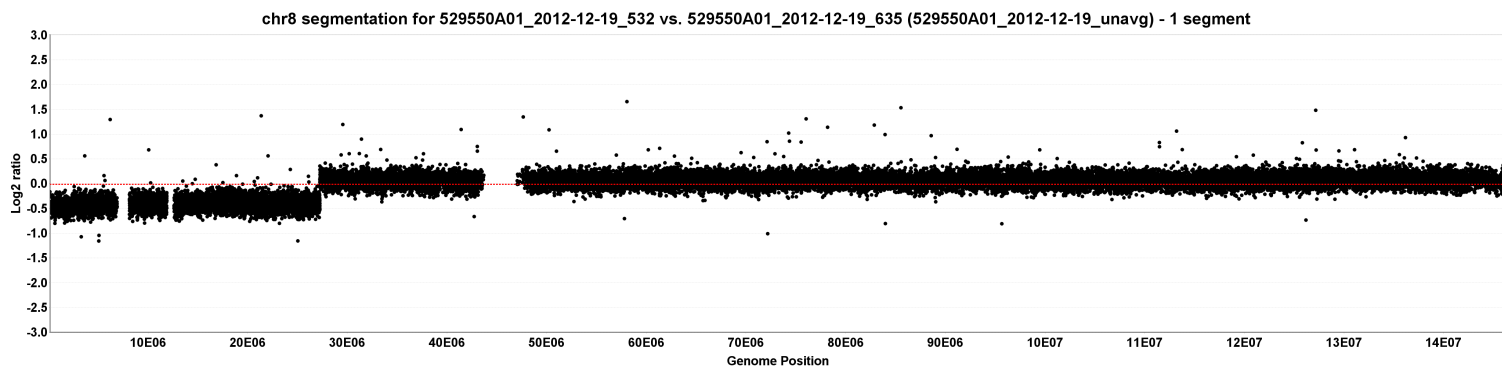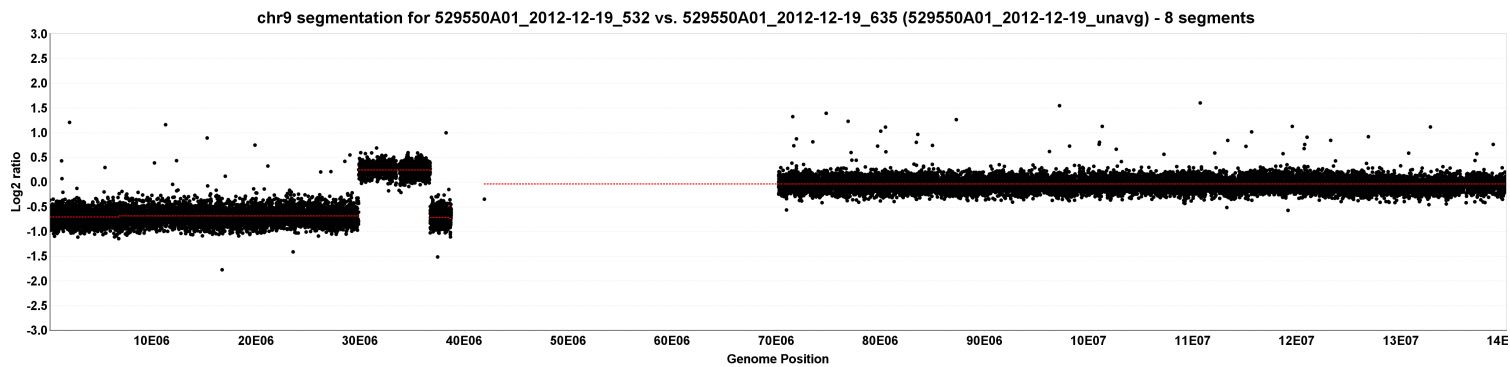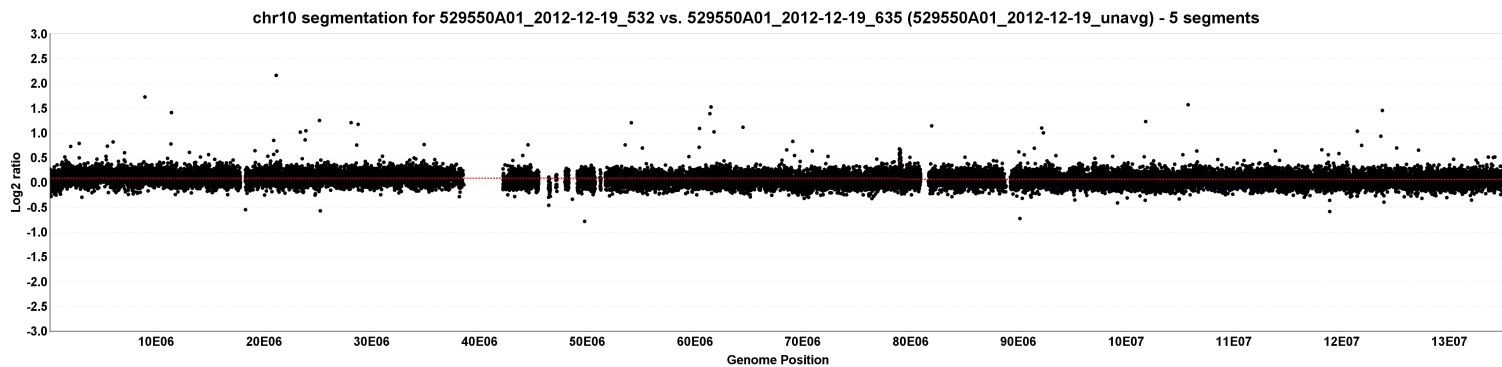

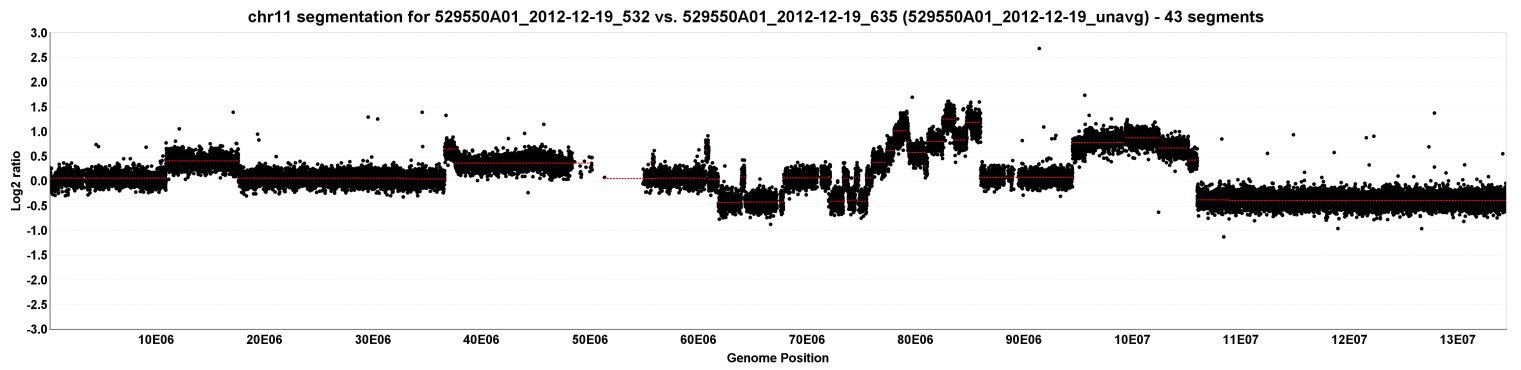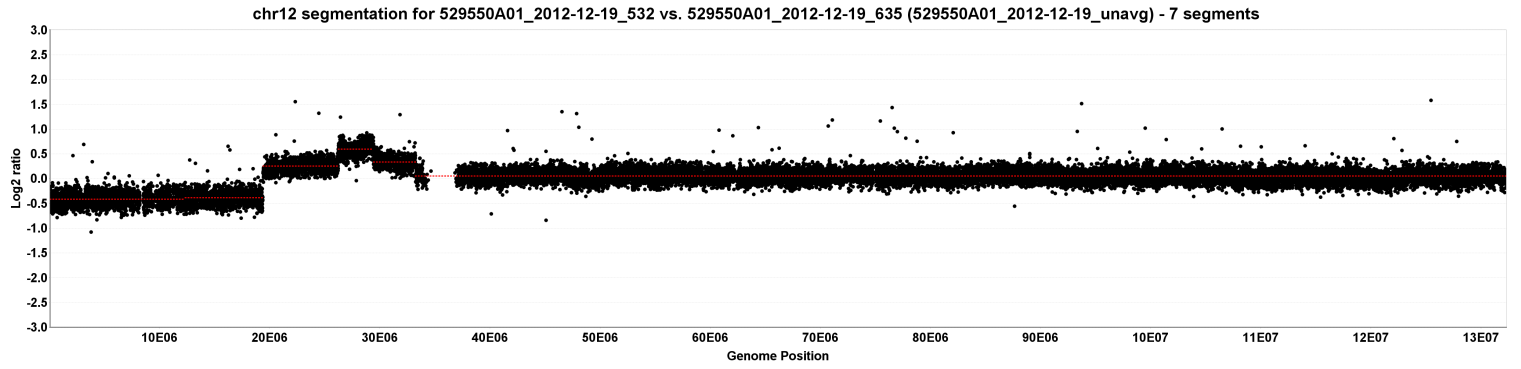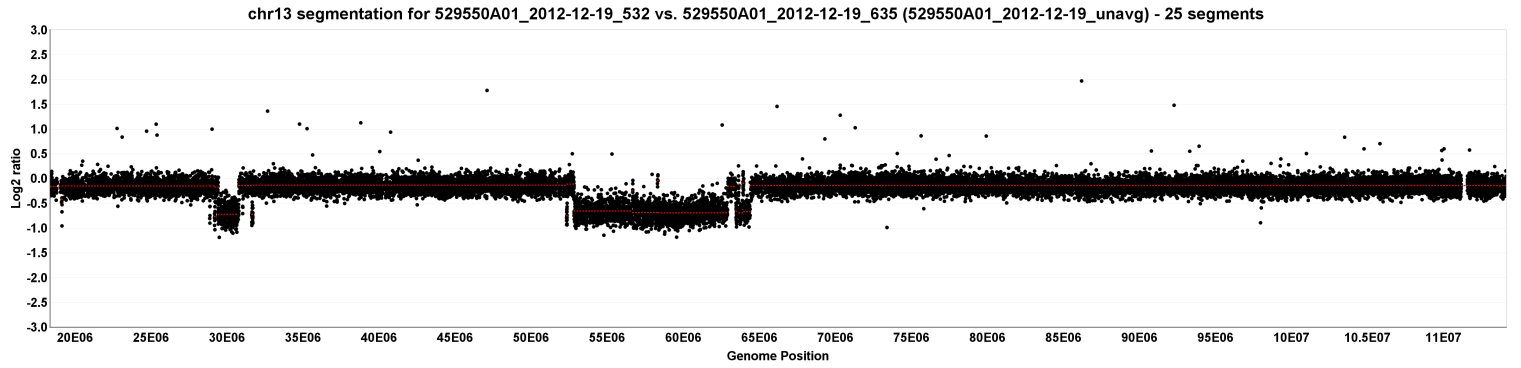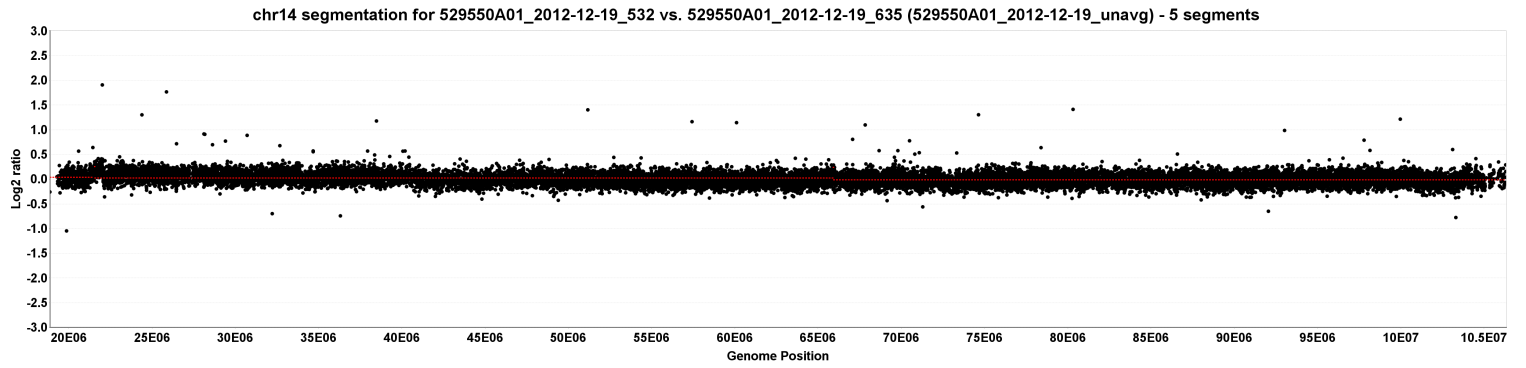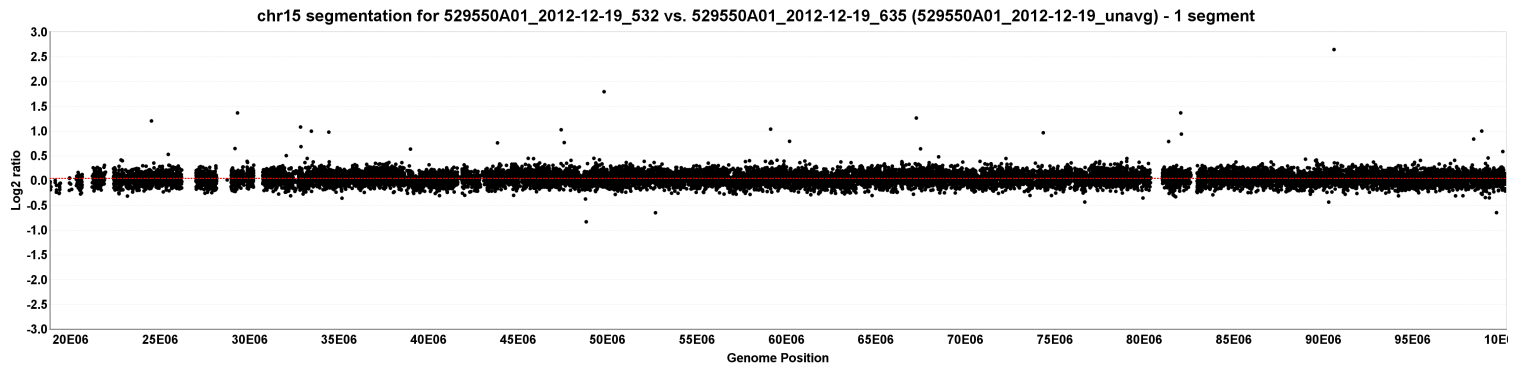

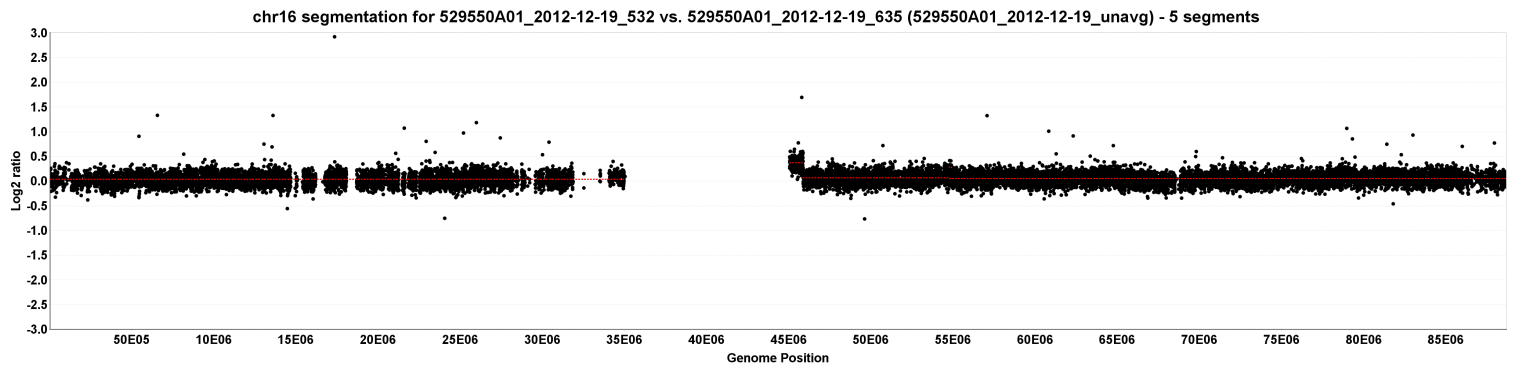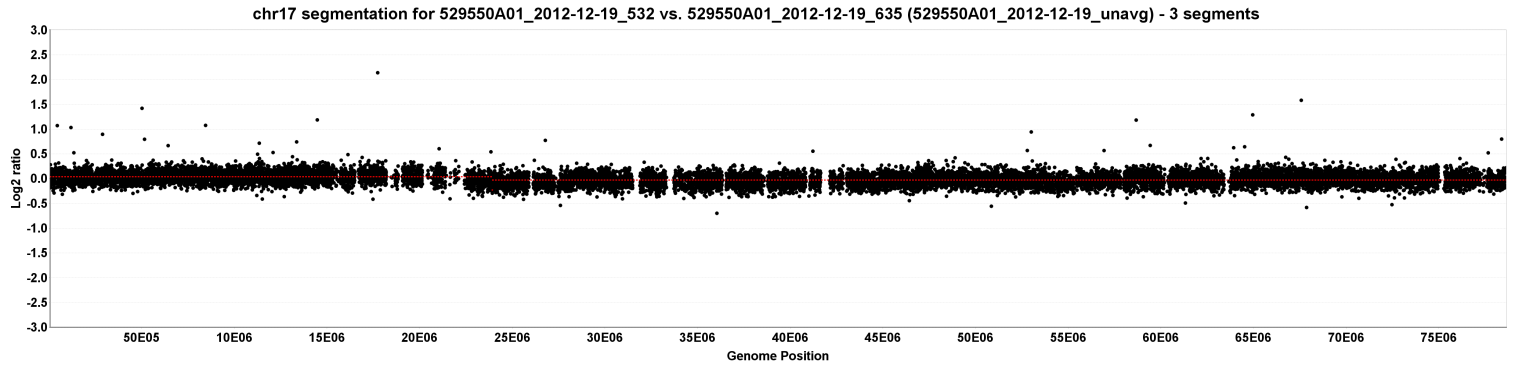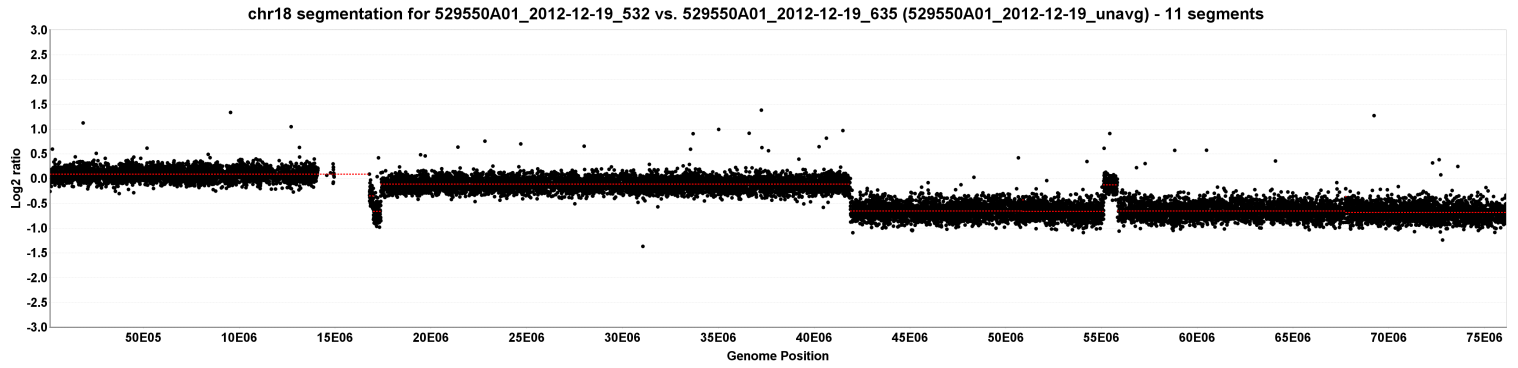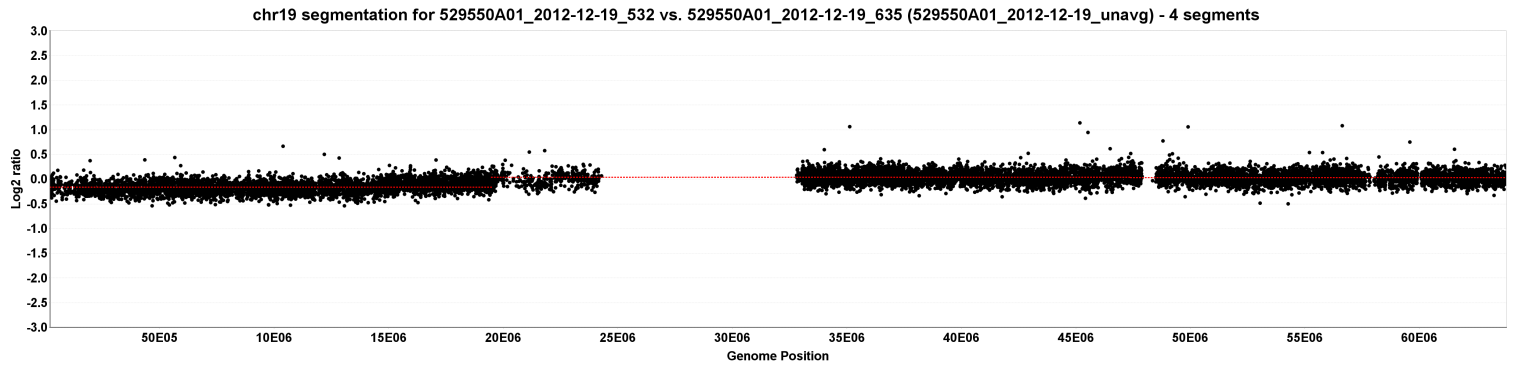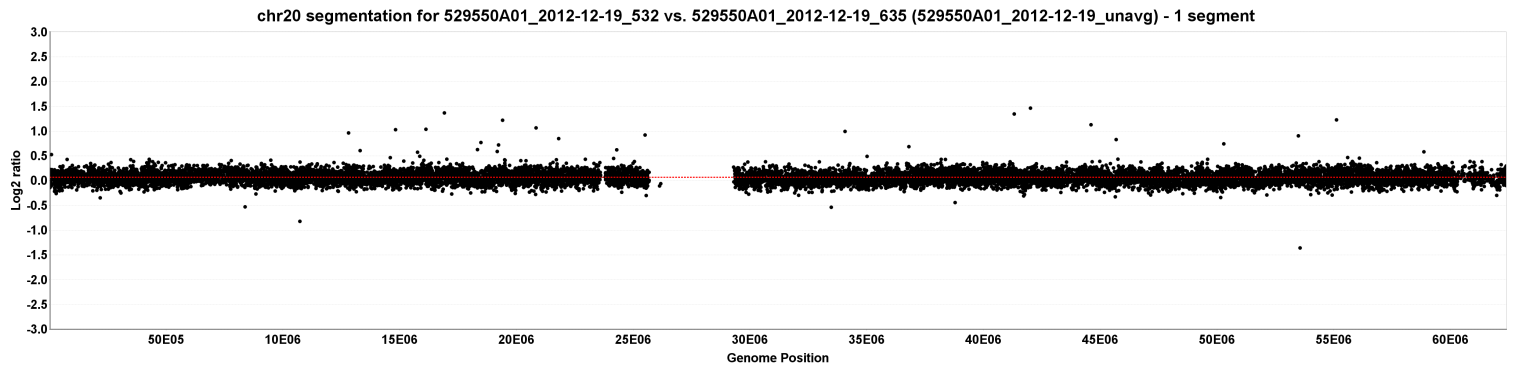

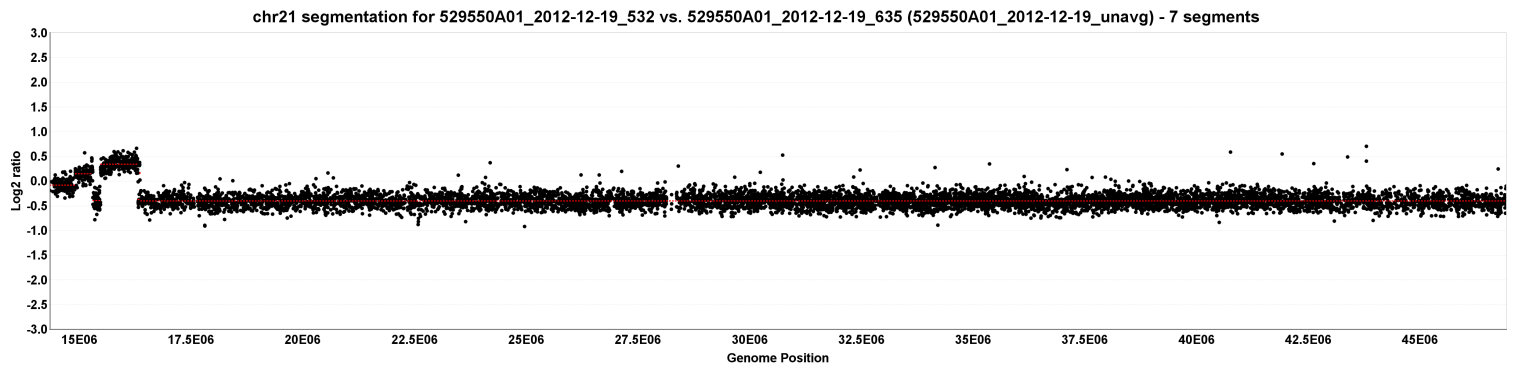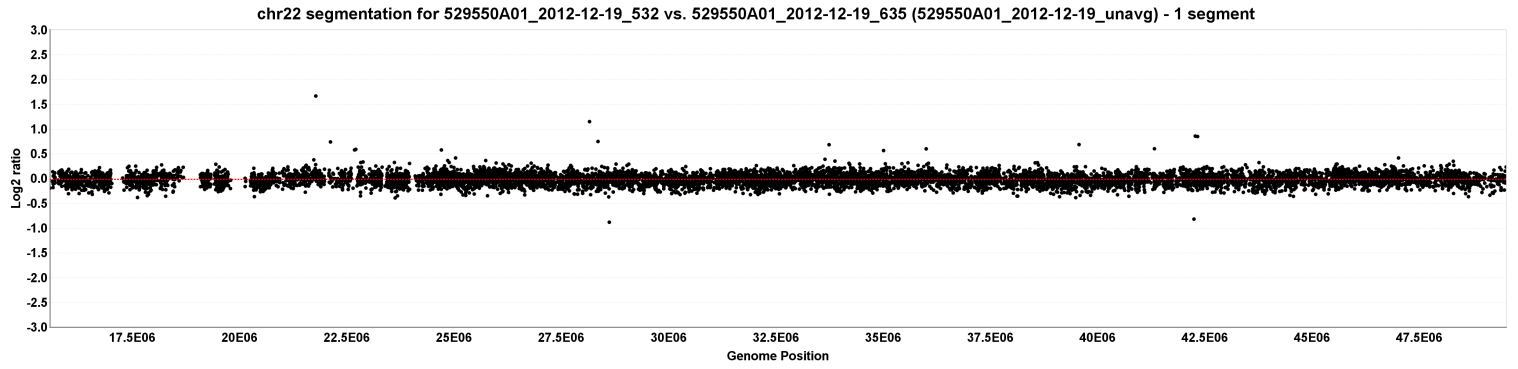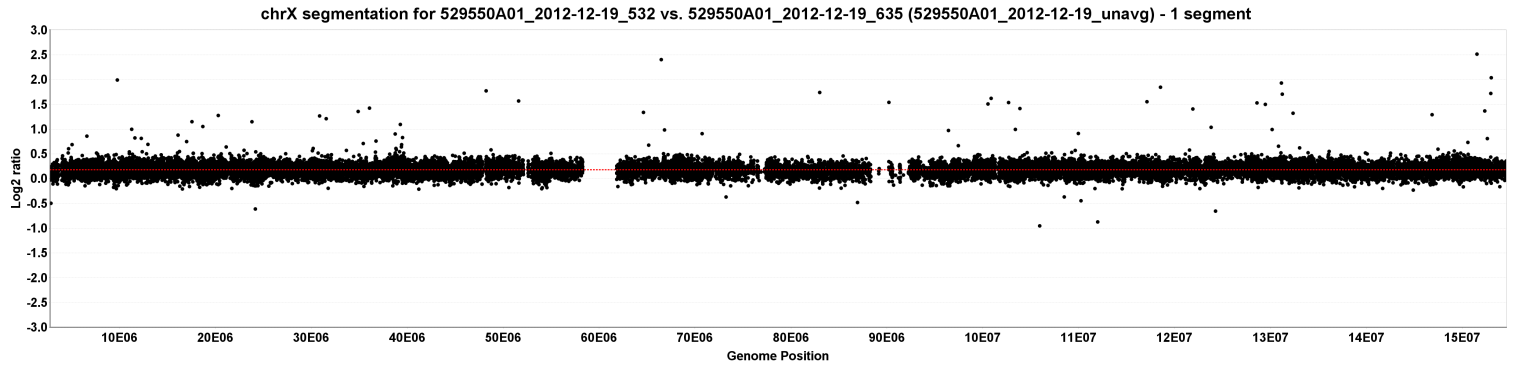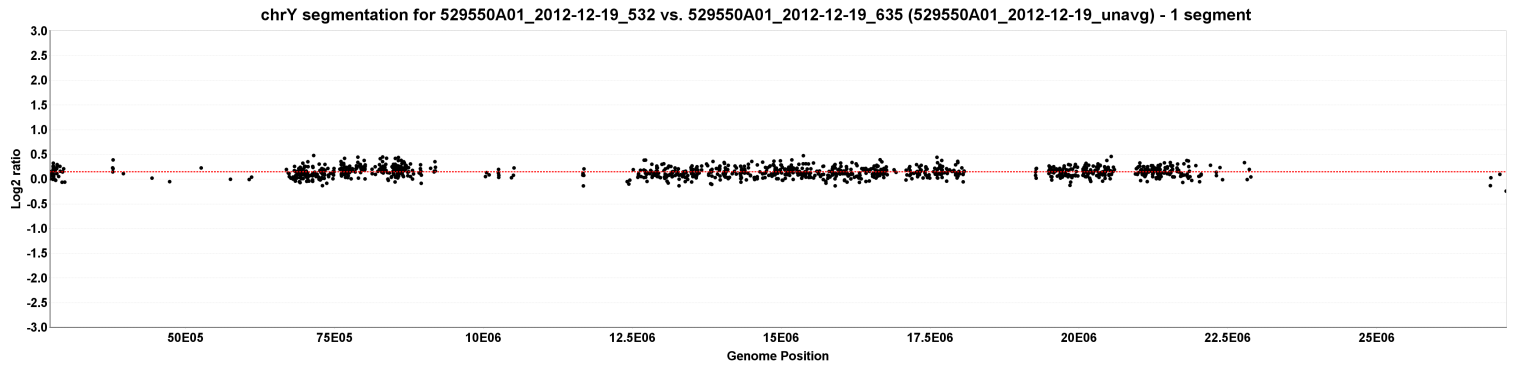

Supplement: S1 File — (PDF) [file pone.0169098.s001.pdf]

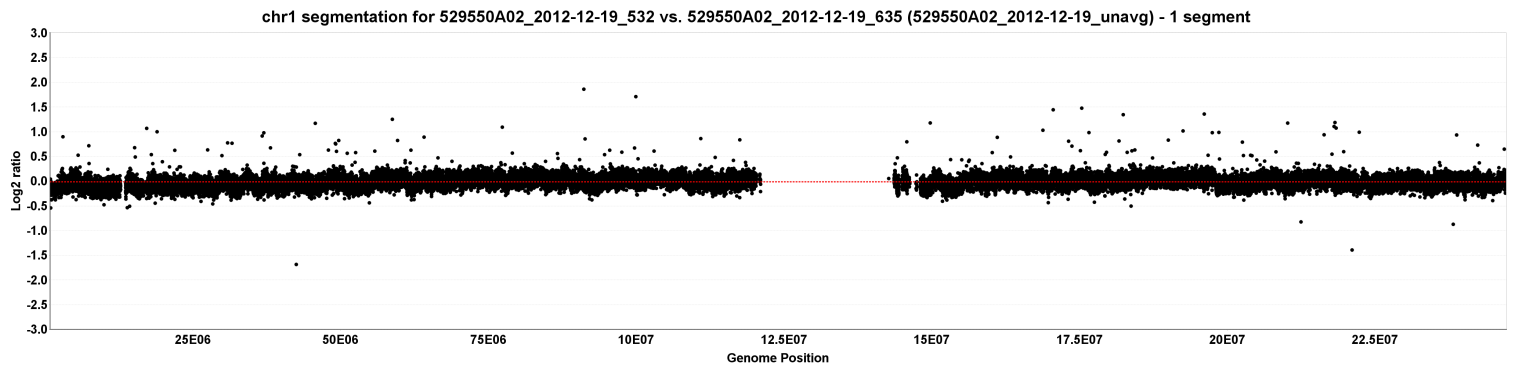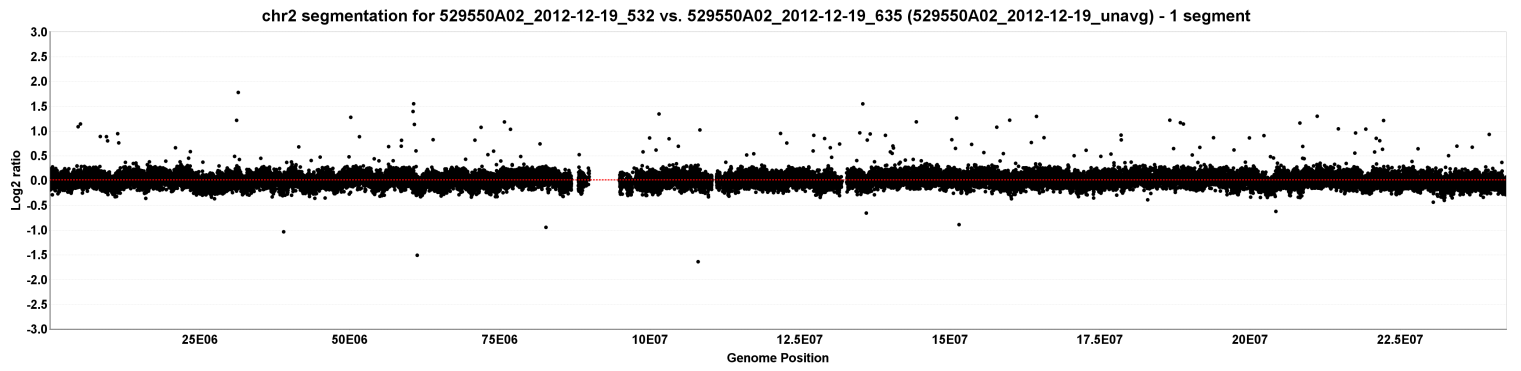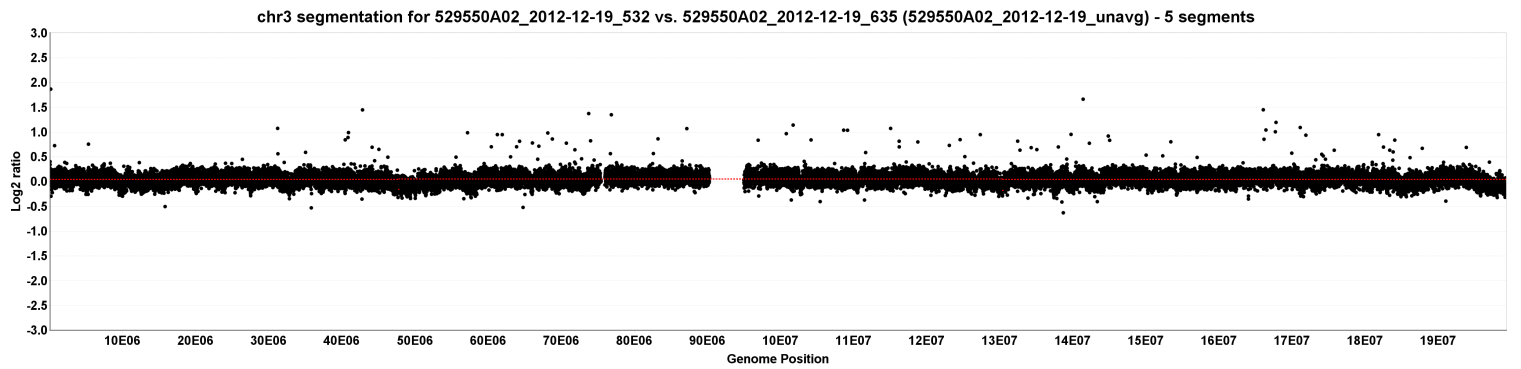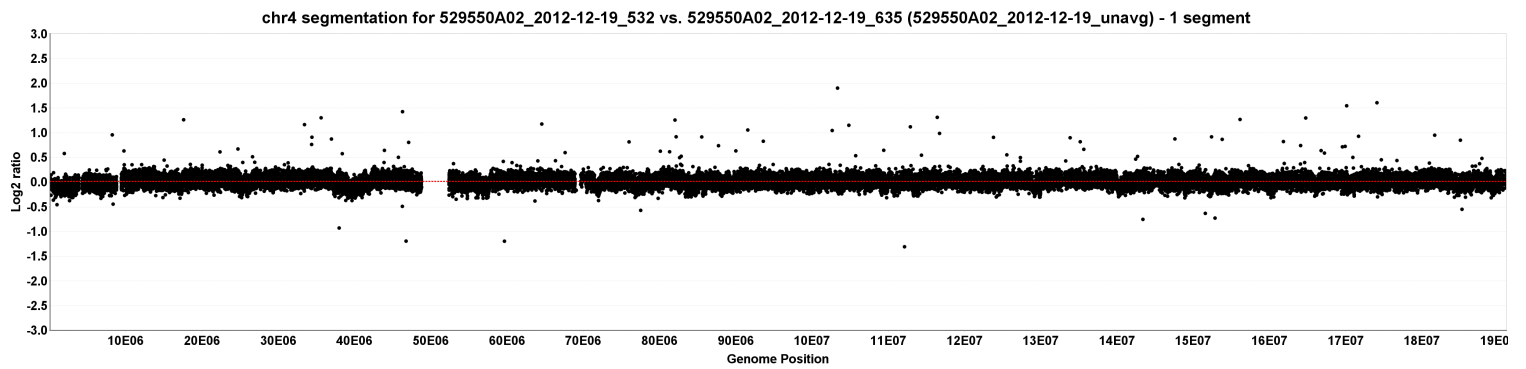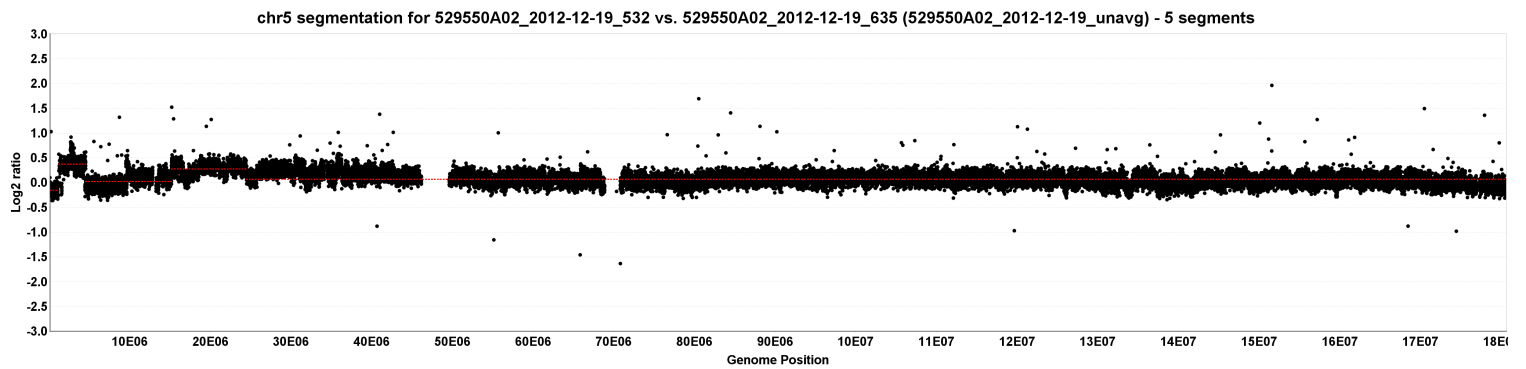

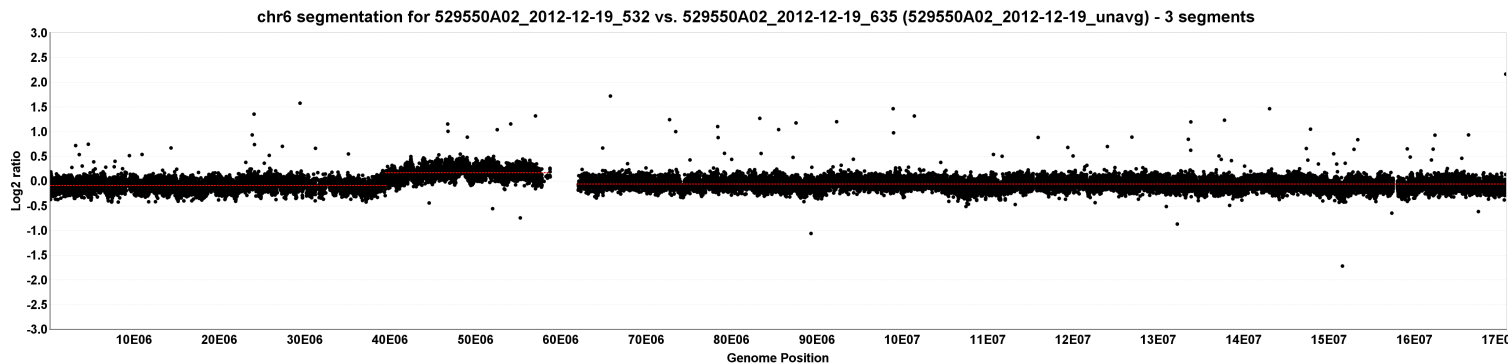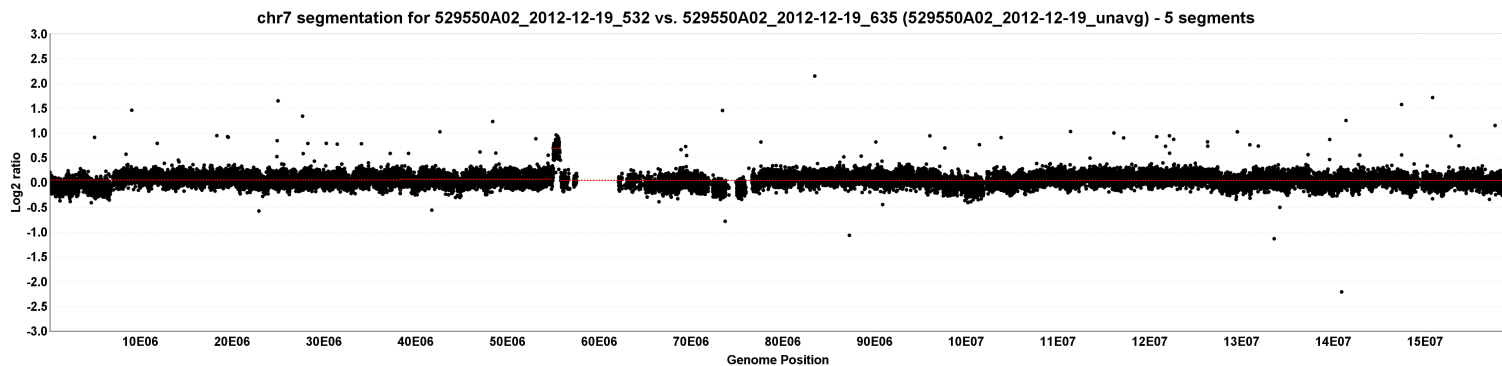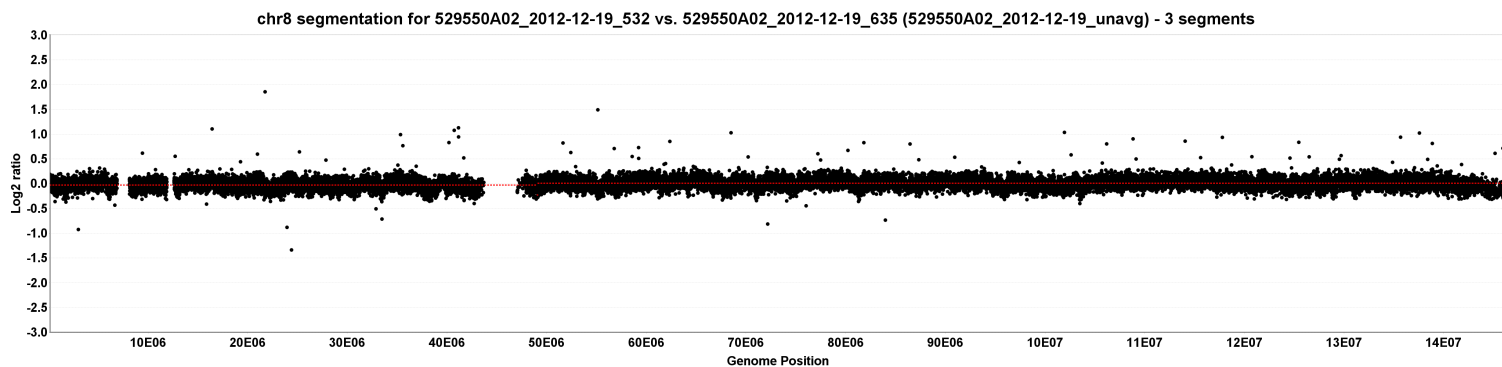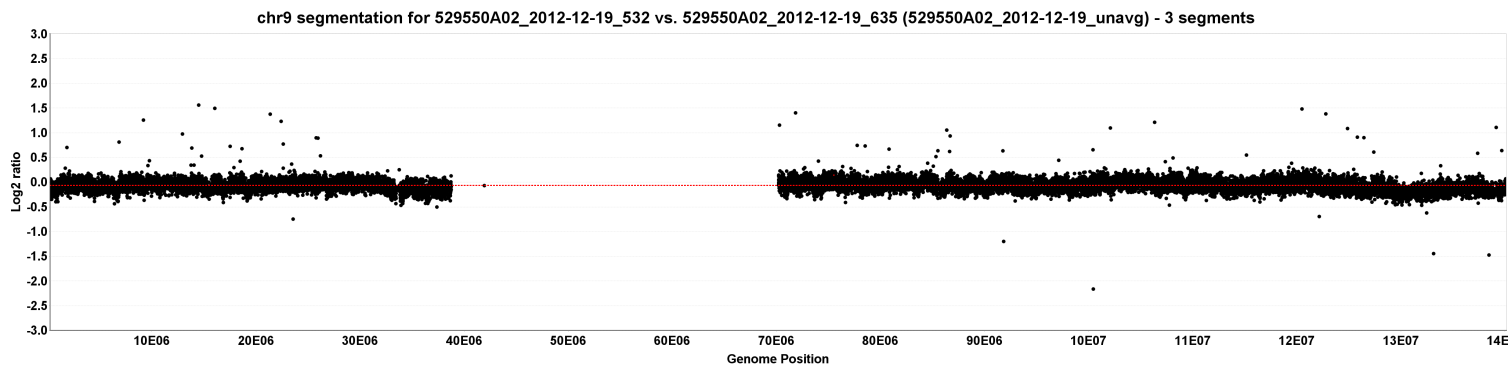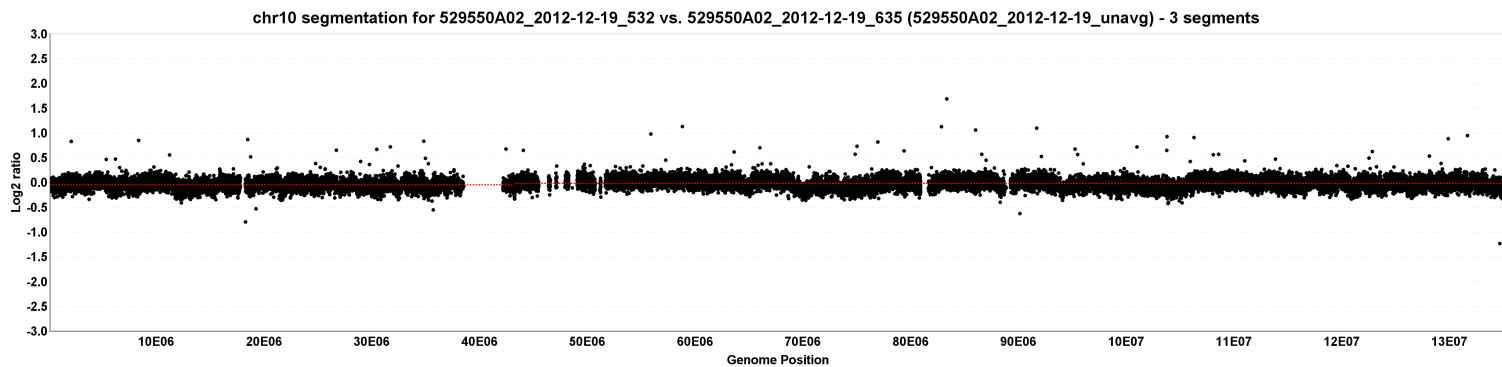

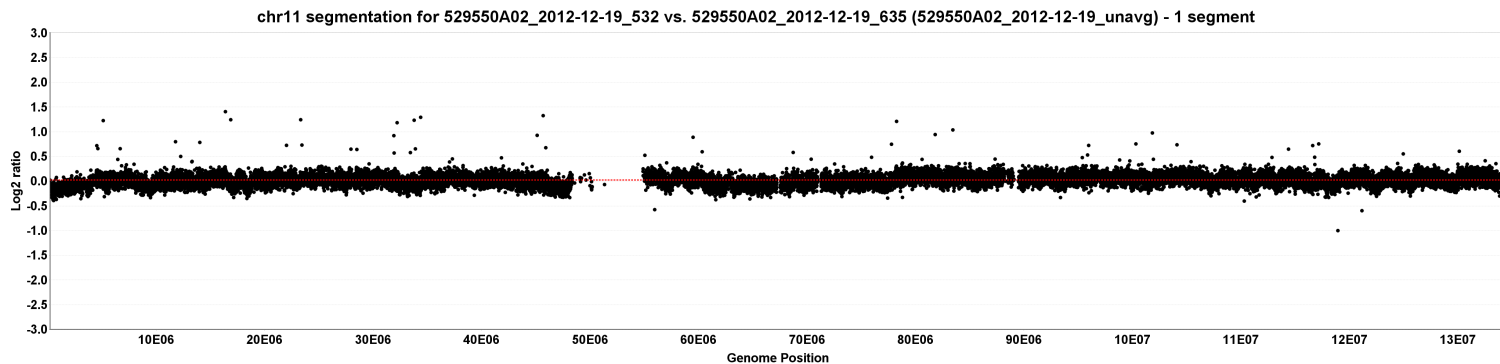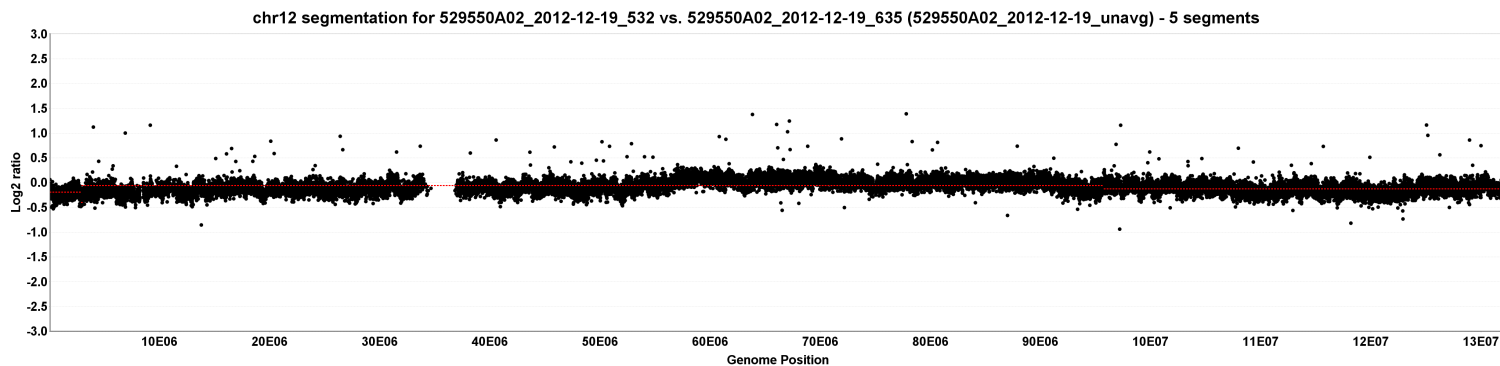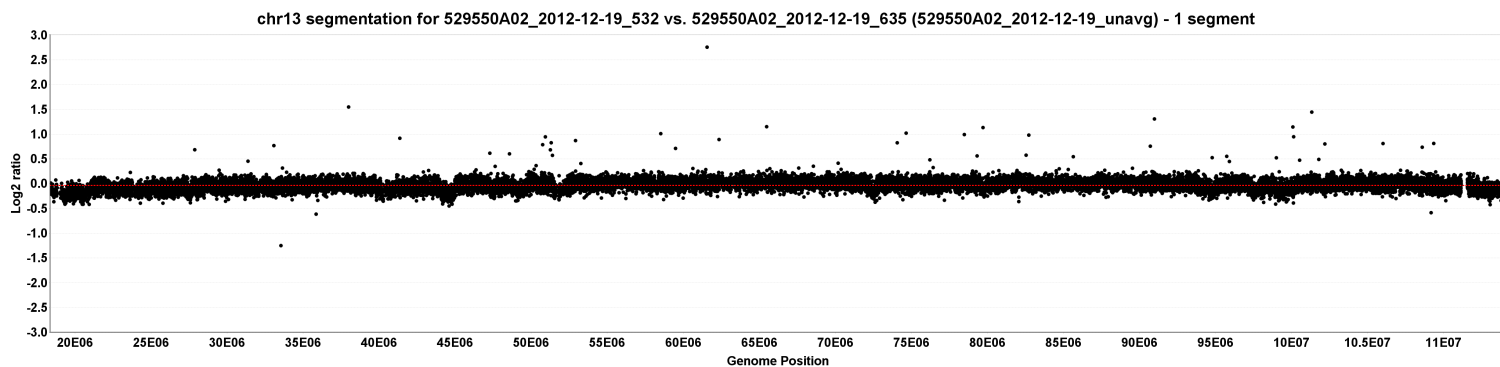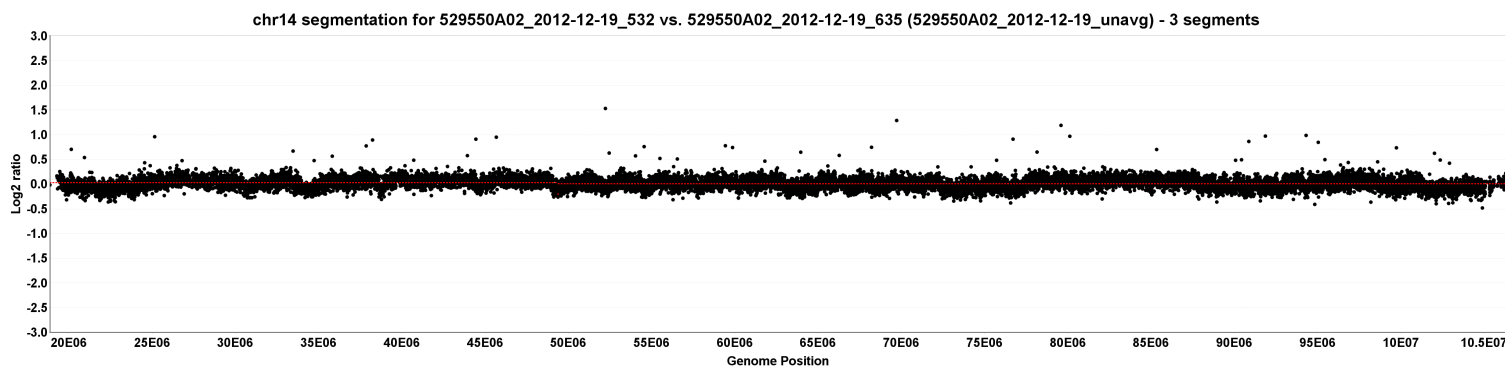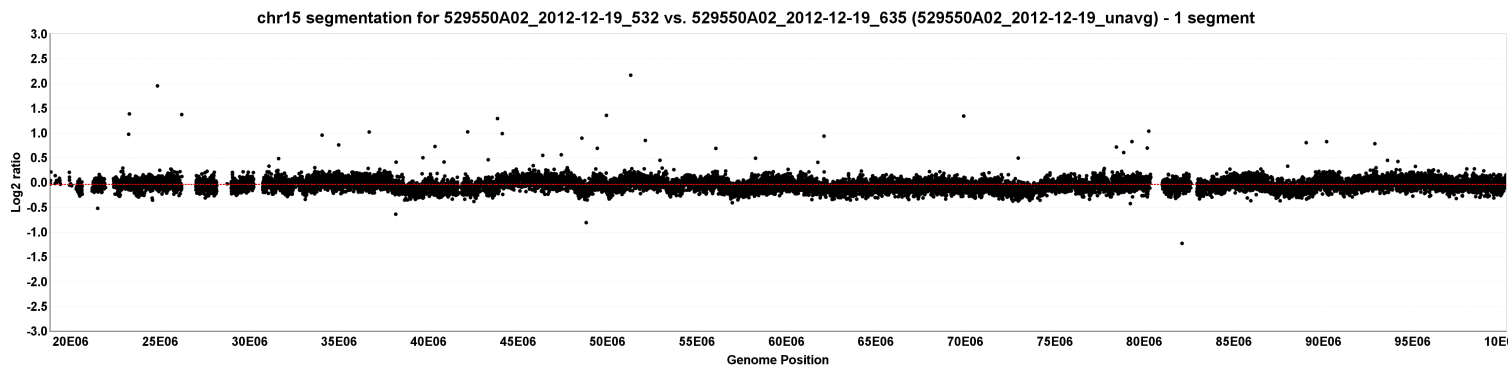

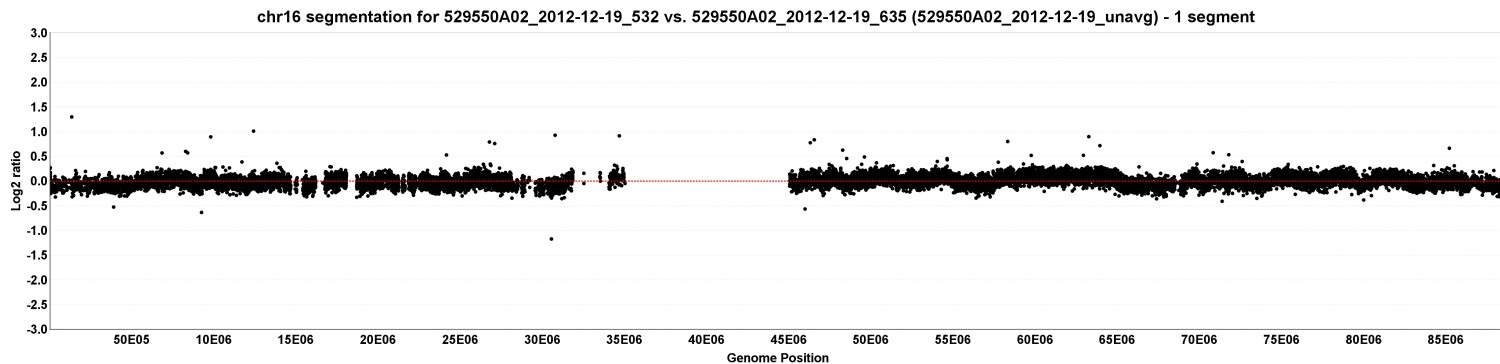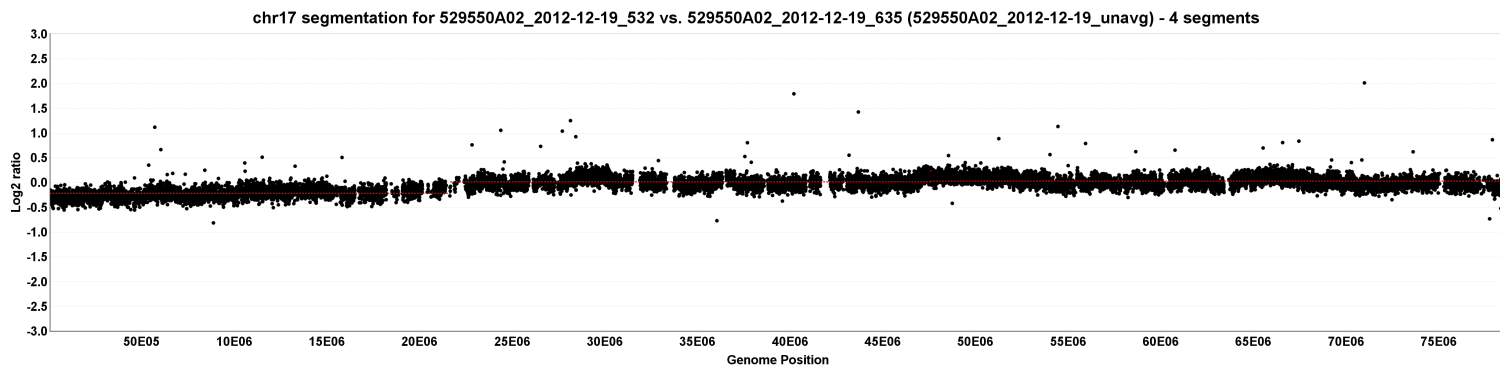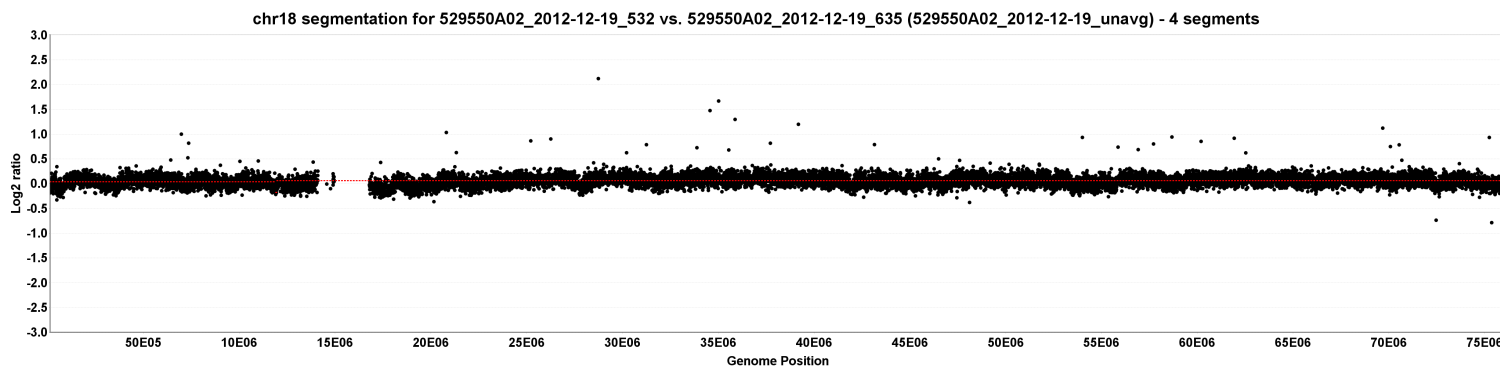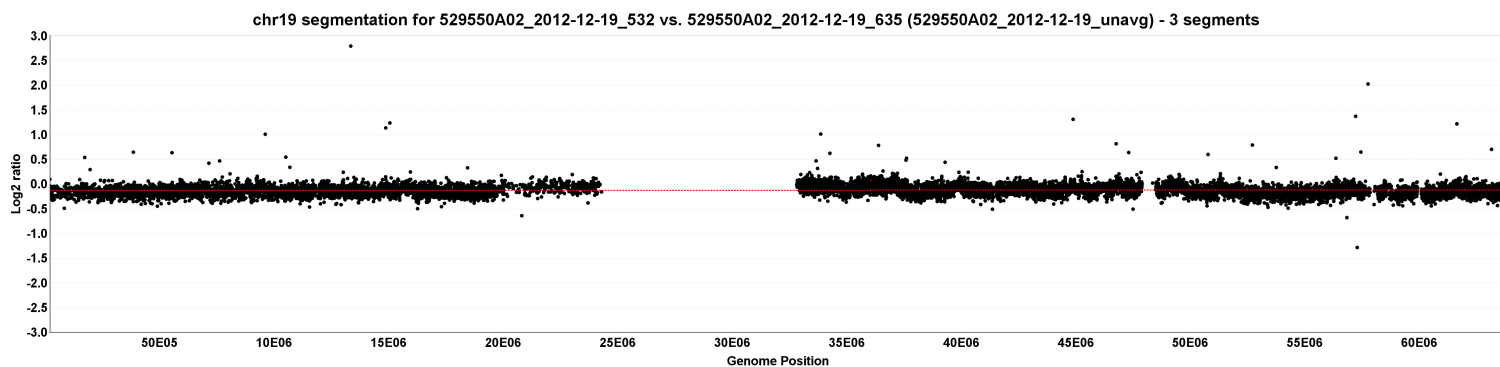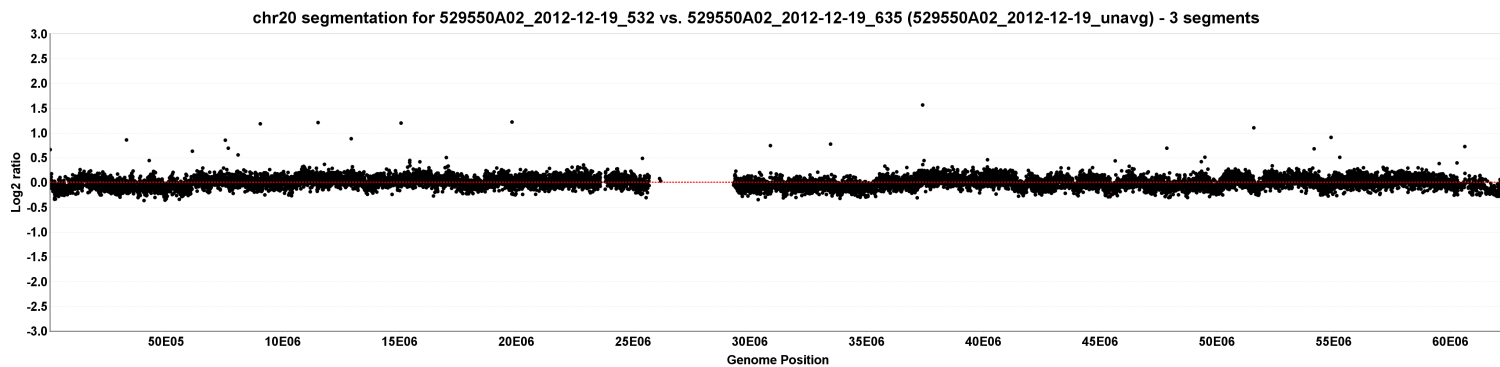

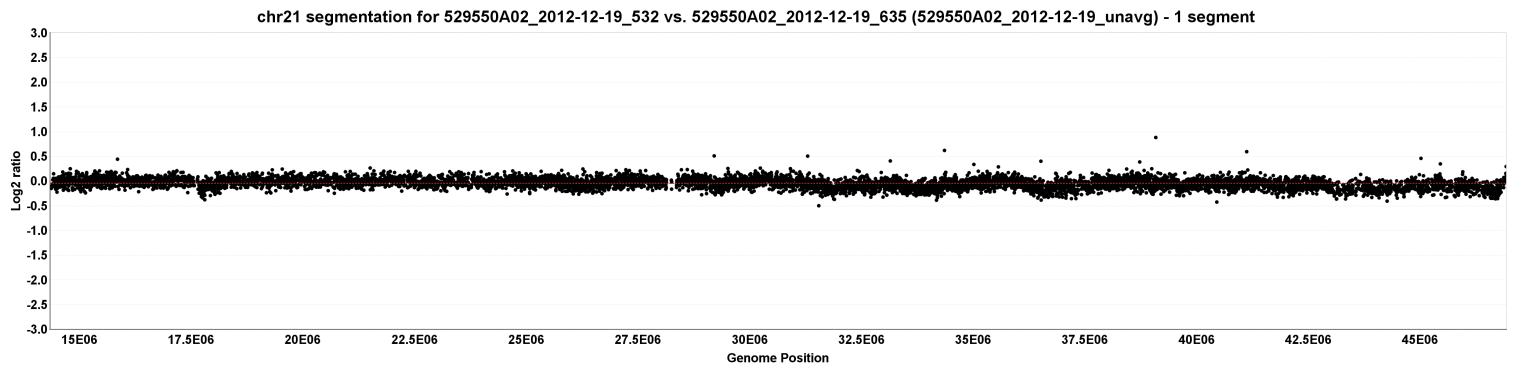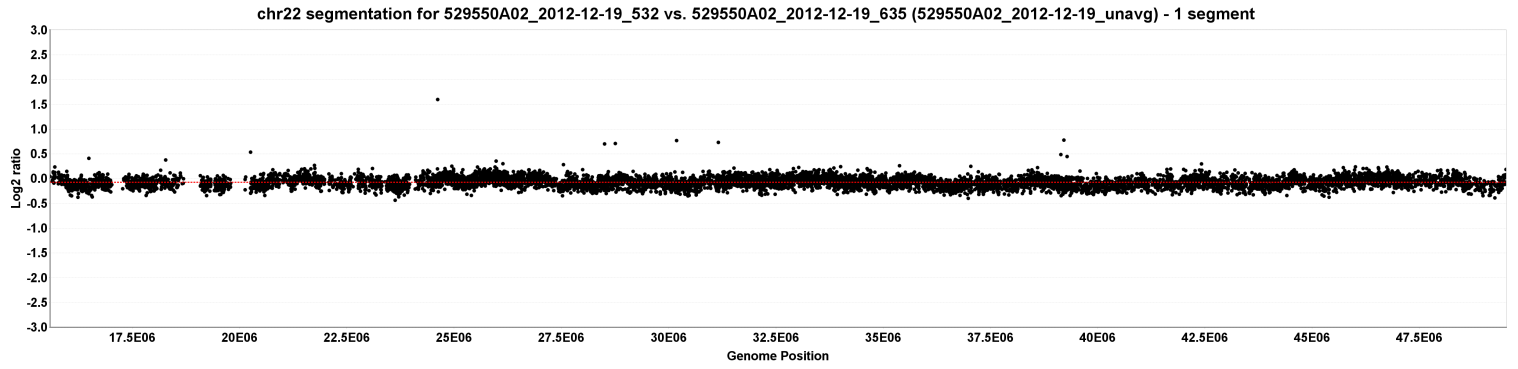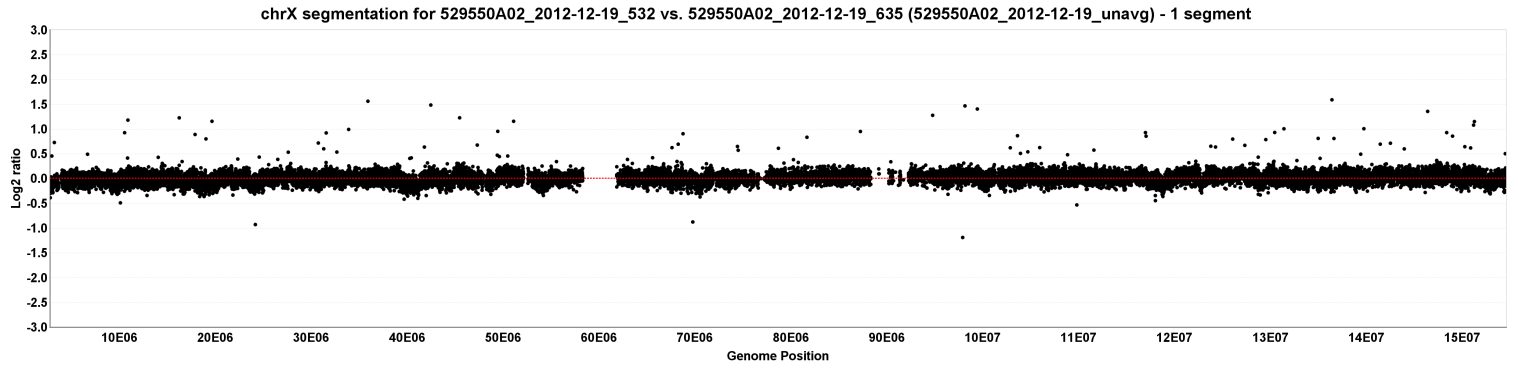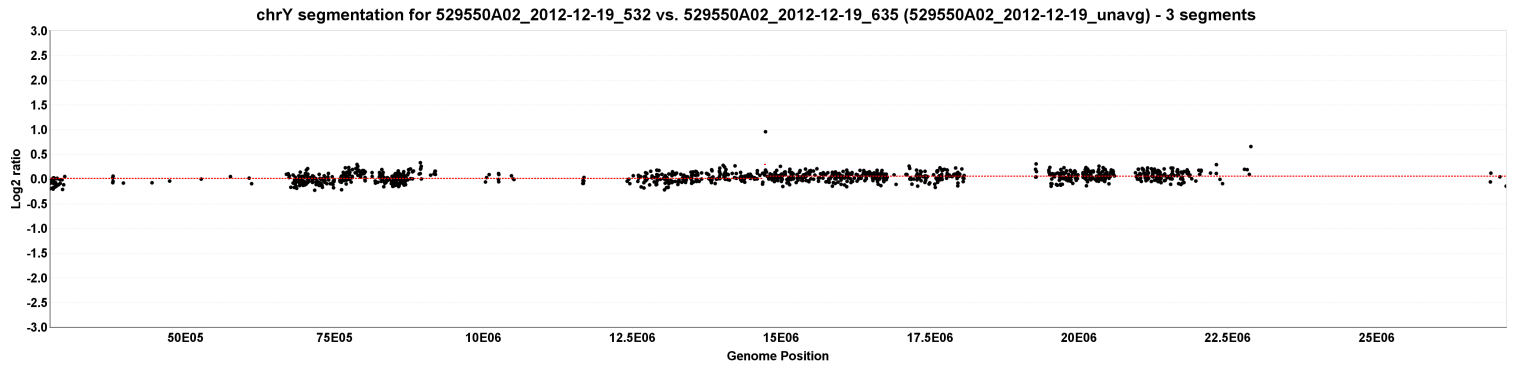

Supplement: S2 File — (PDF) [file pone.0169098.s002.pdf]

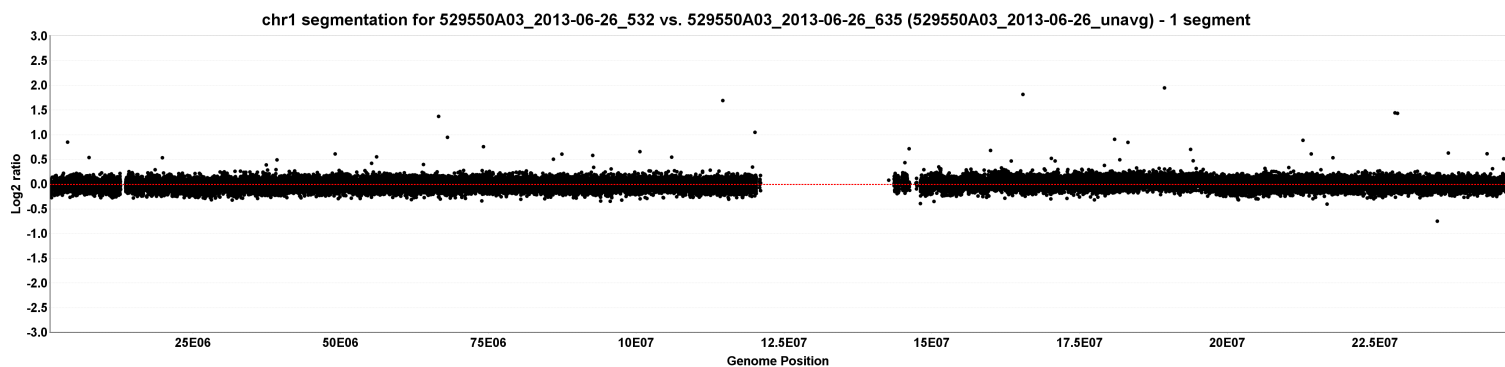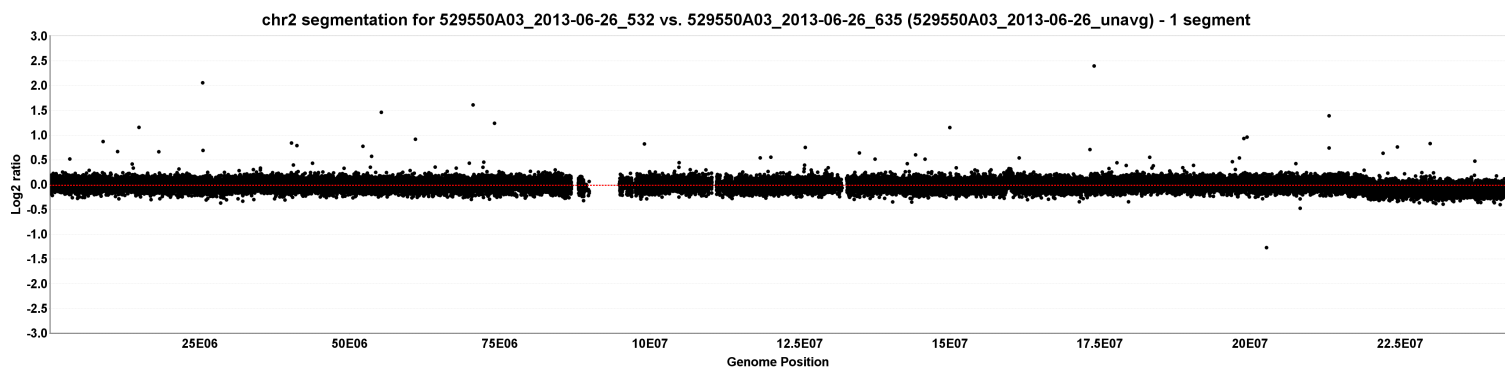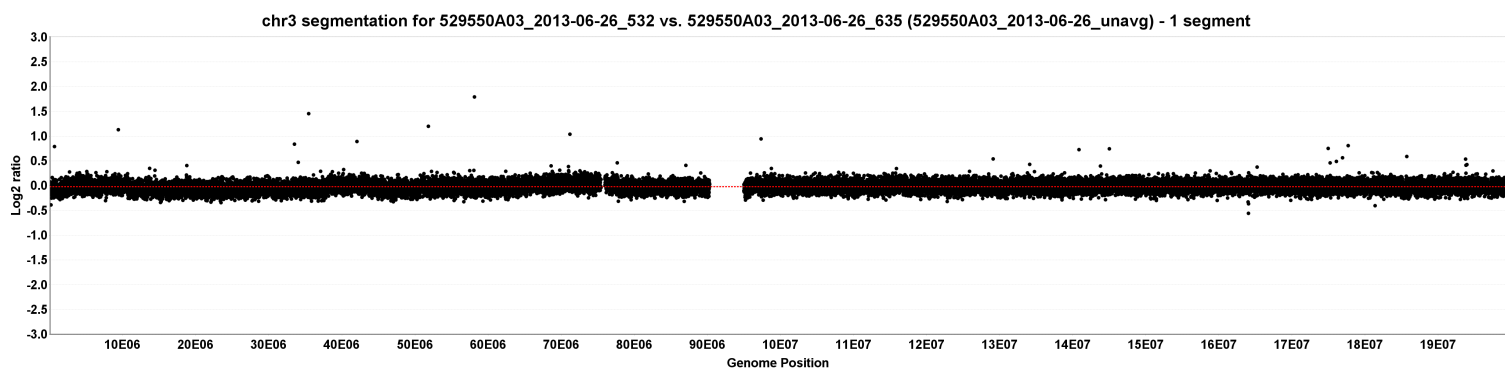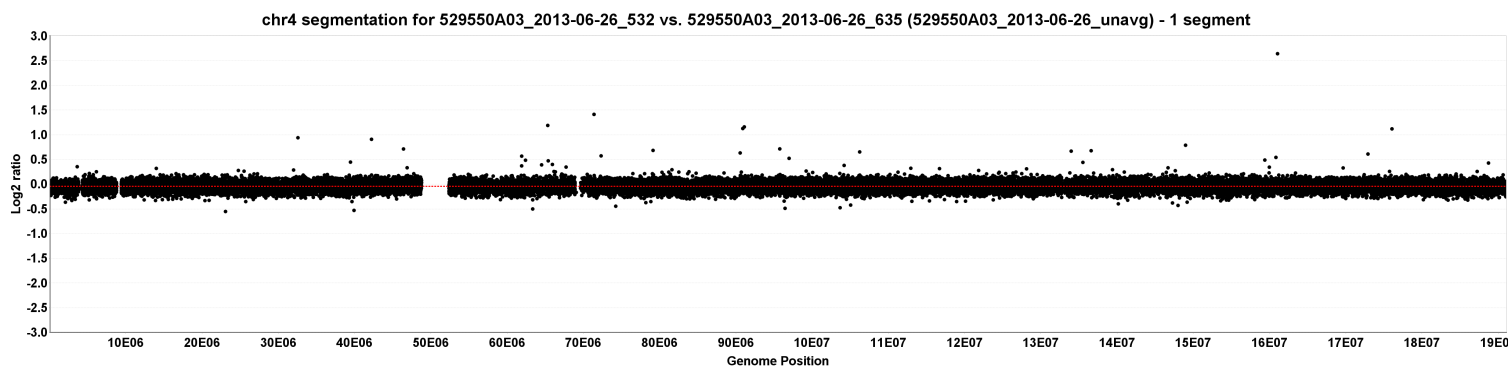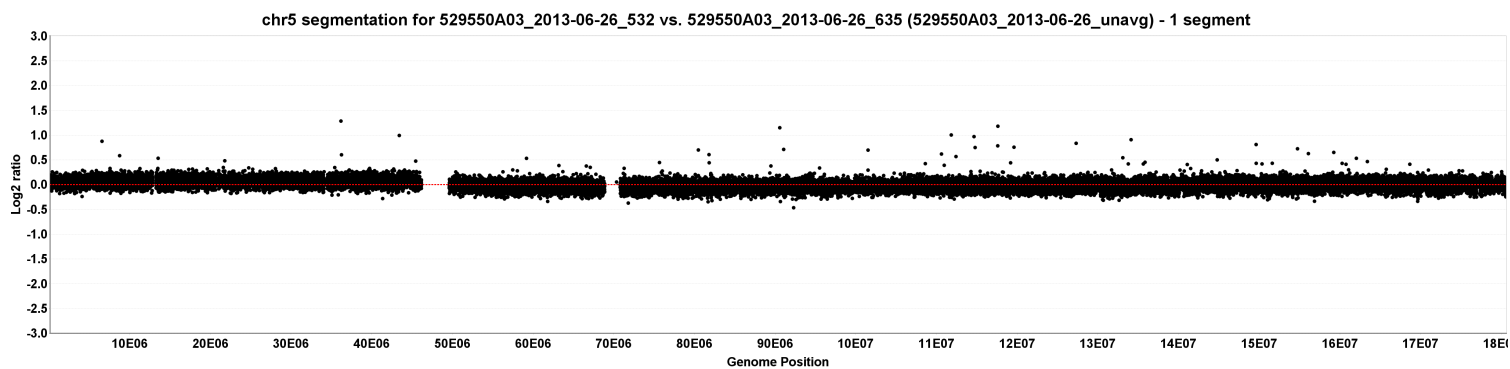

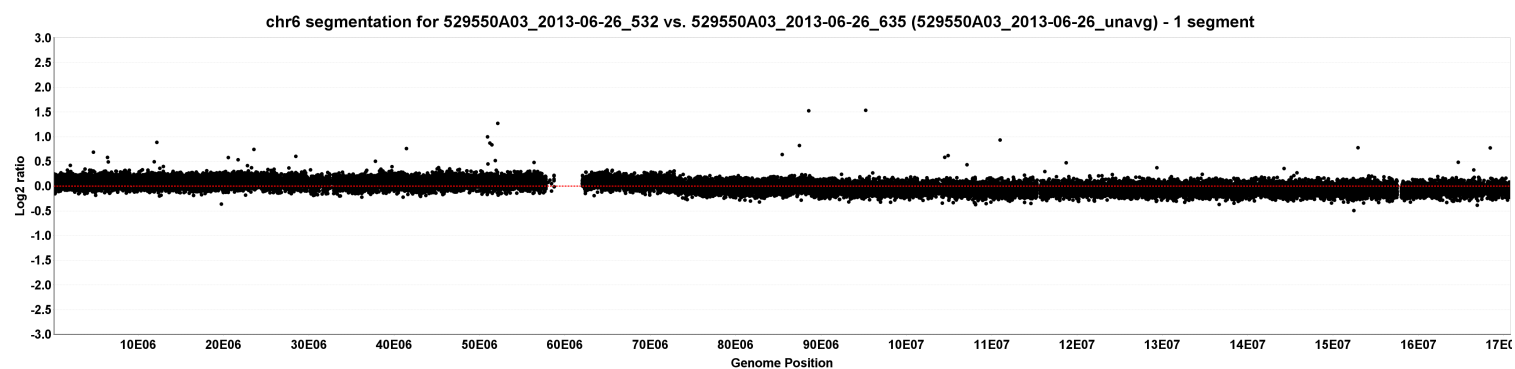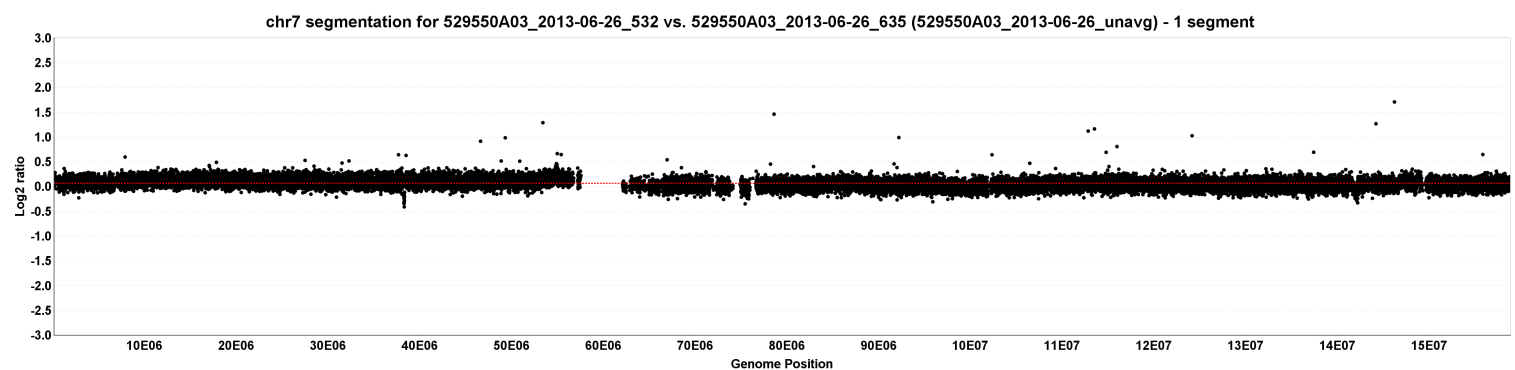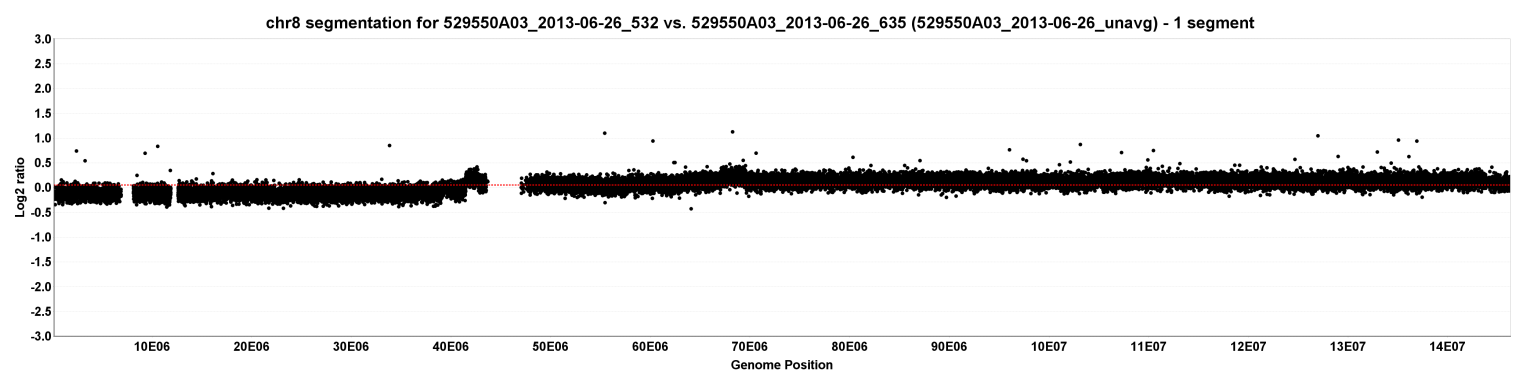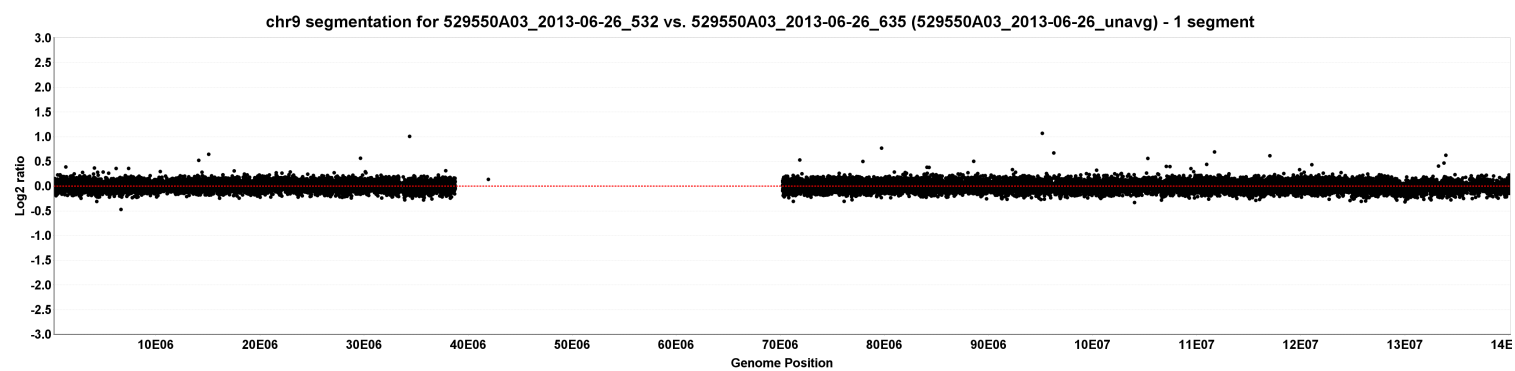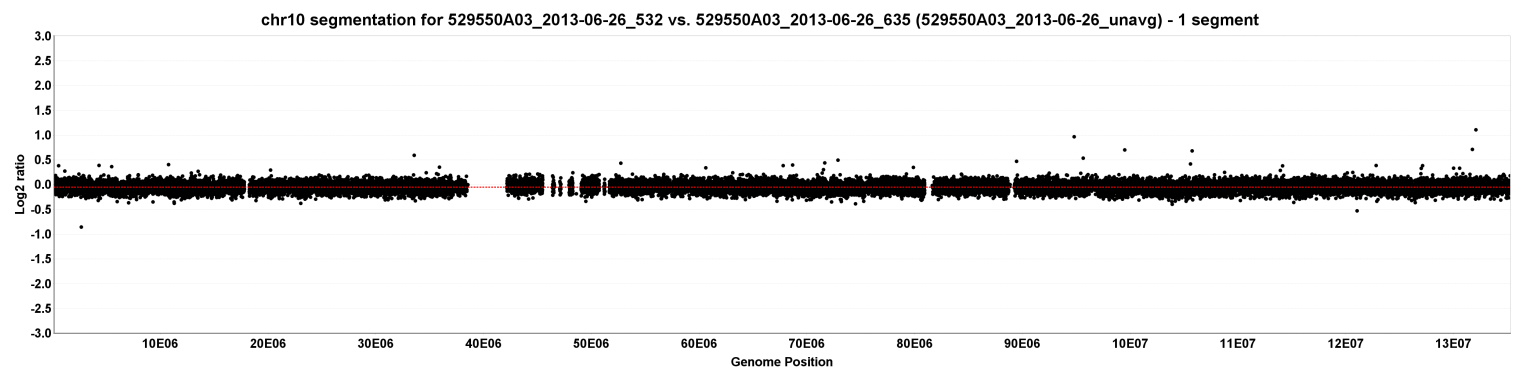

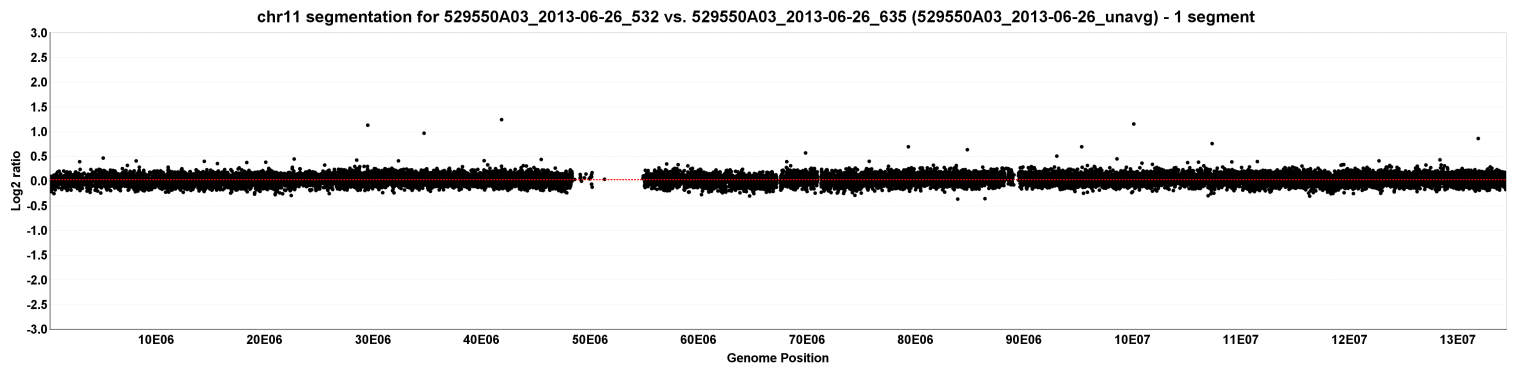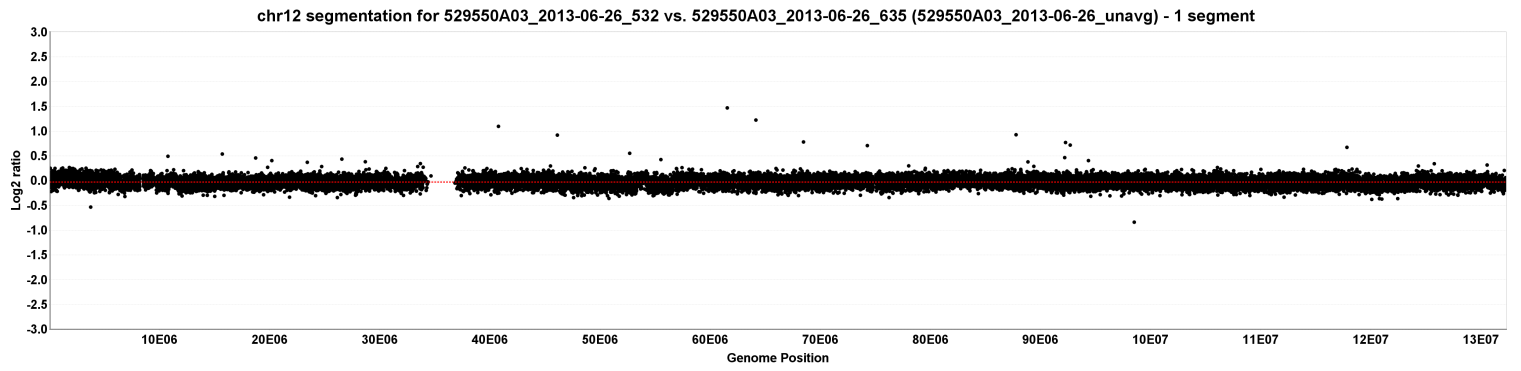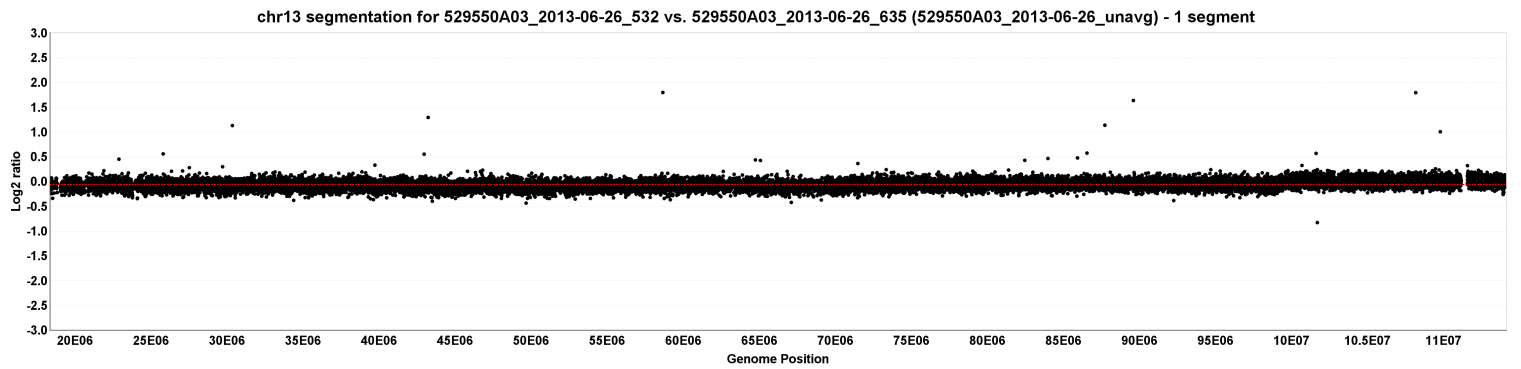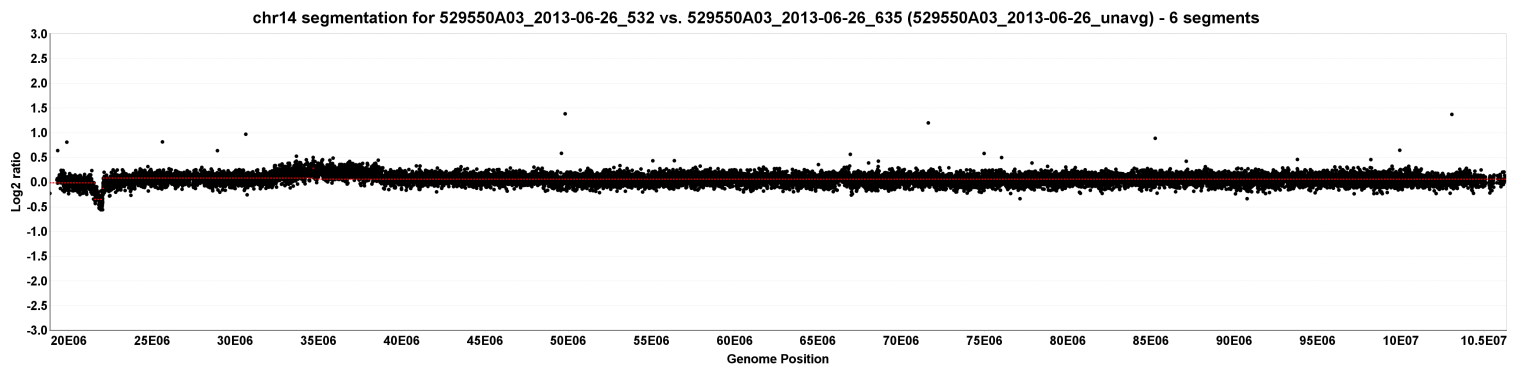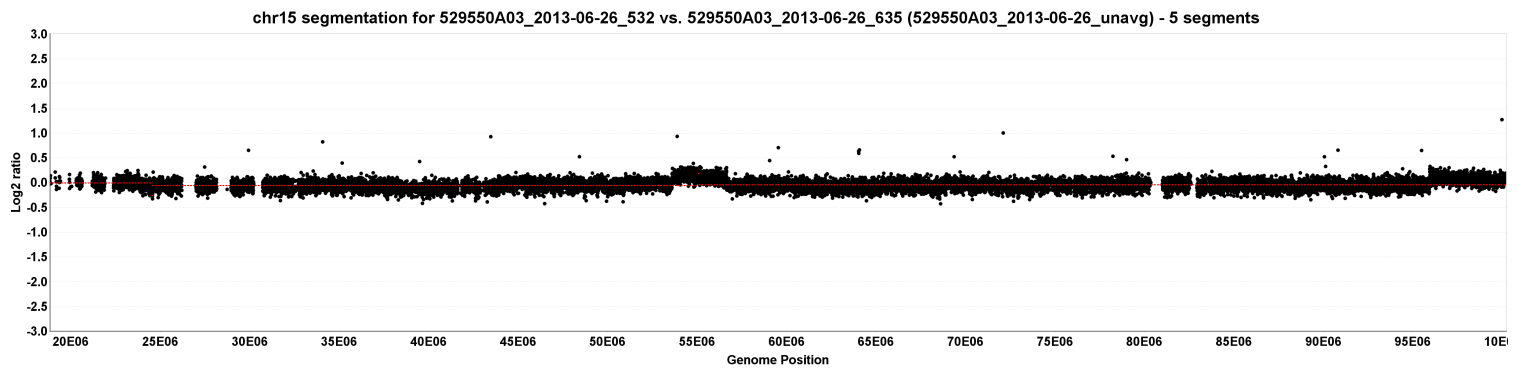

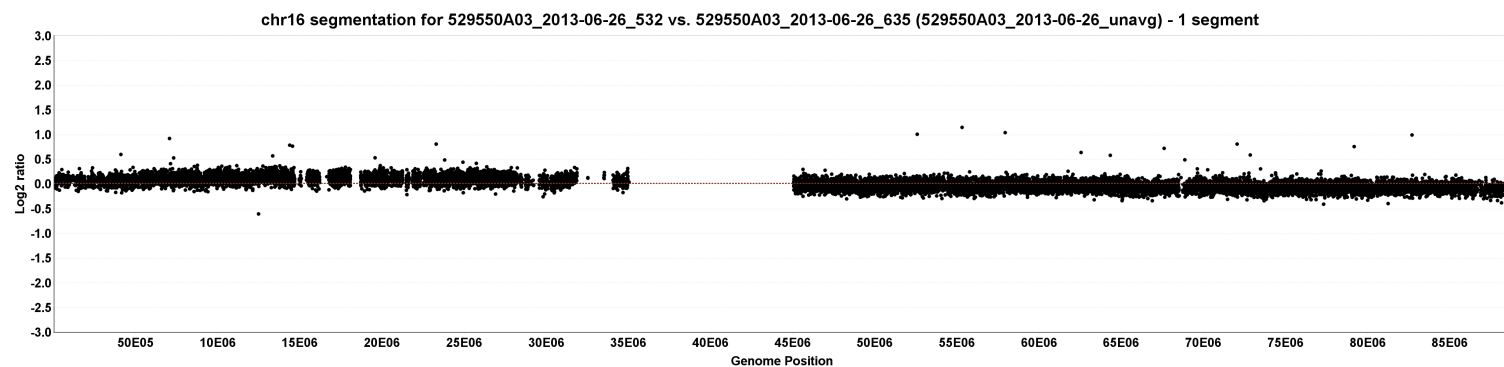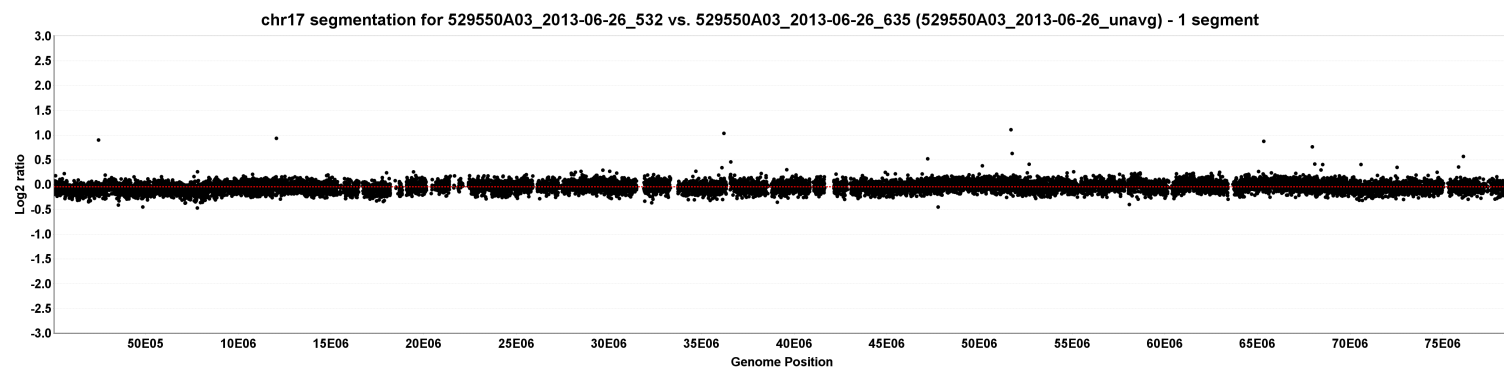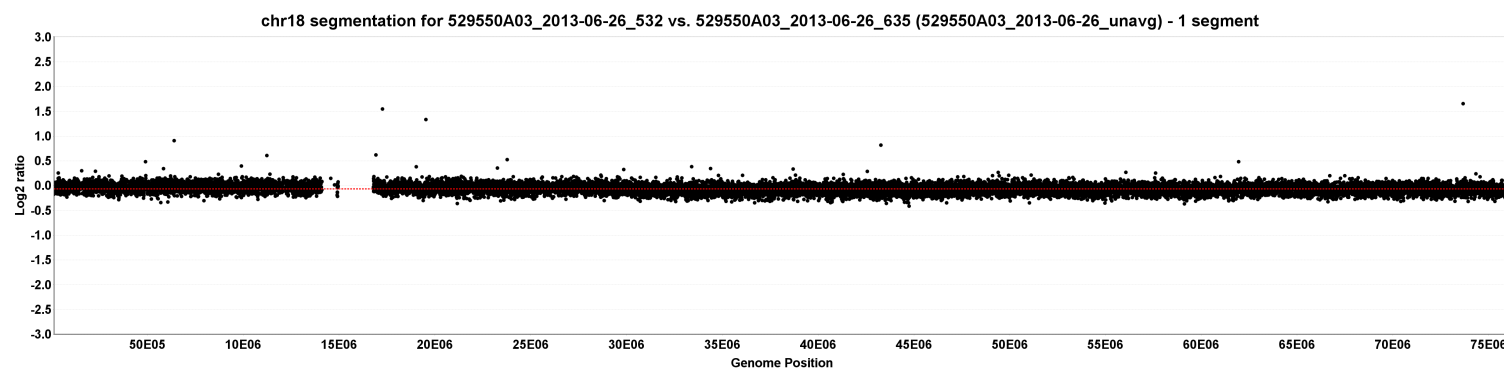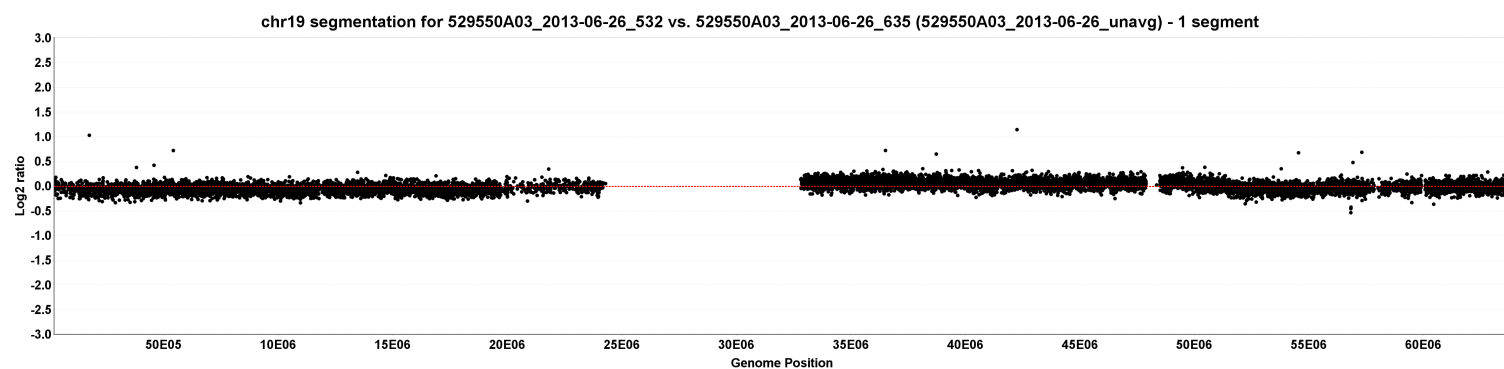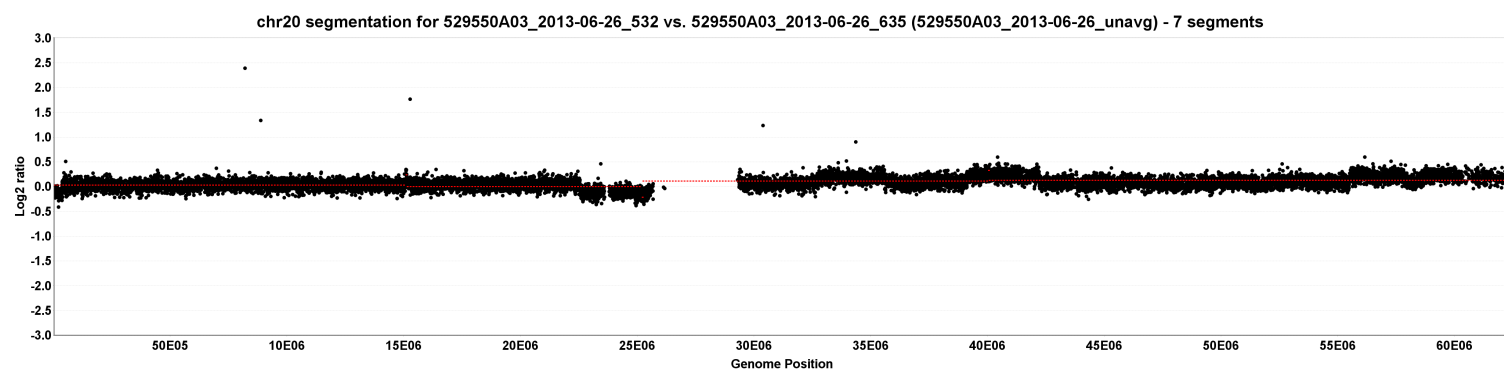

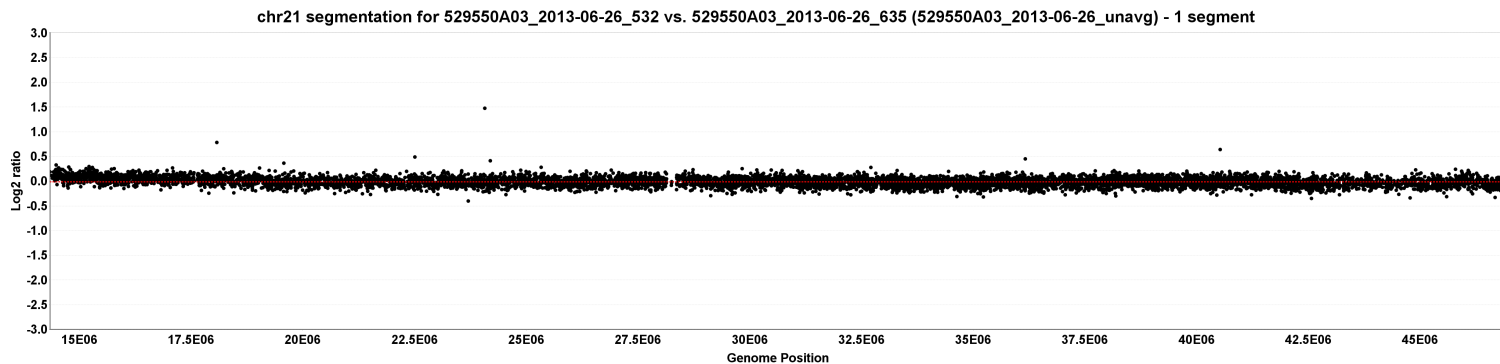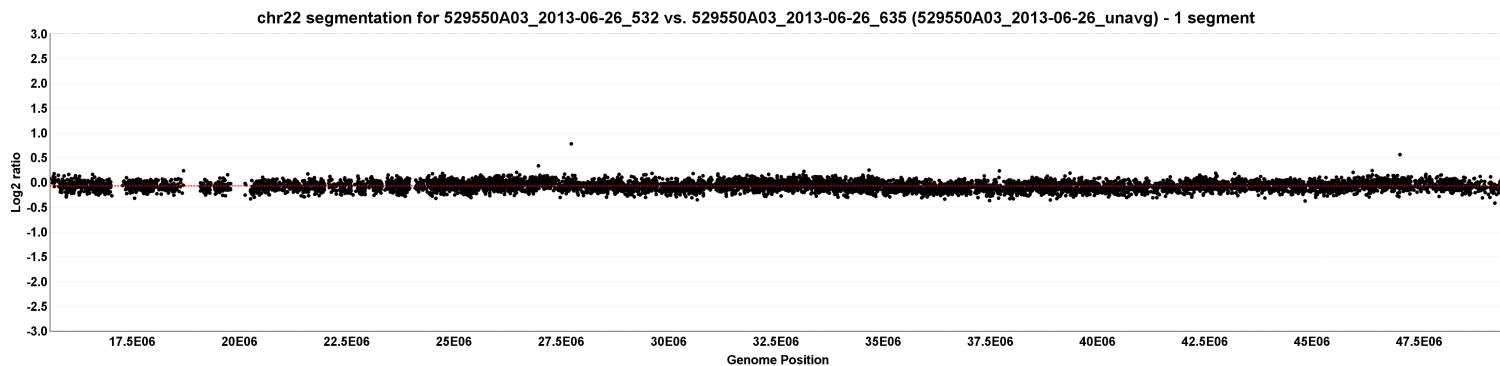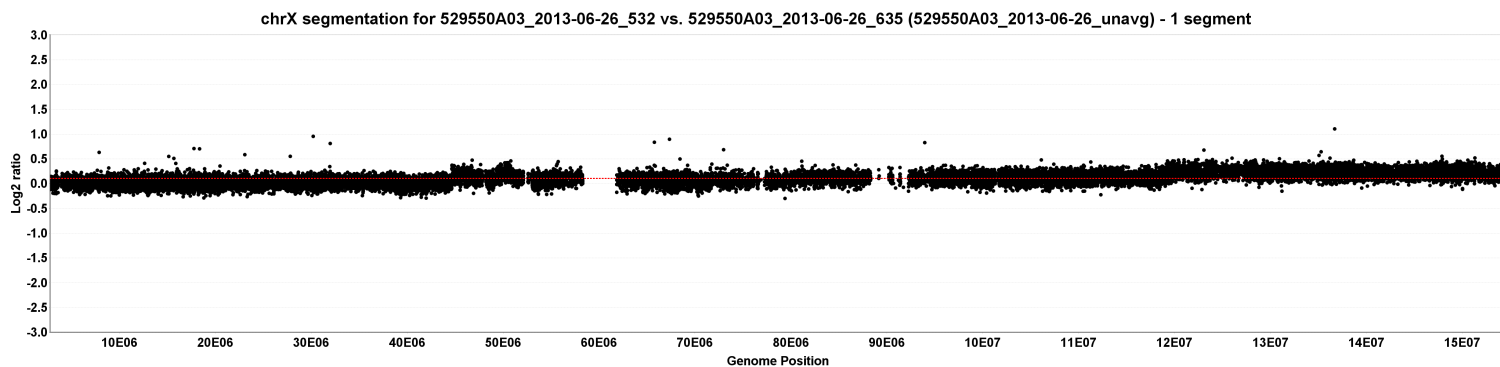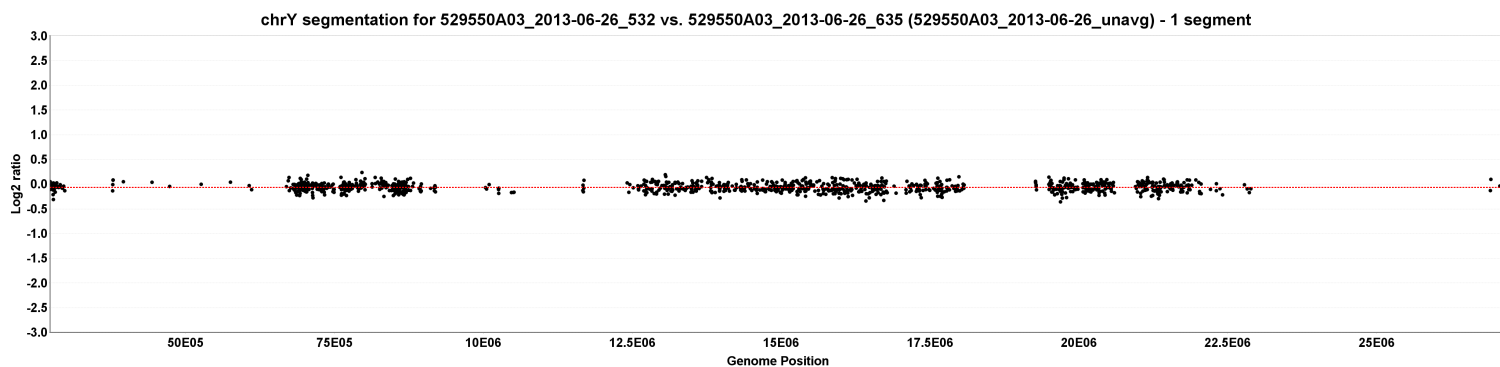

Supplement: S3 File — (PDF) [file pone.0169098.s003.pdf]

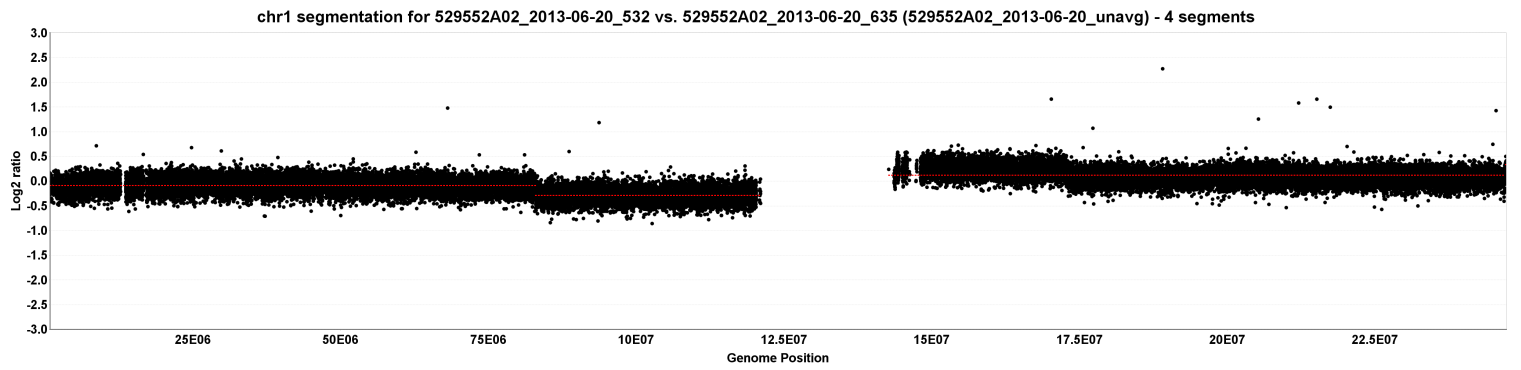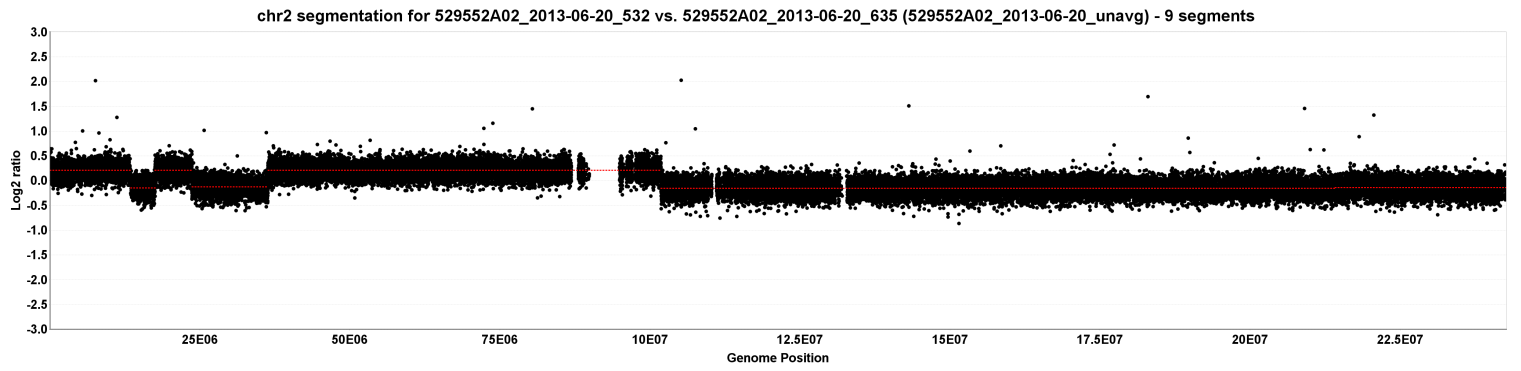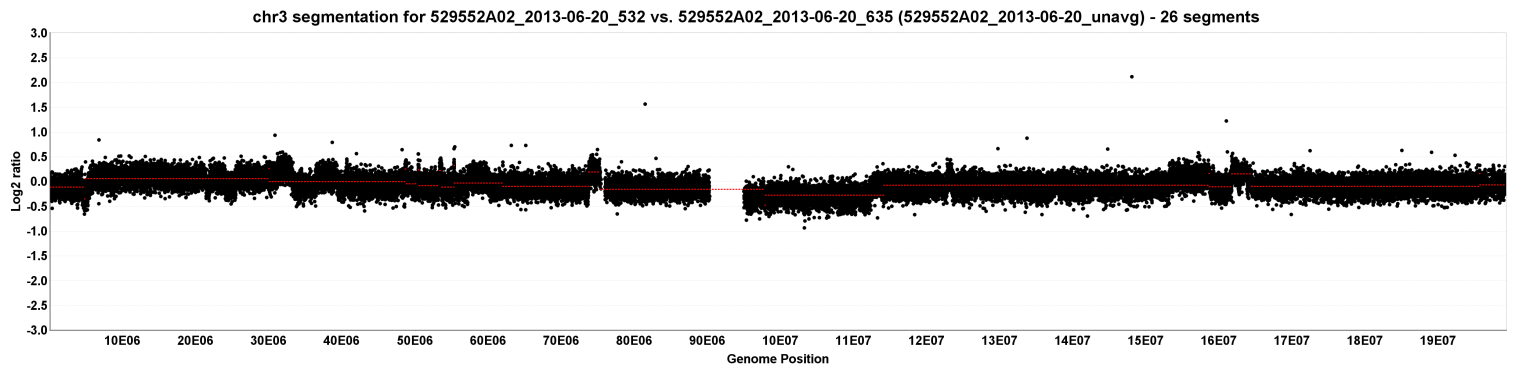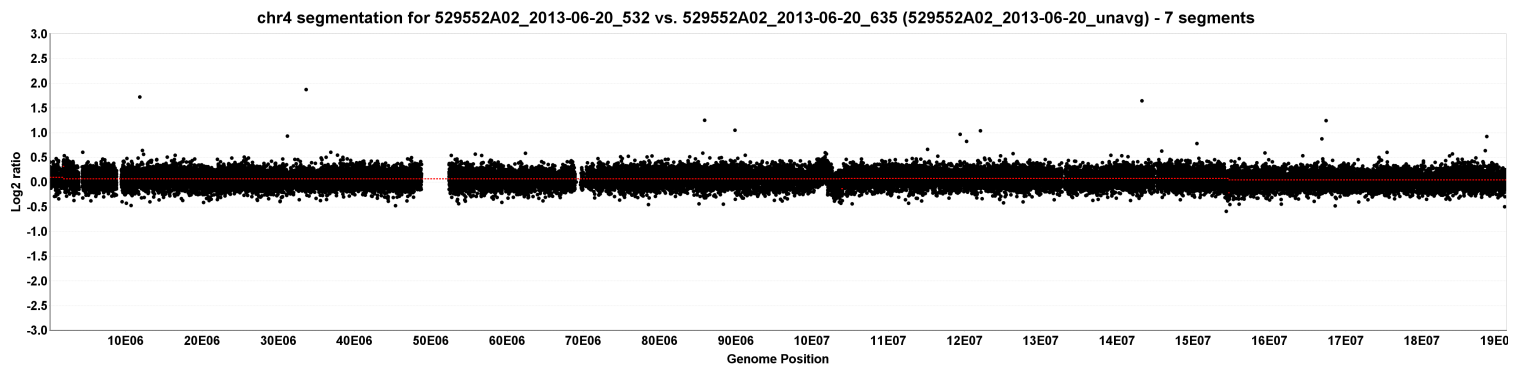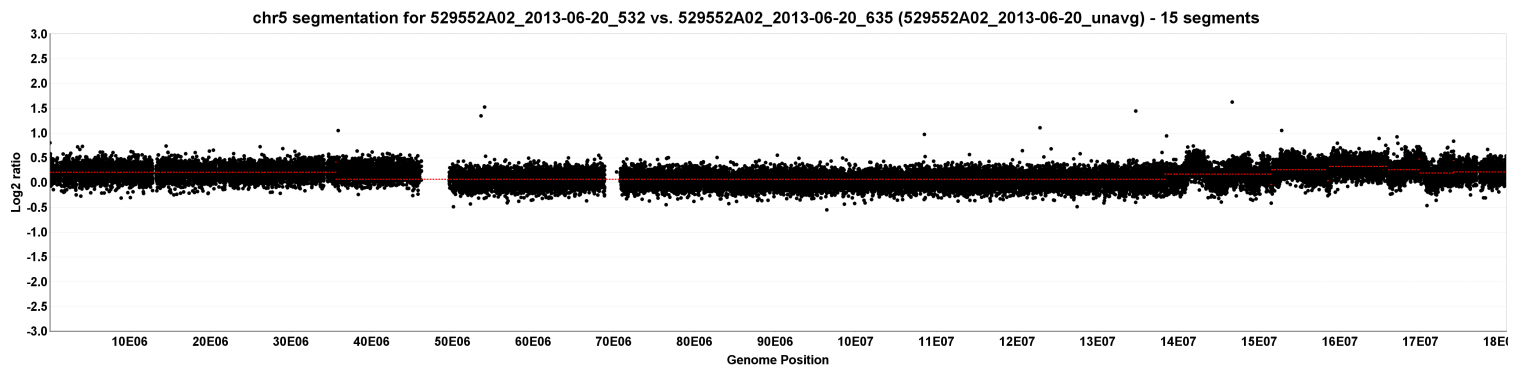

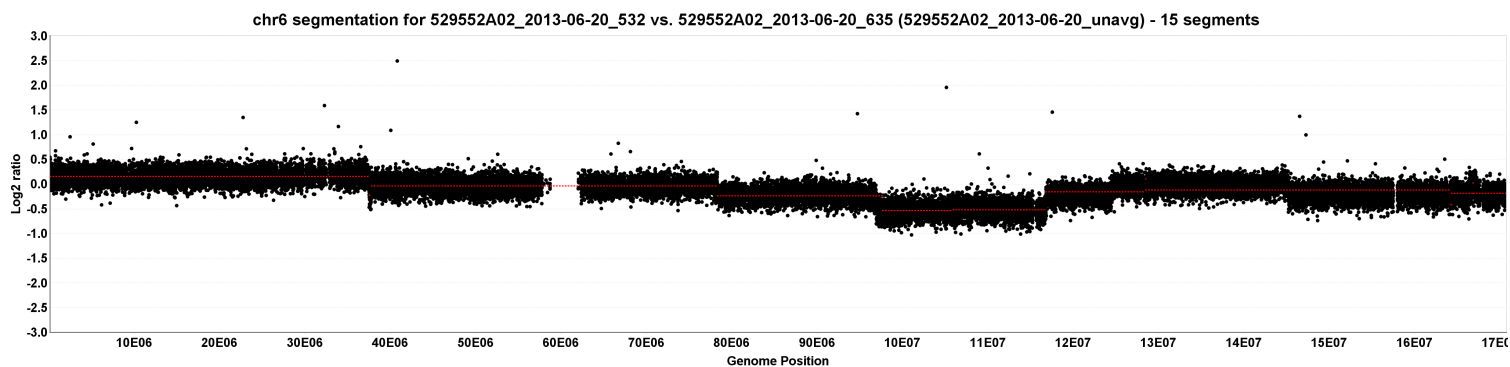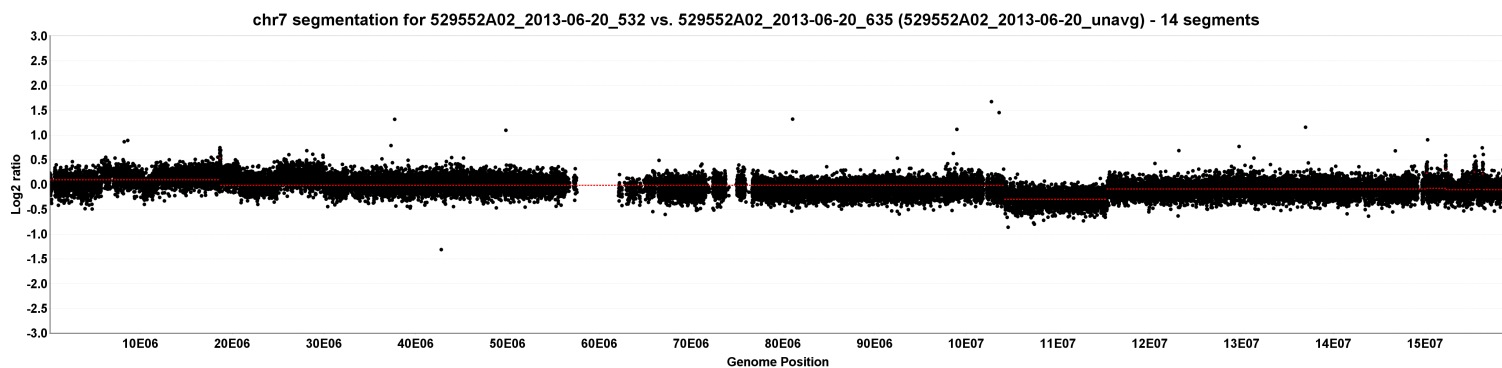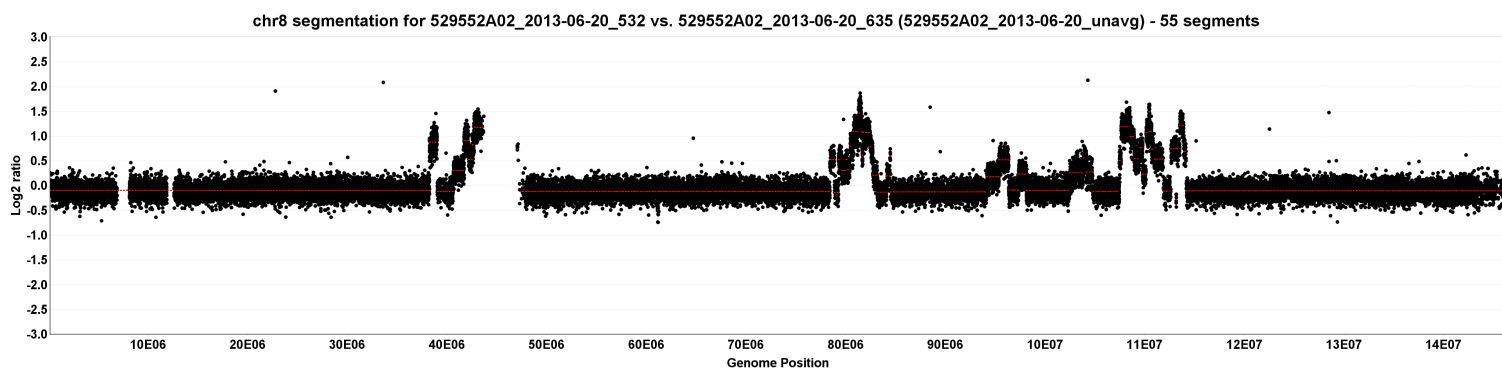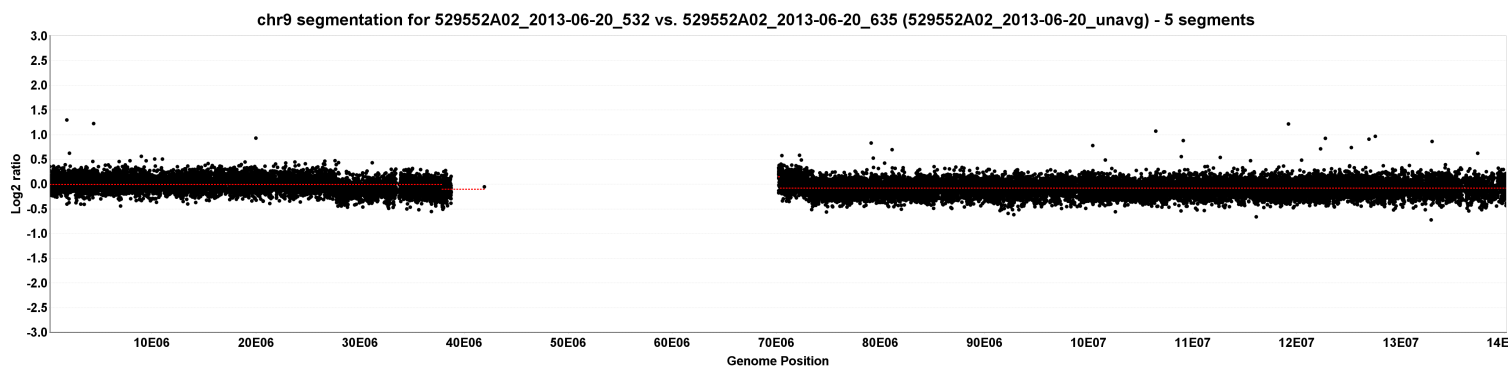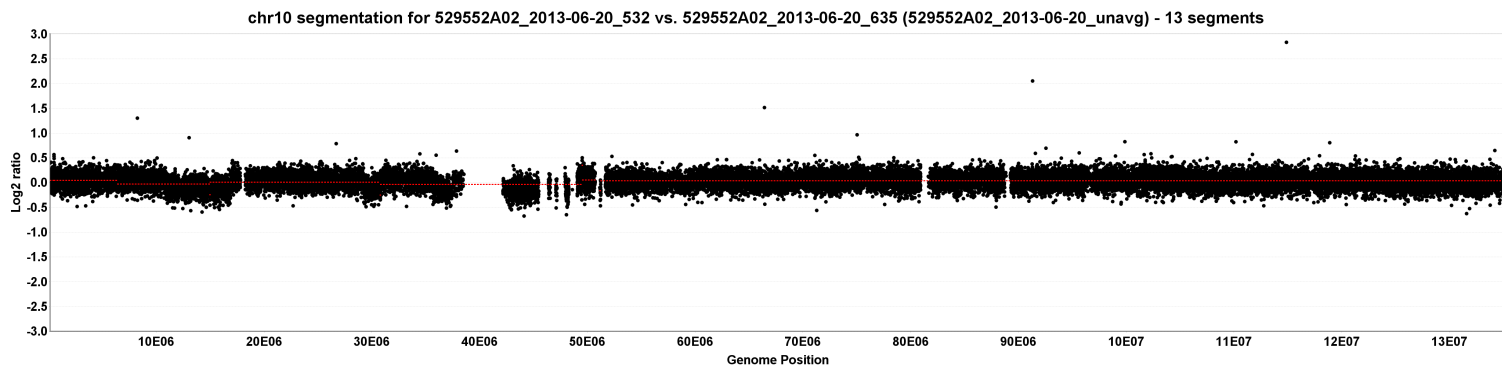

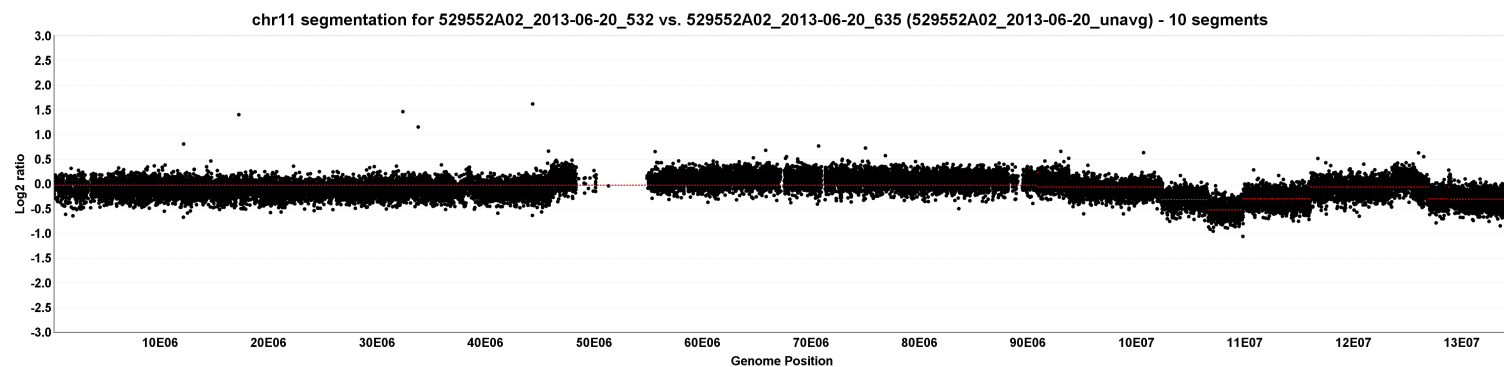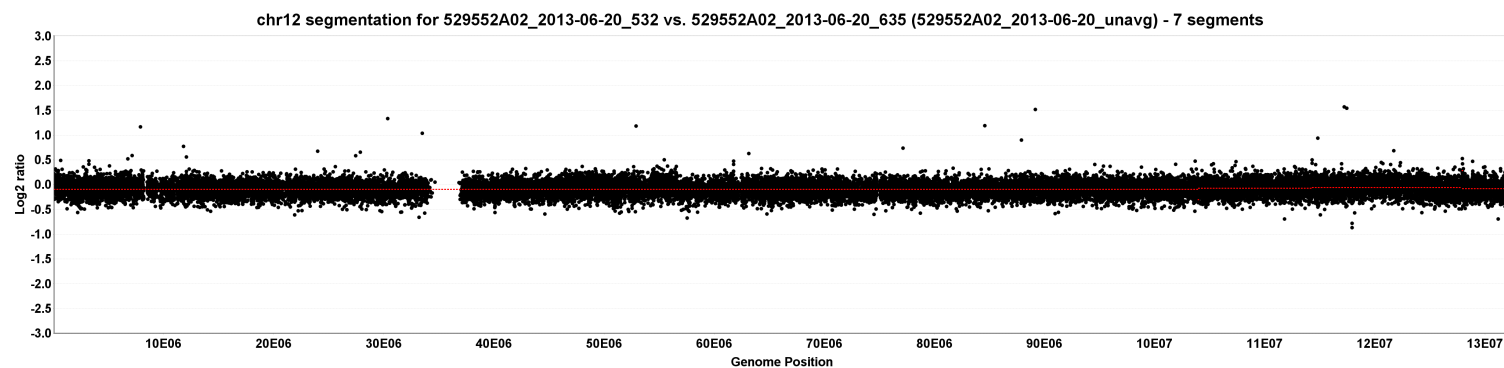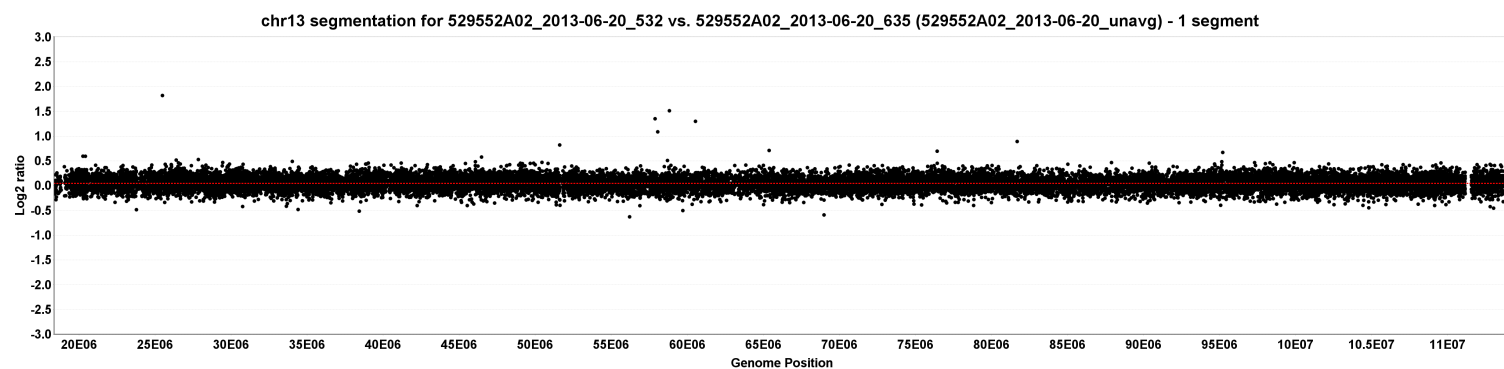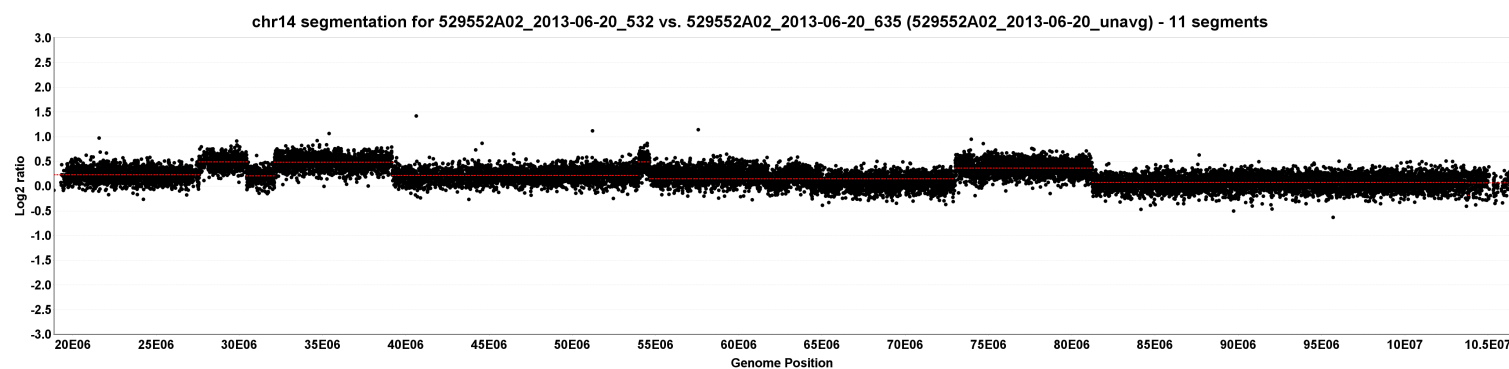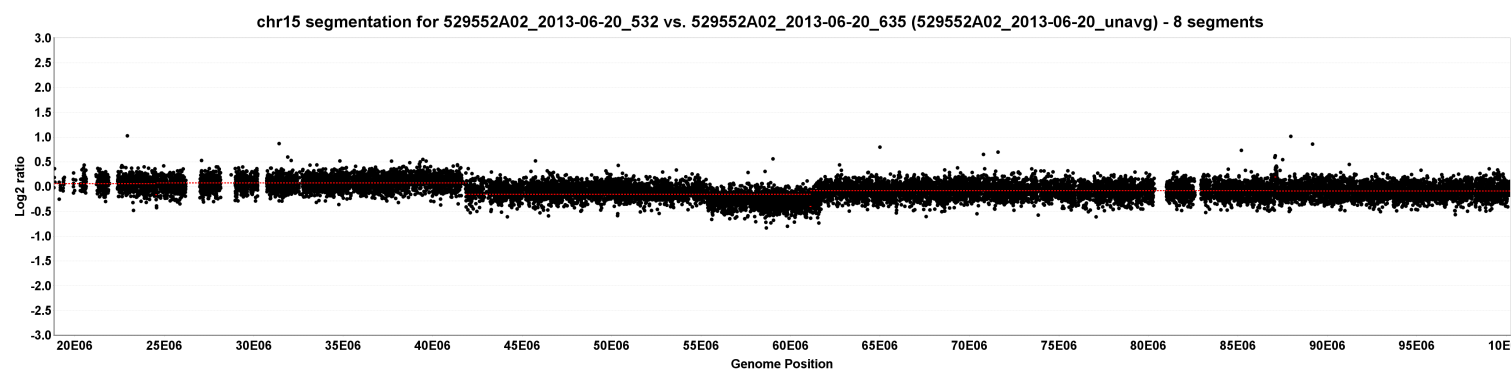

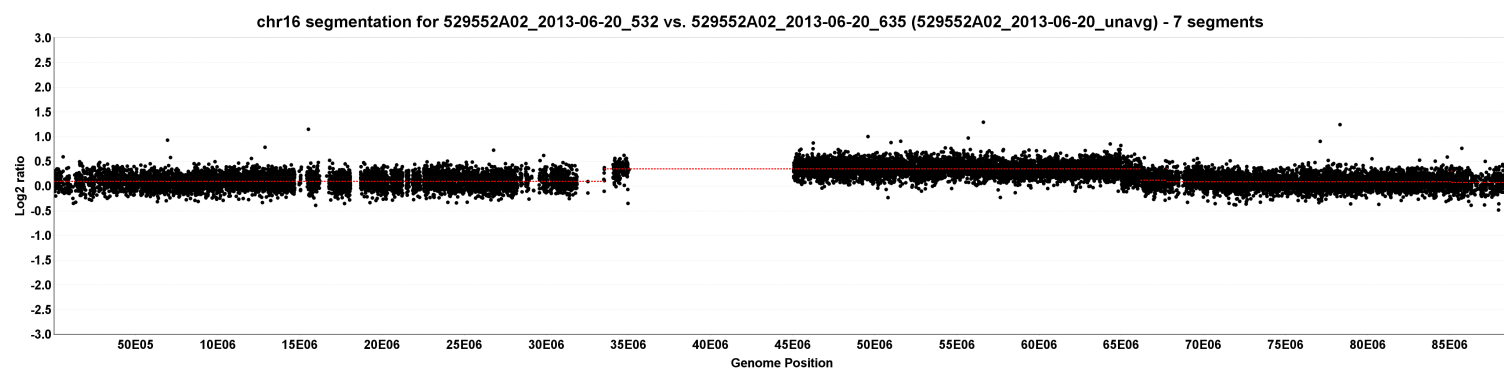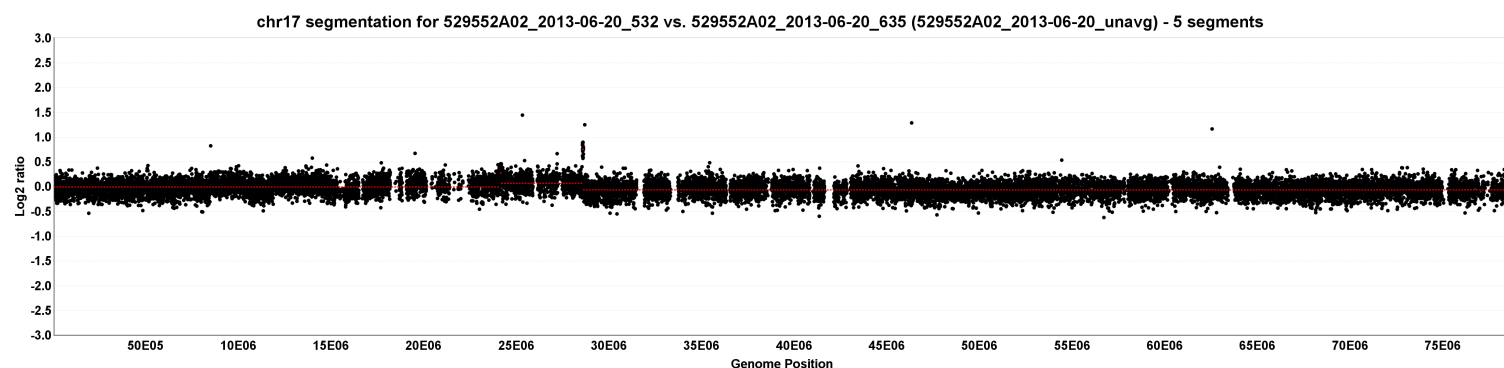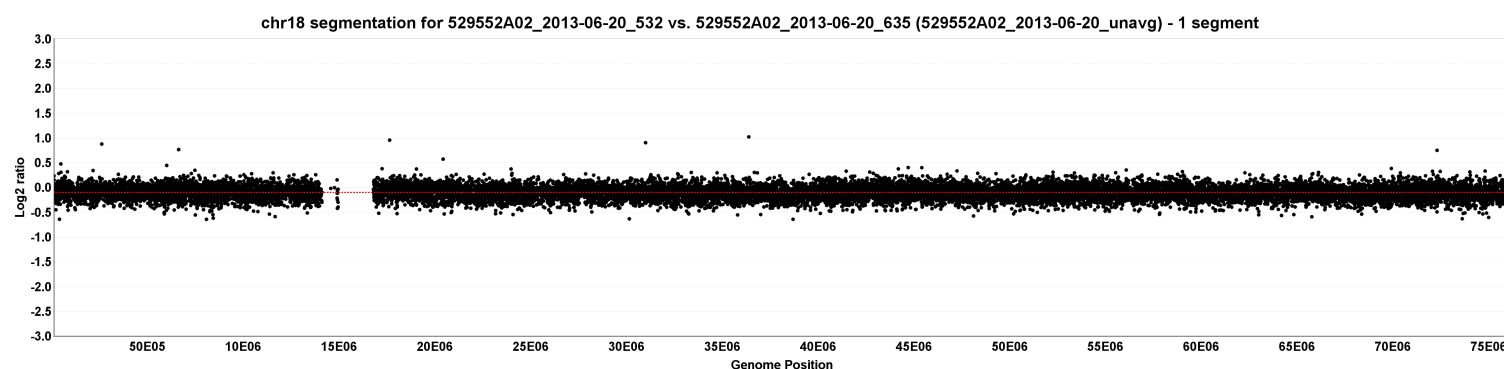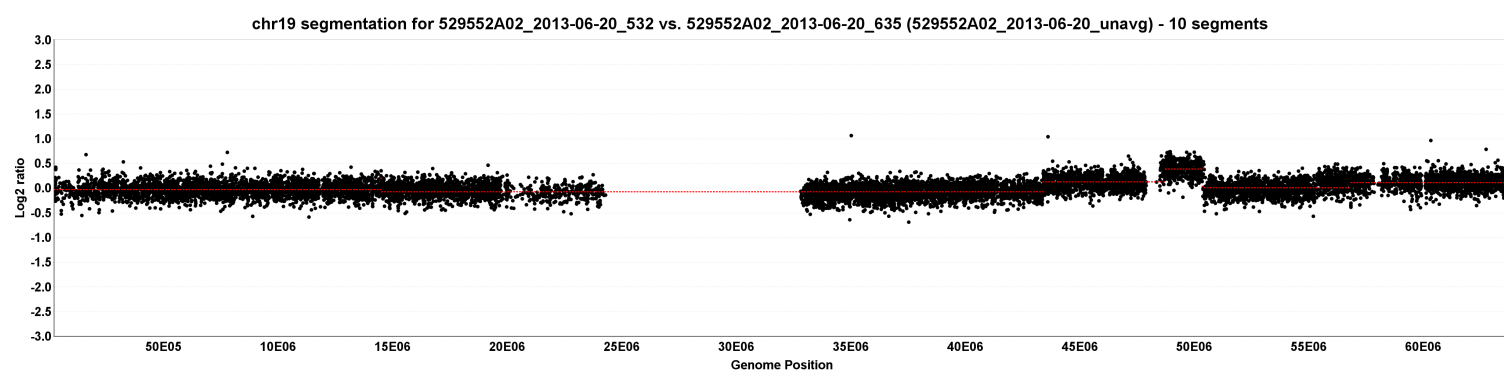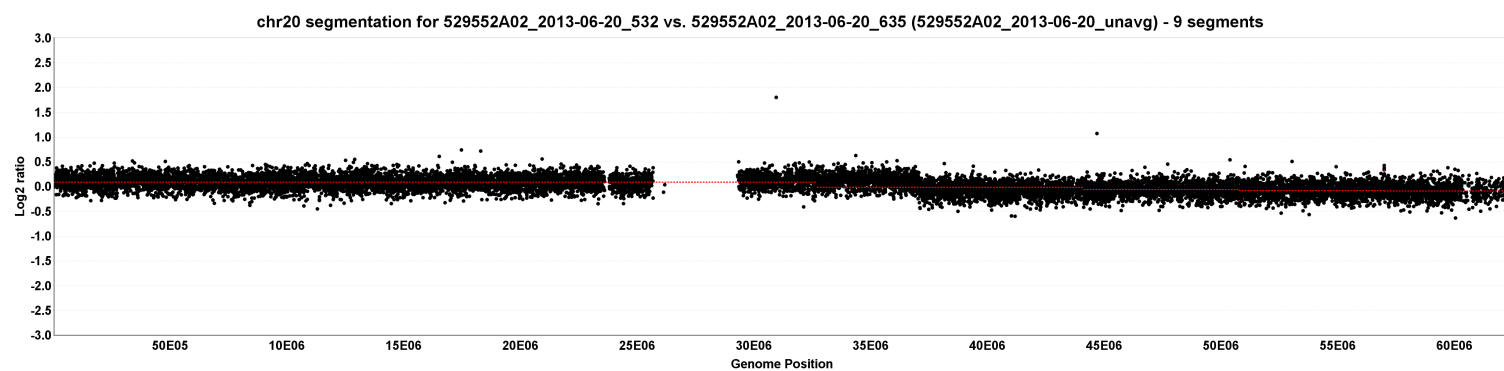

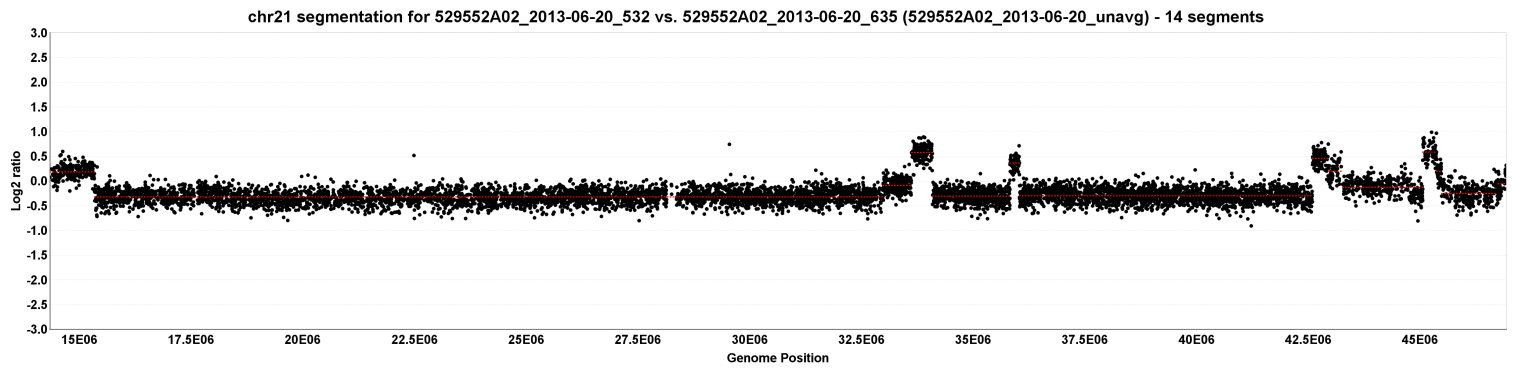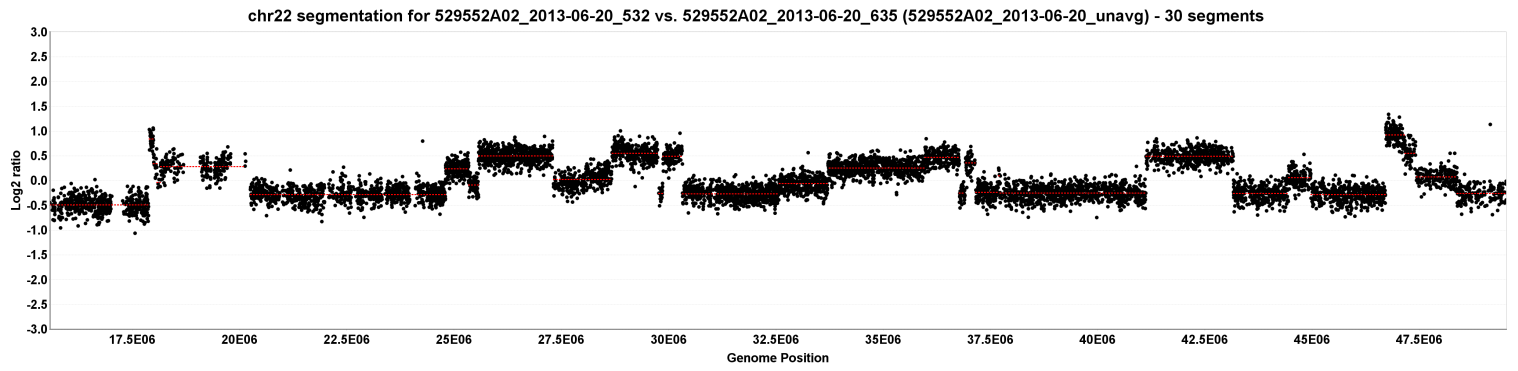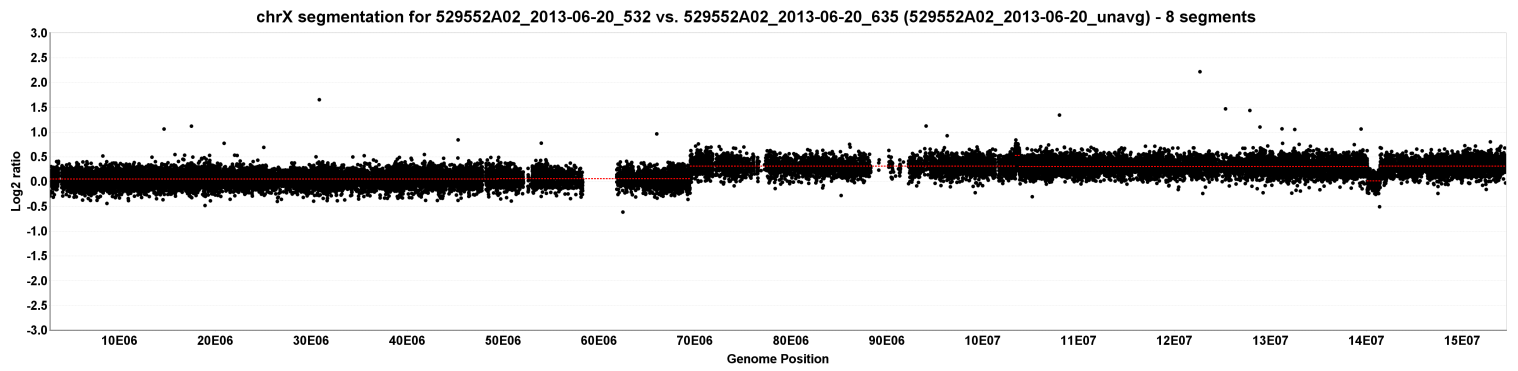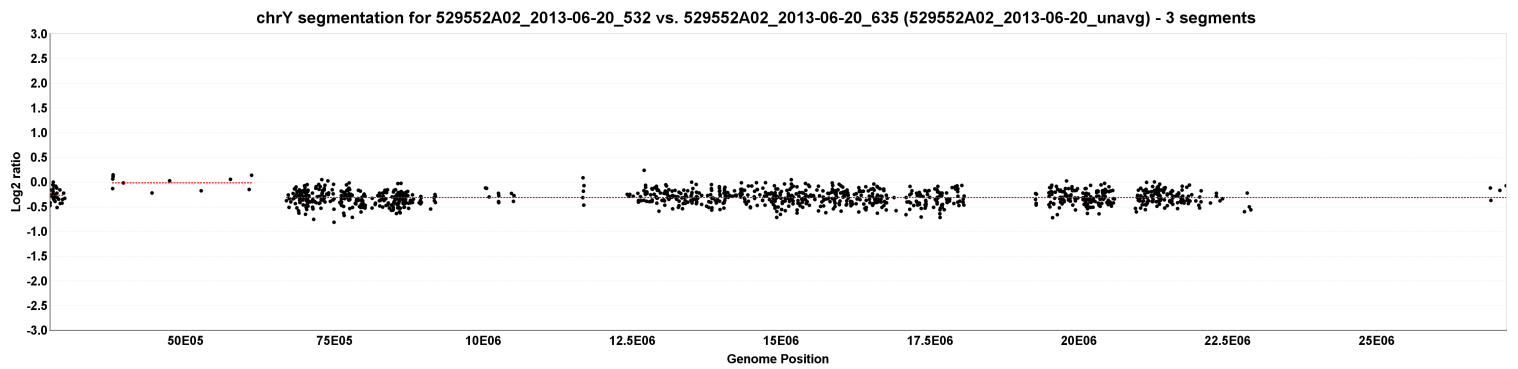

Supplement: S4 File — (PDF) [file pone.0169098.s004.pdf]

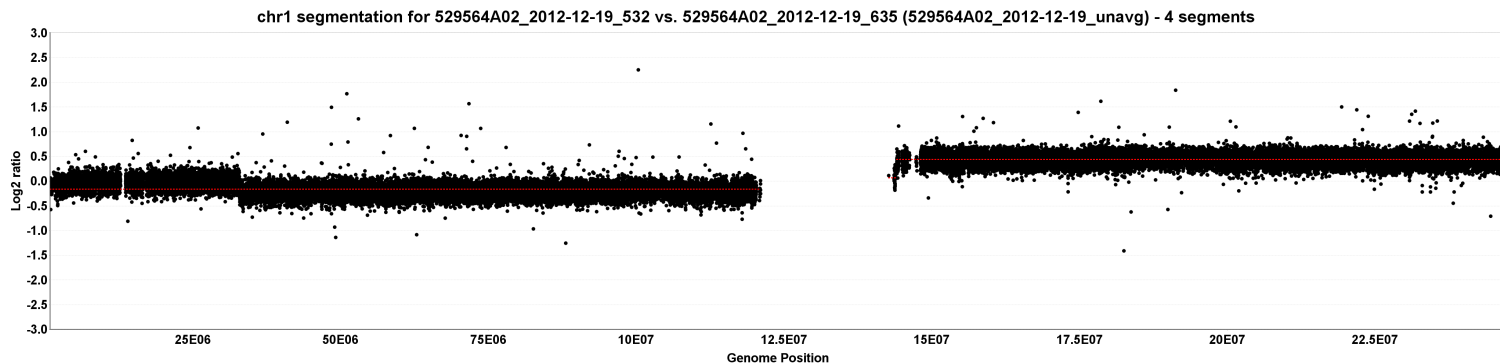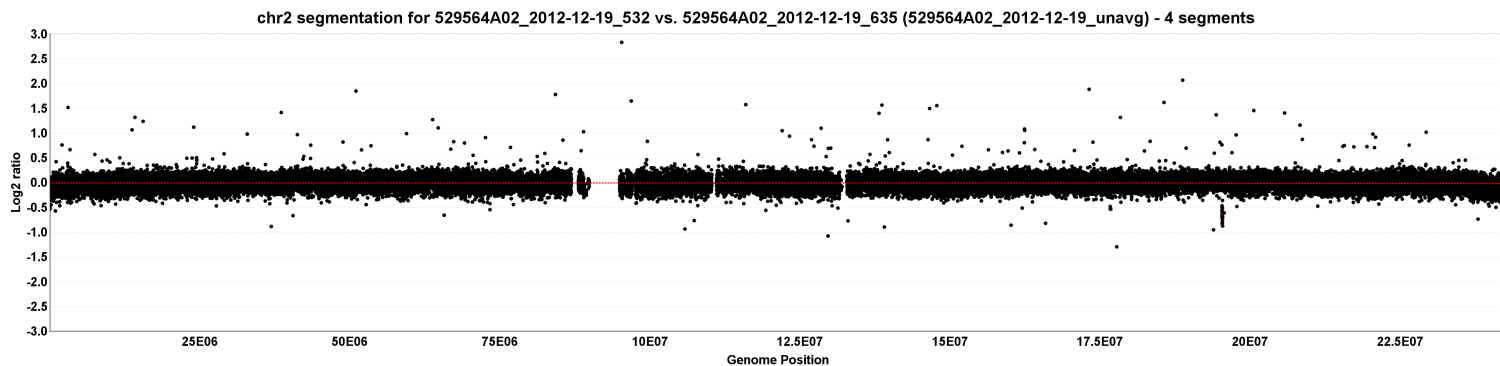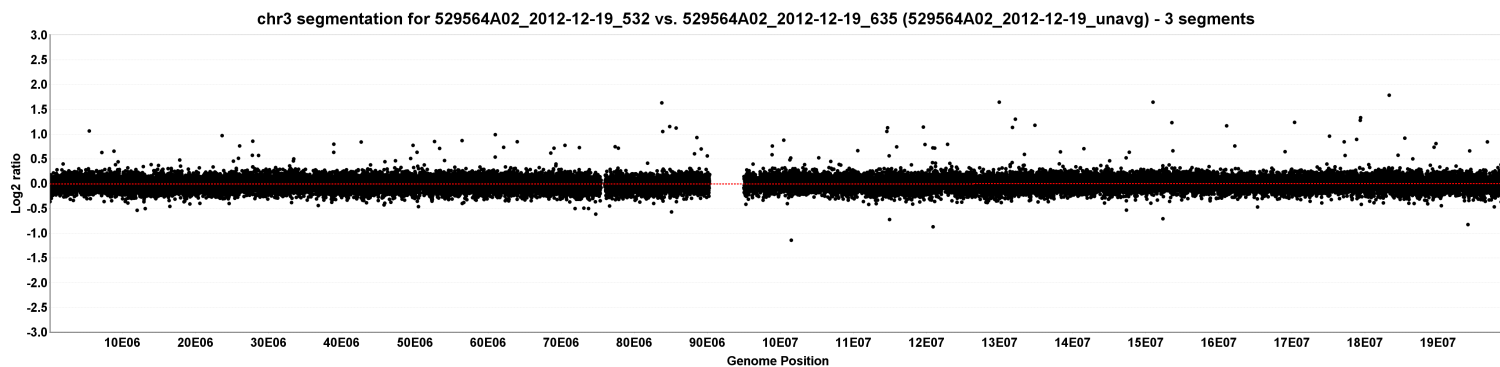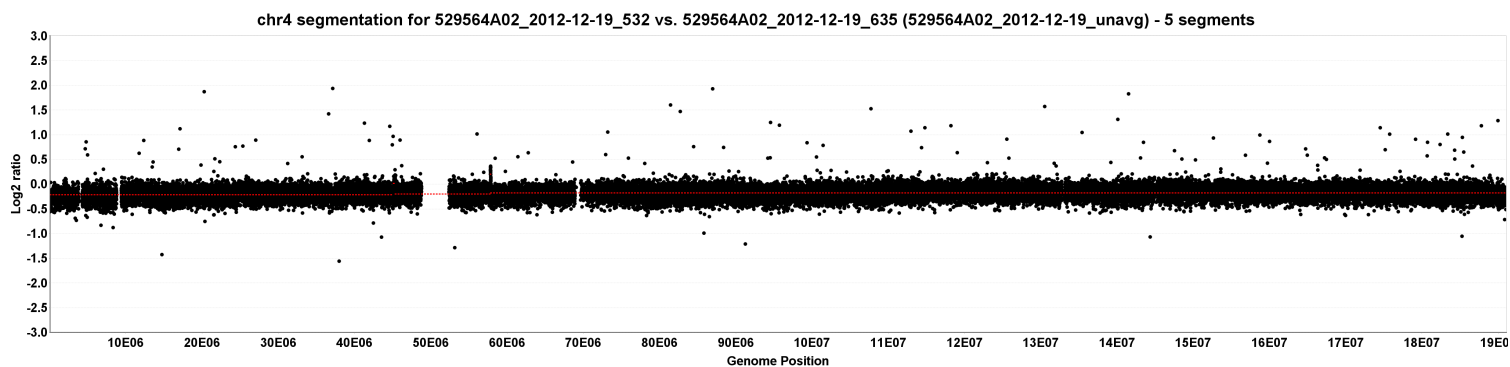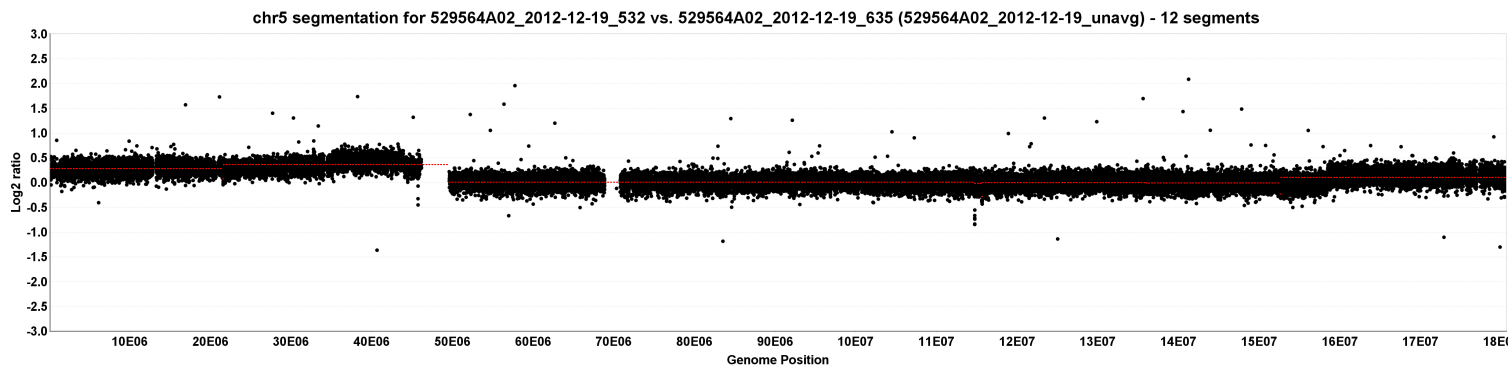

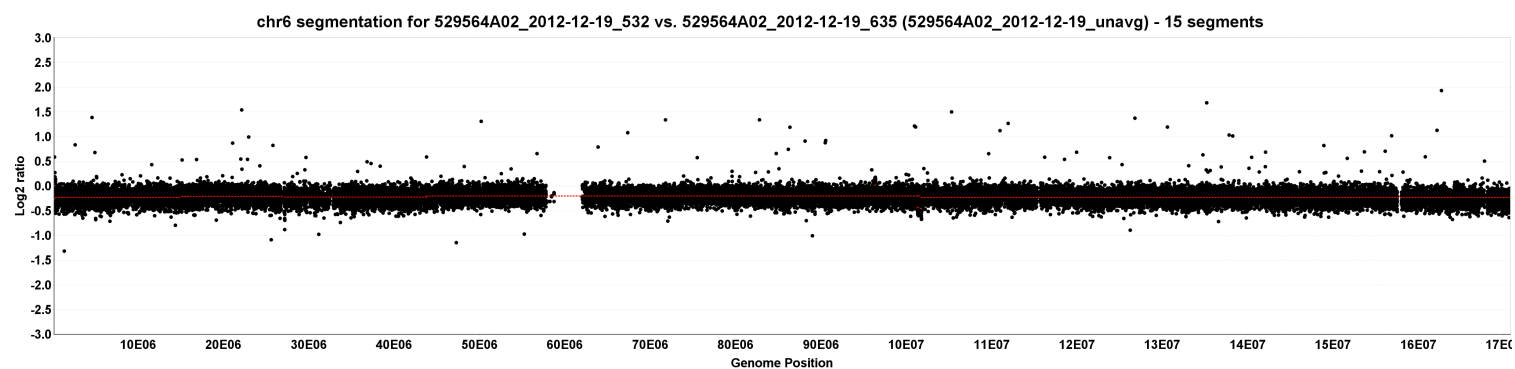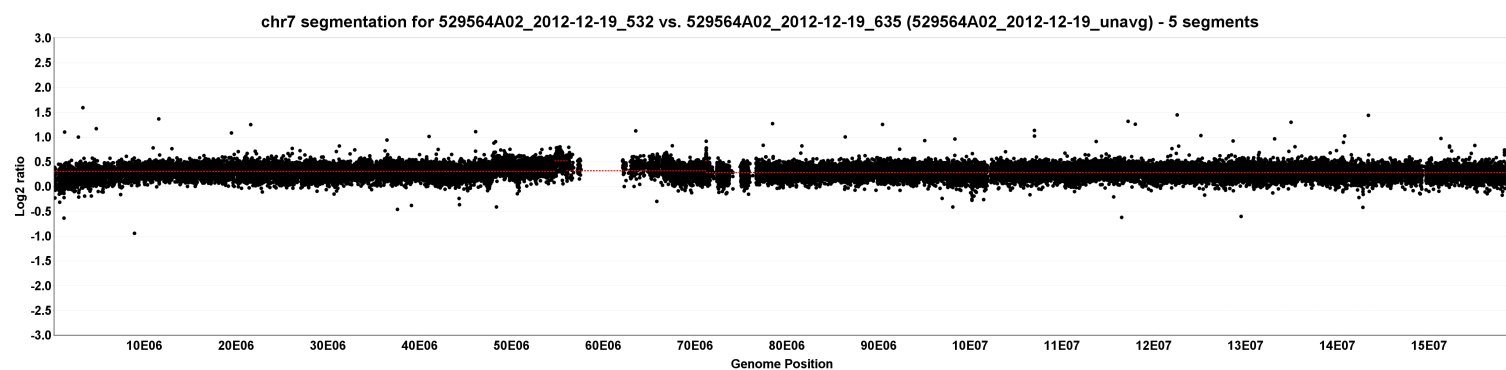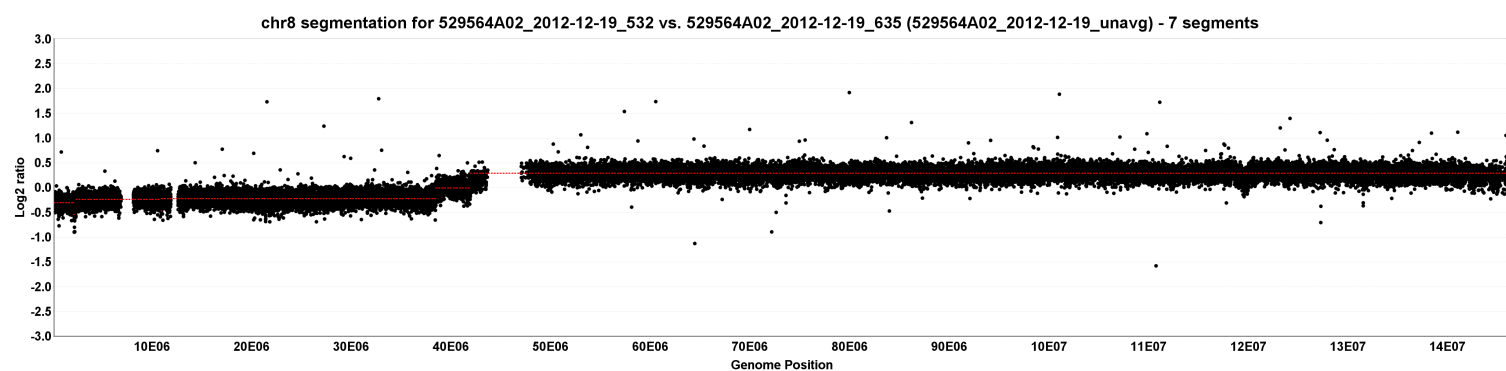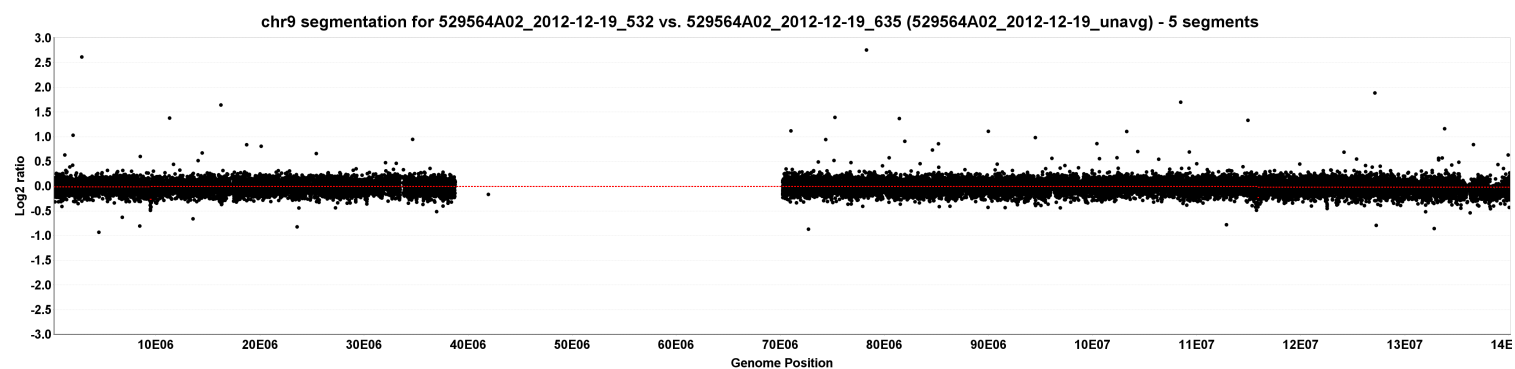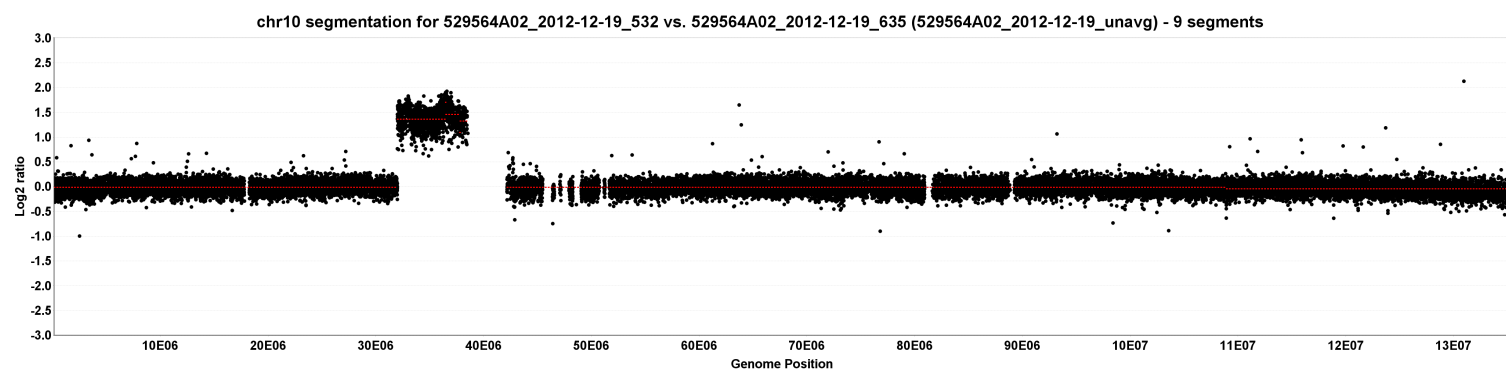

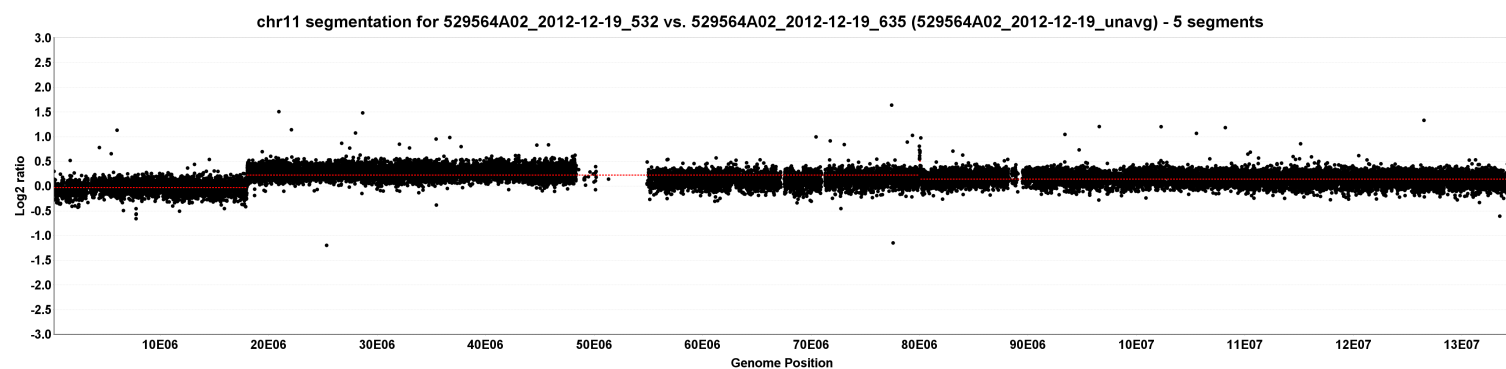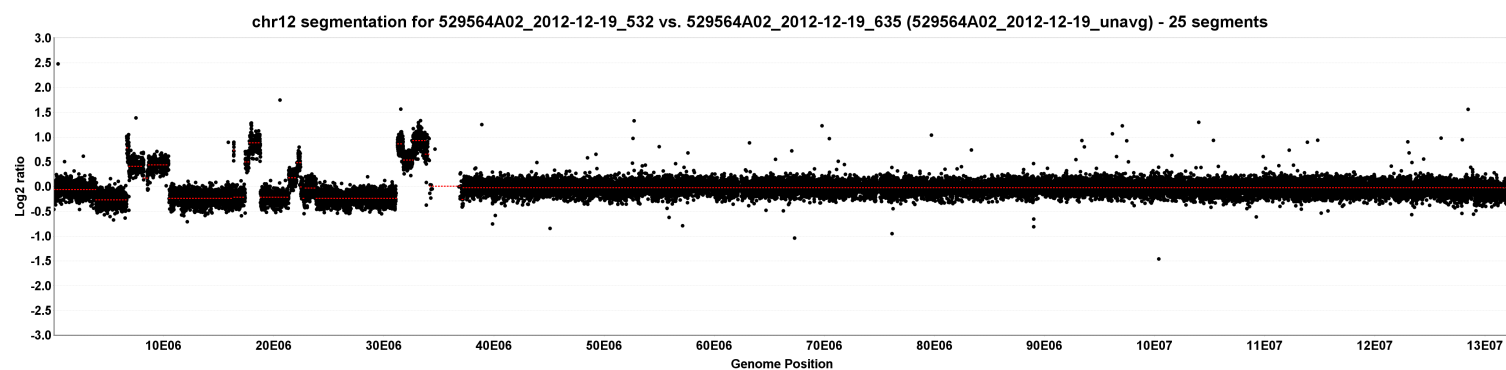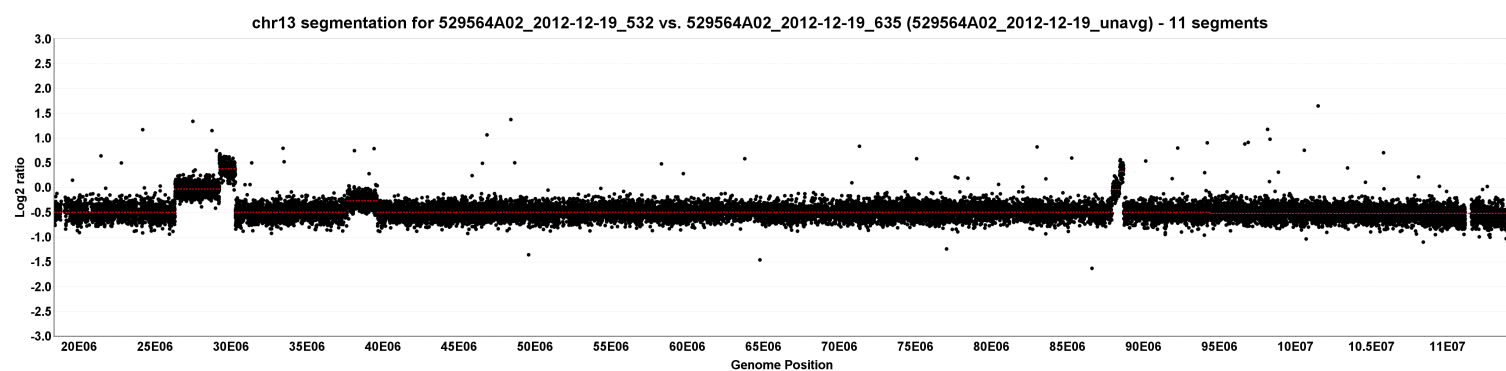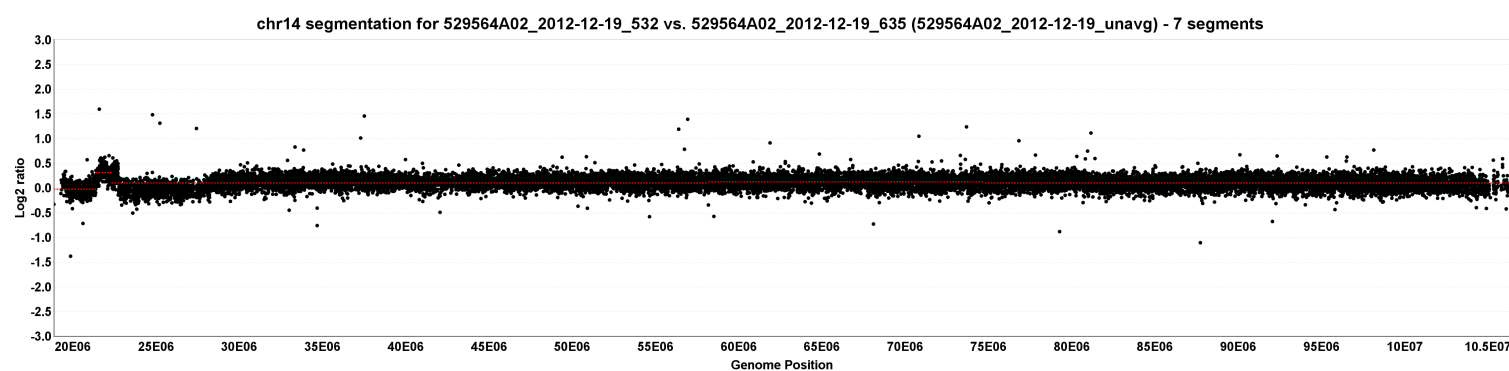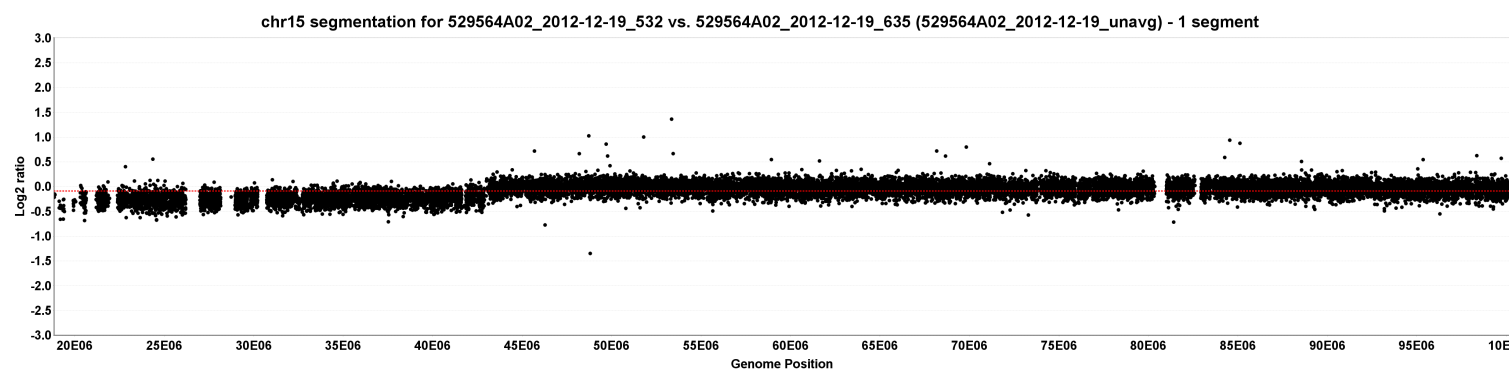

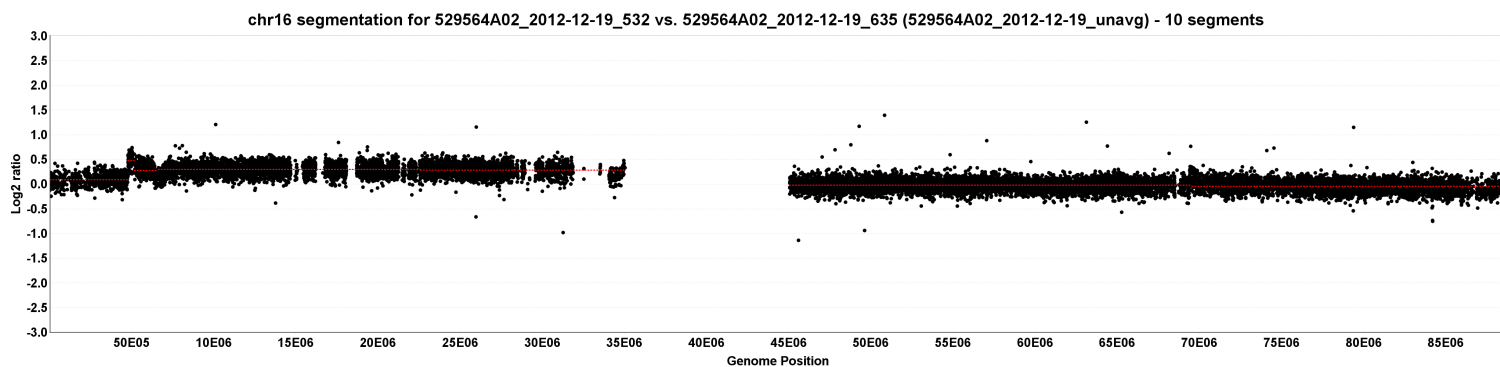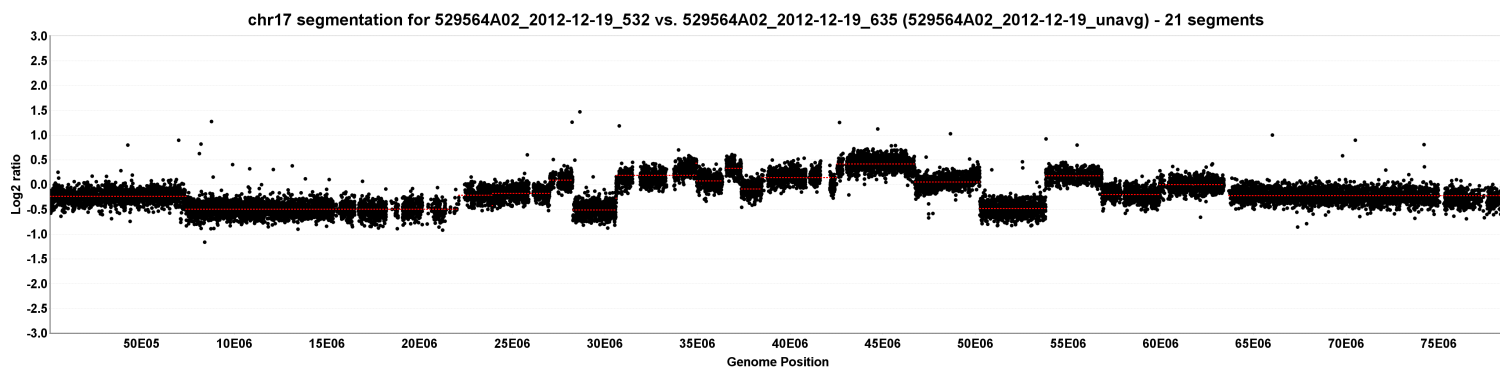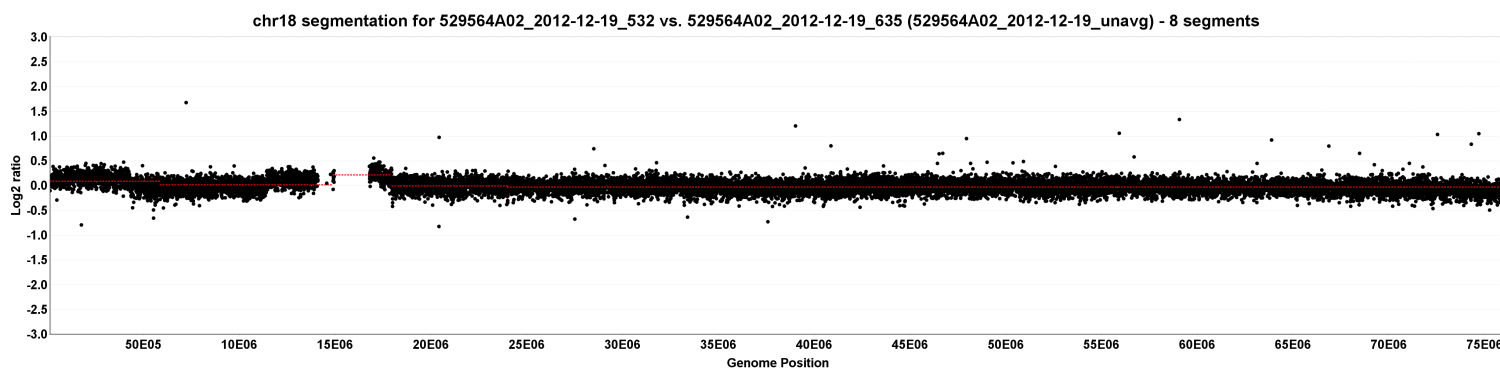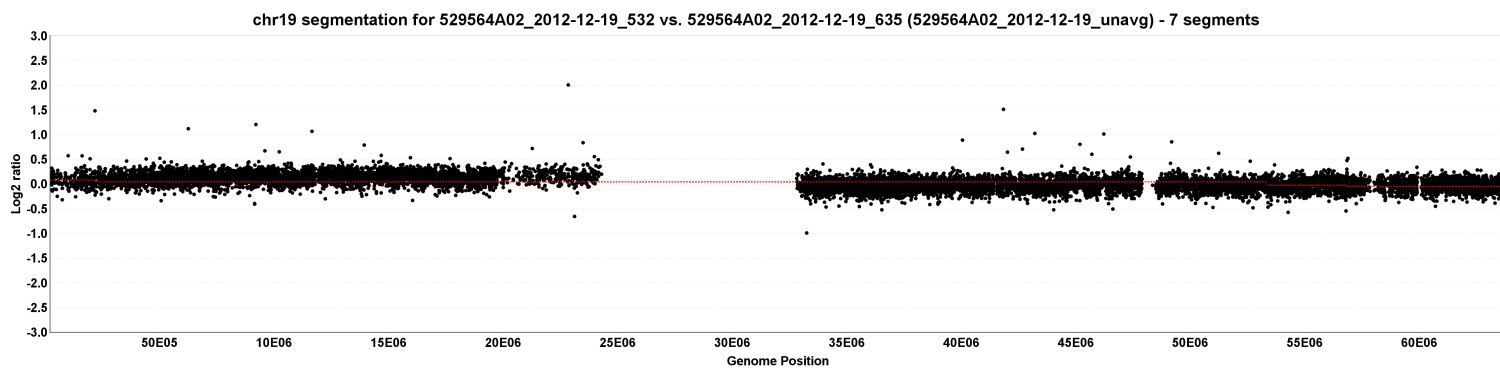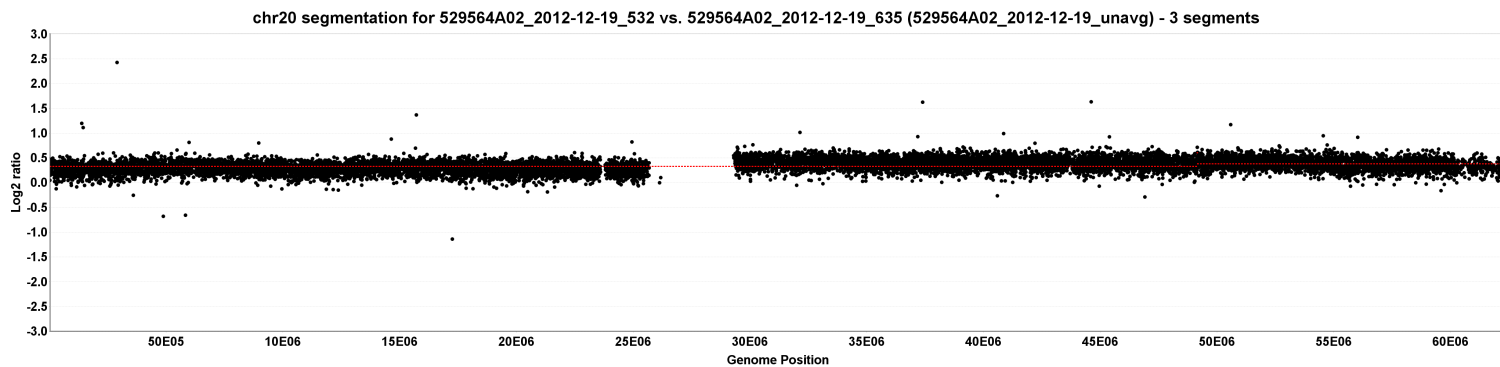

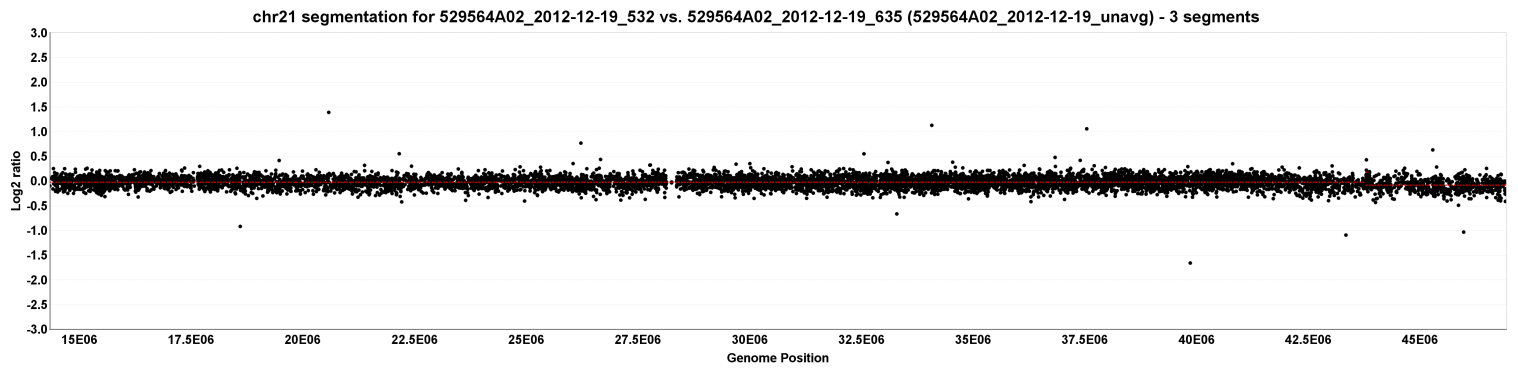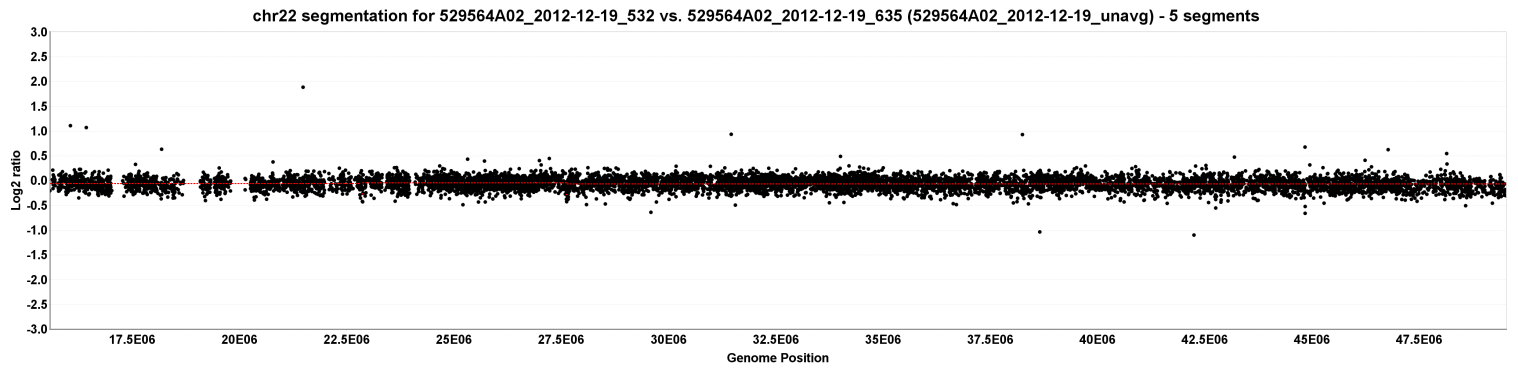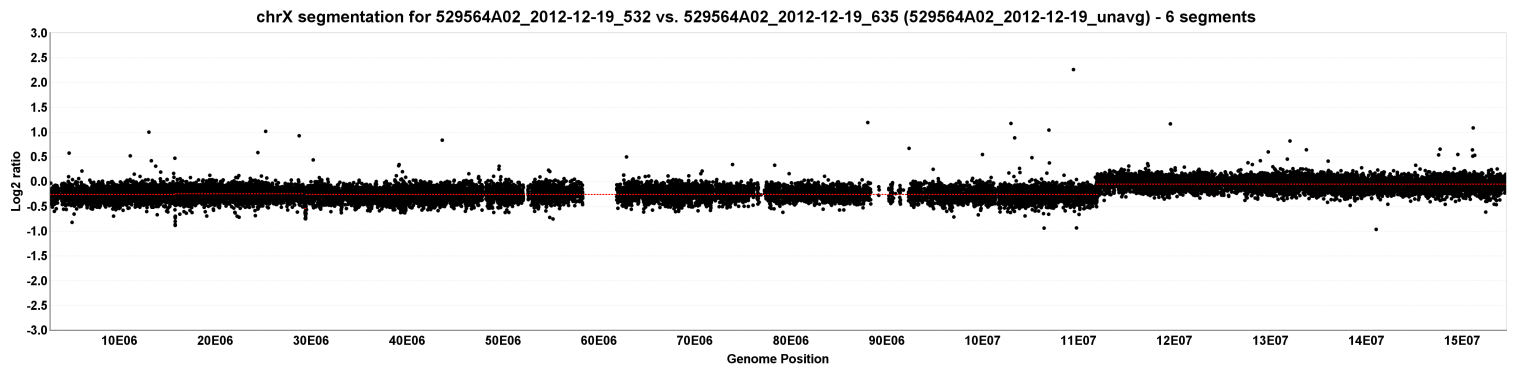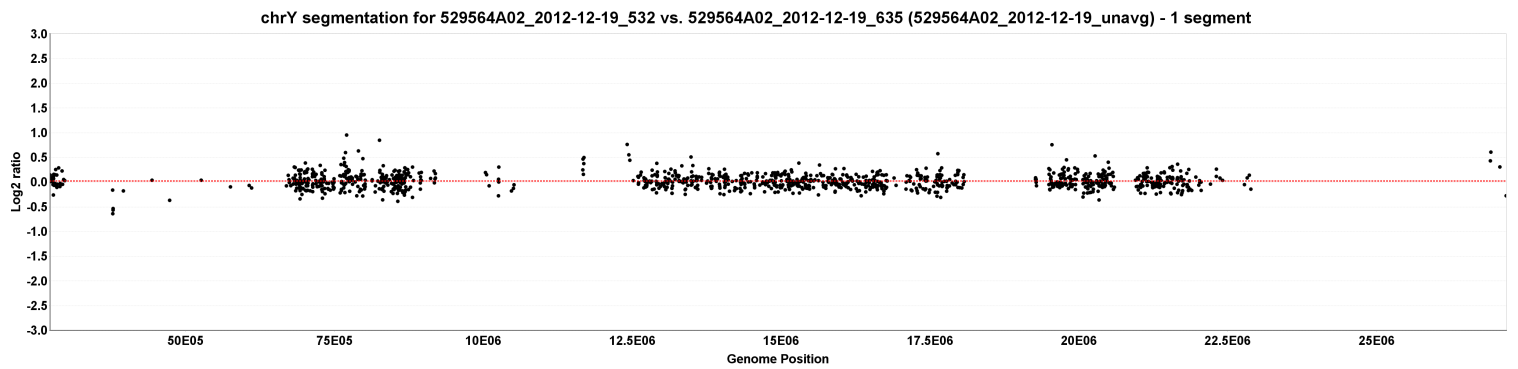

Supplement: S5 File — (PDF) [file pone.0169098.s005.pdf]

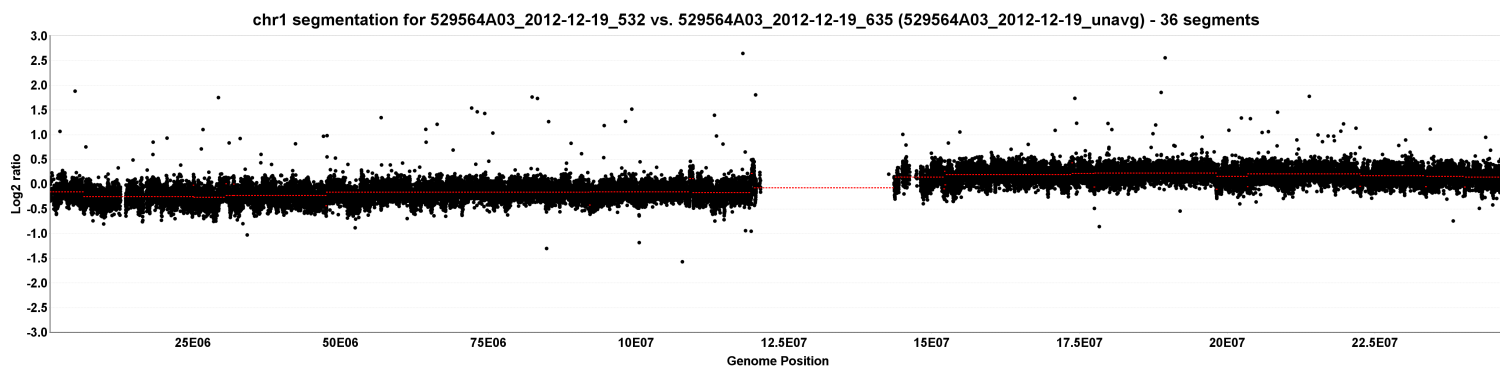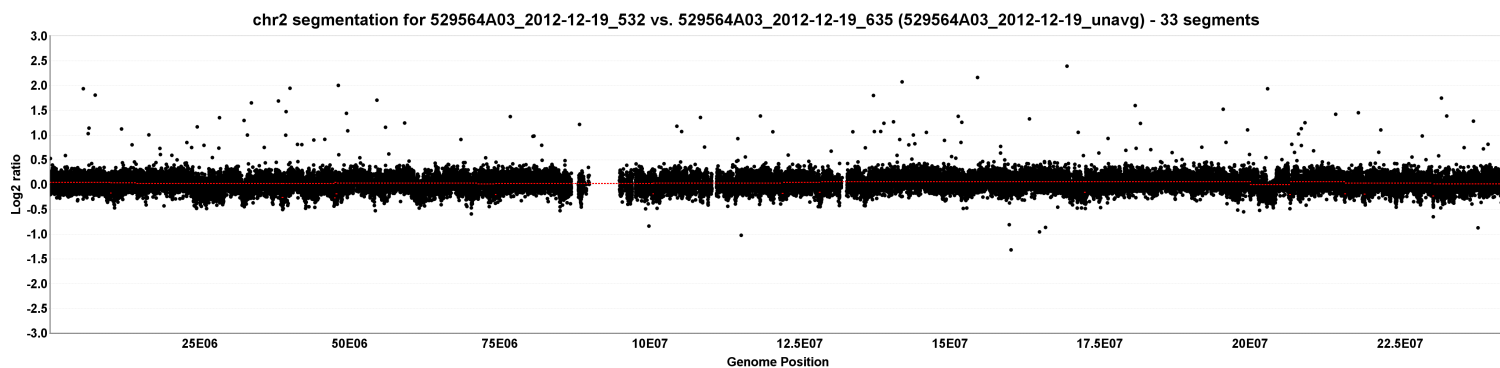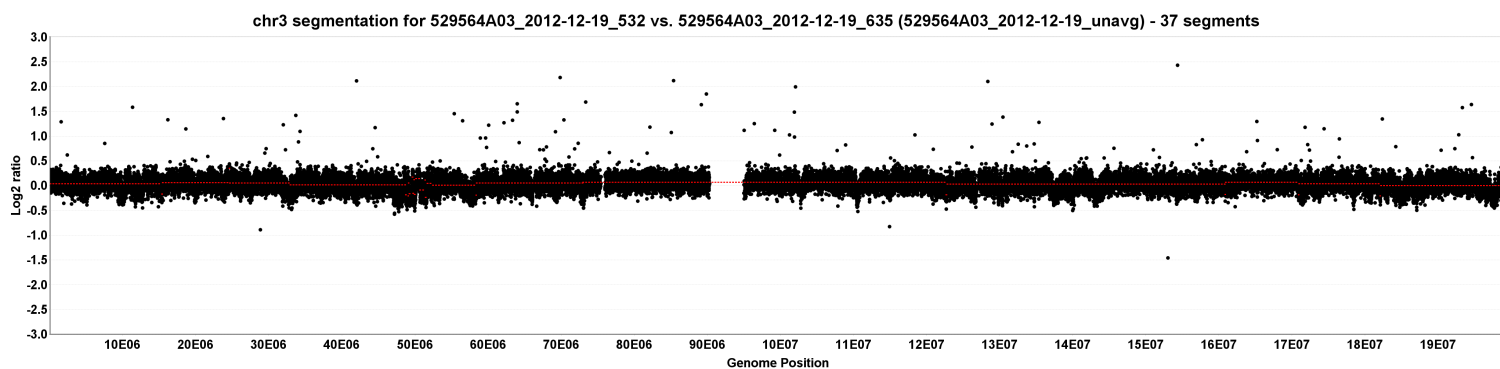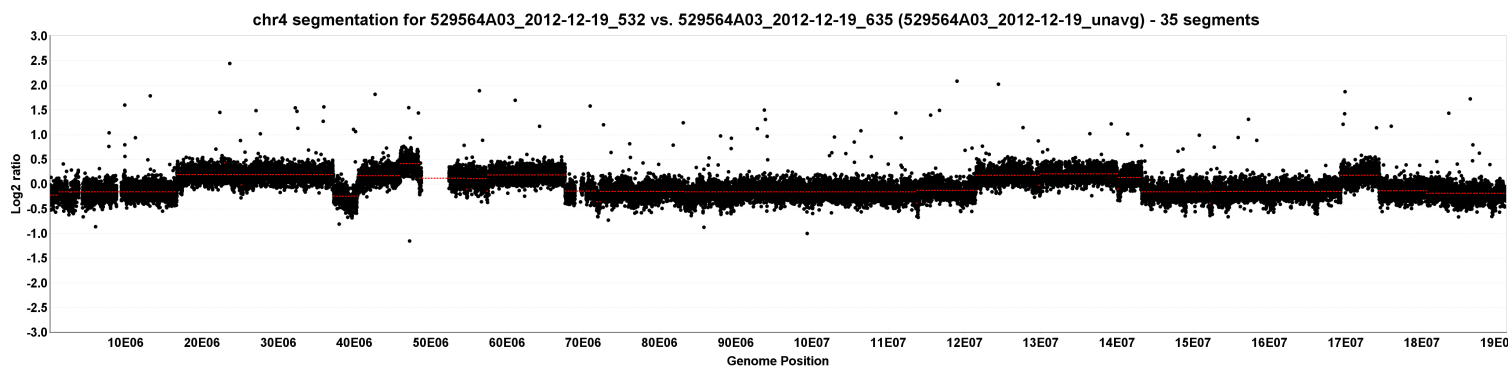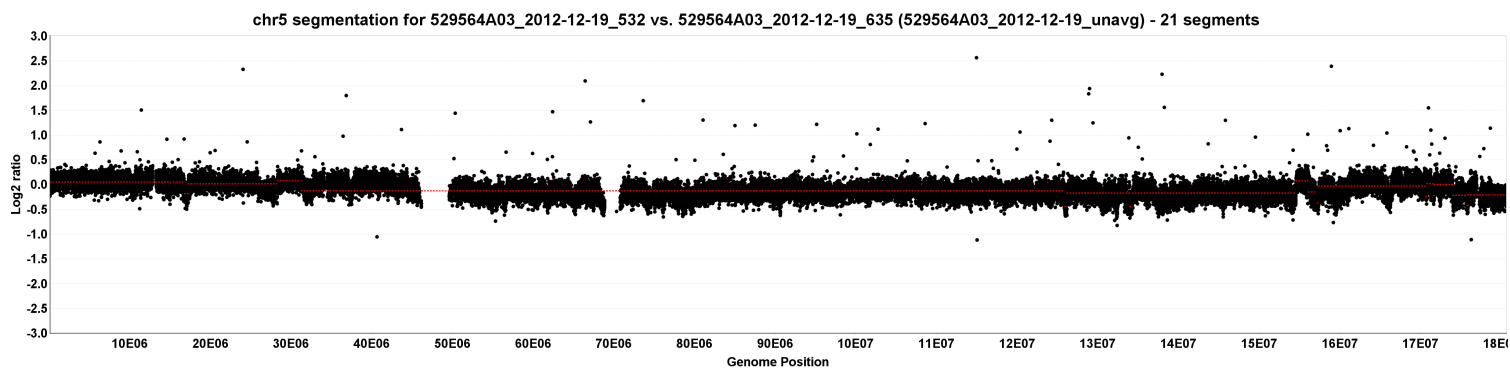

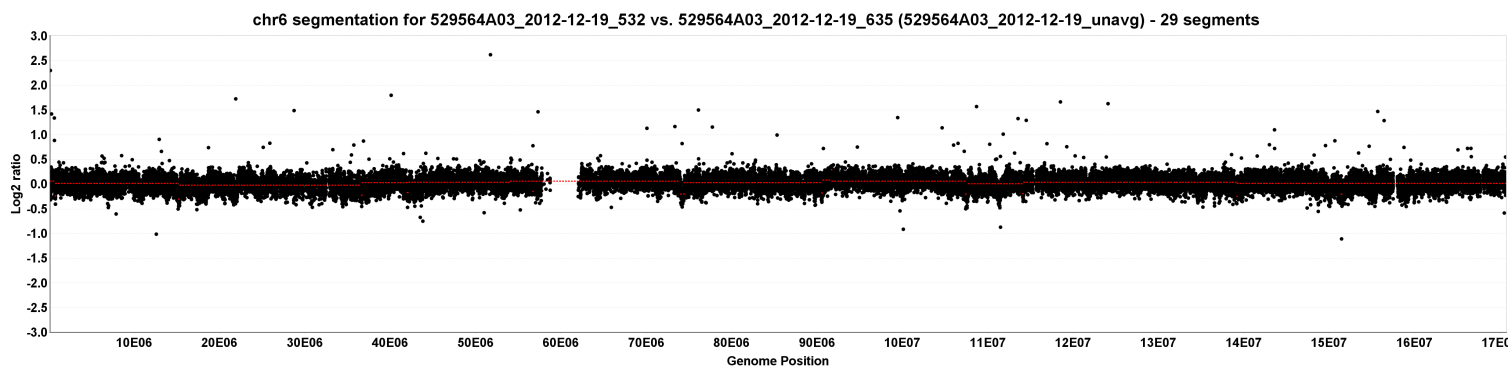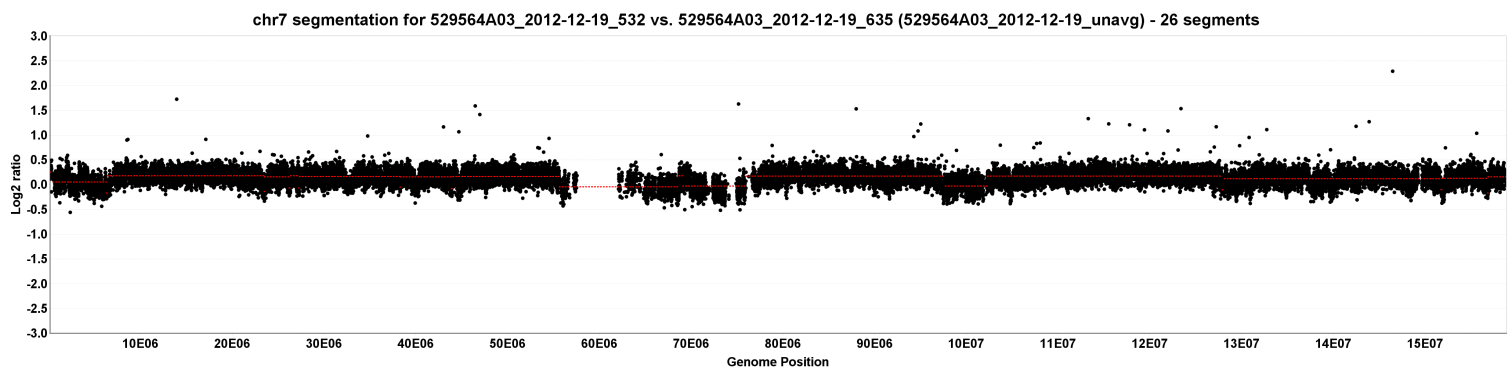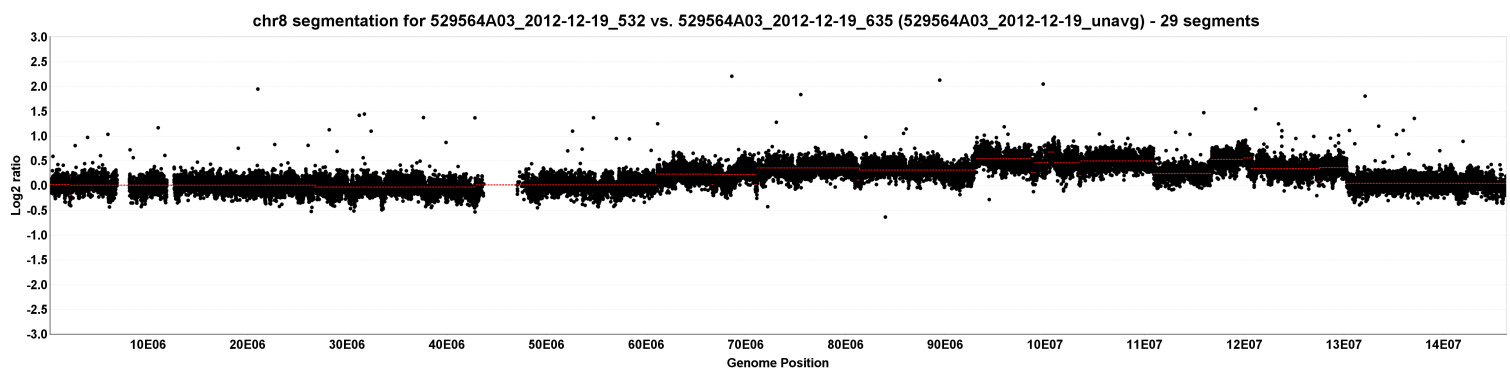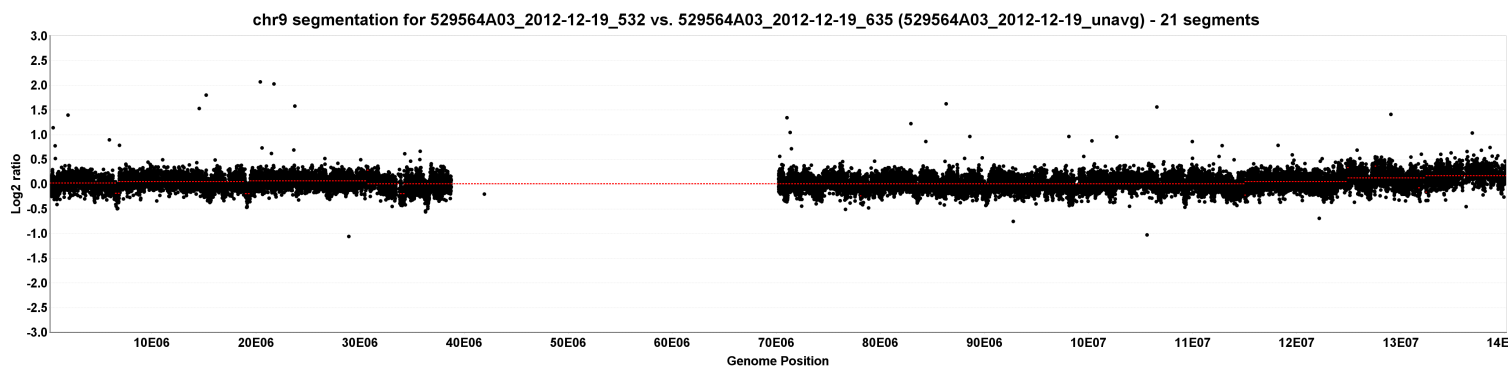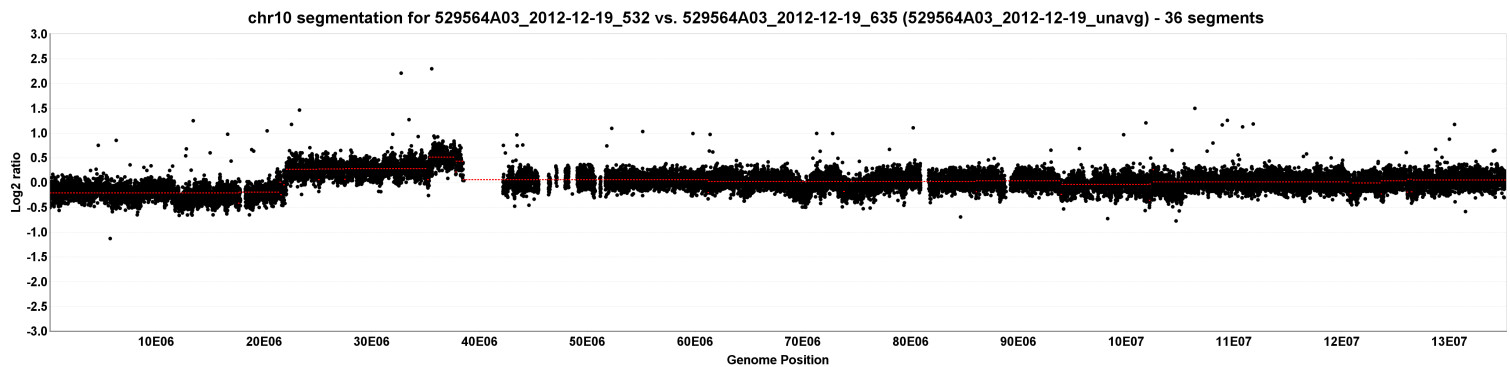

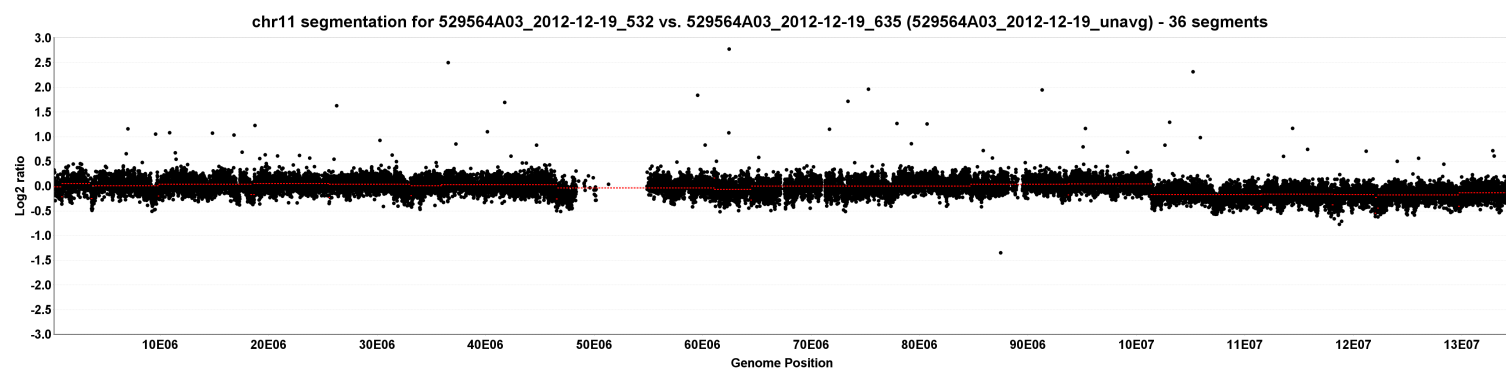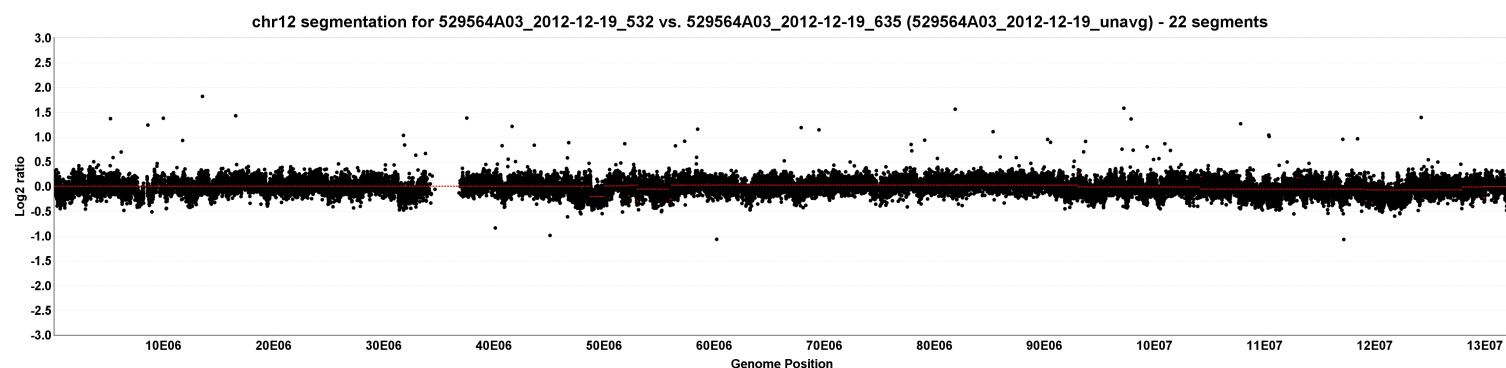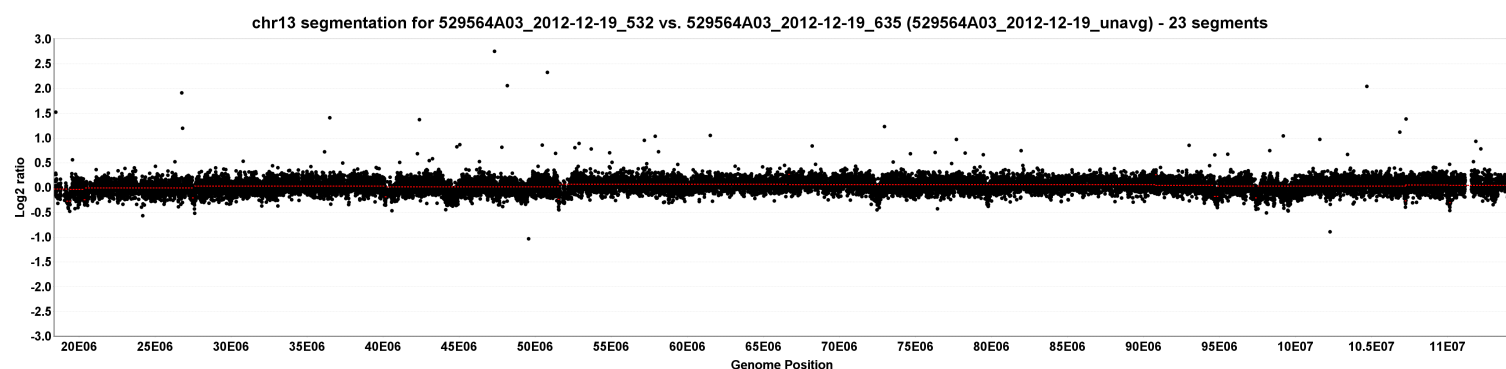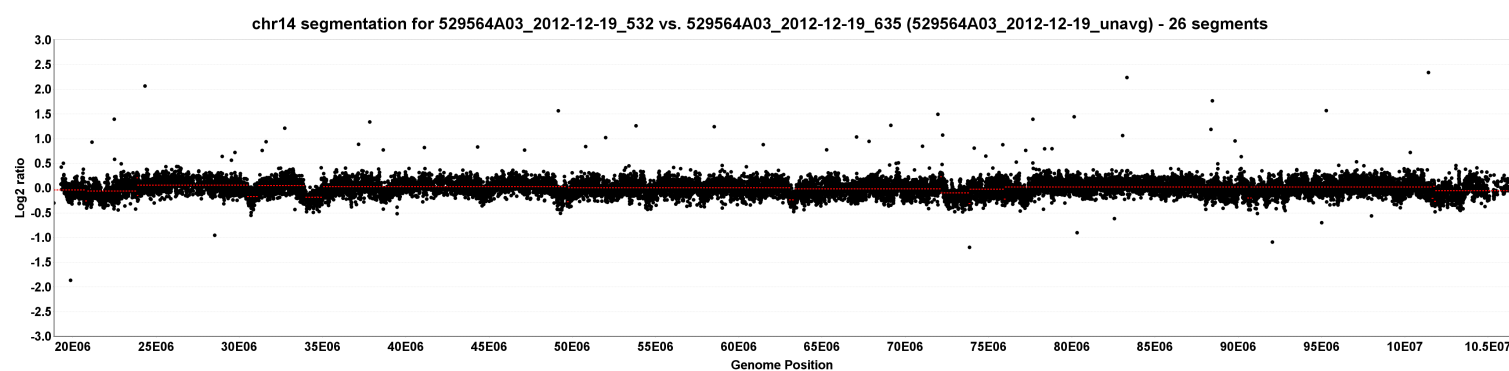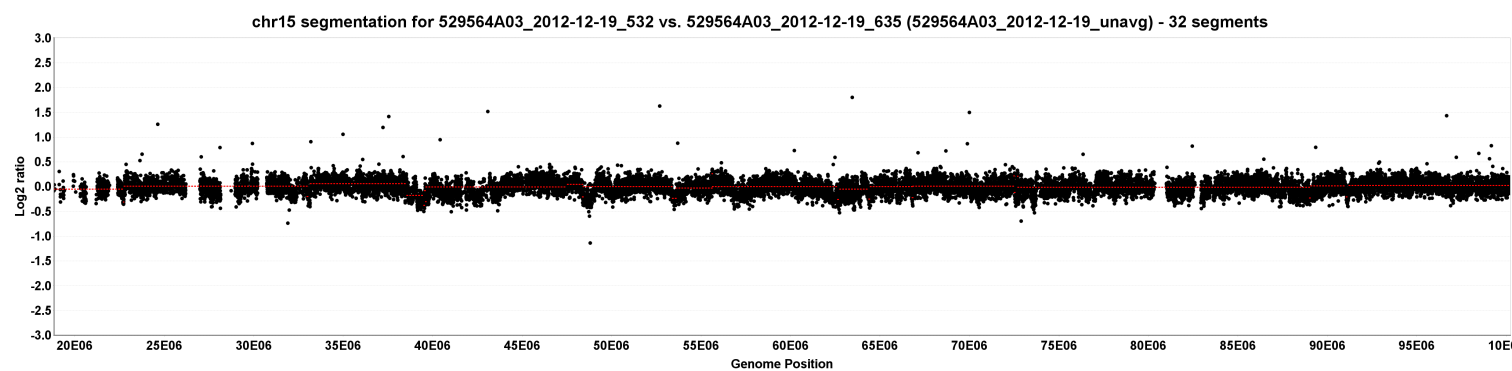

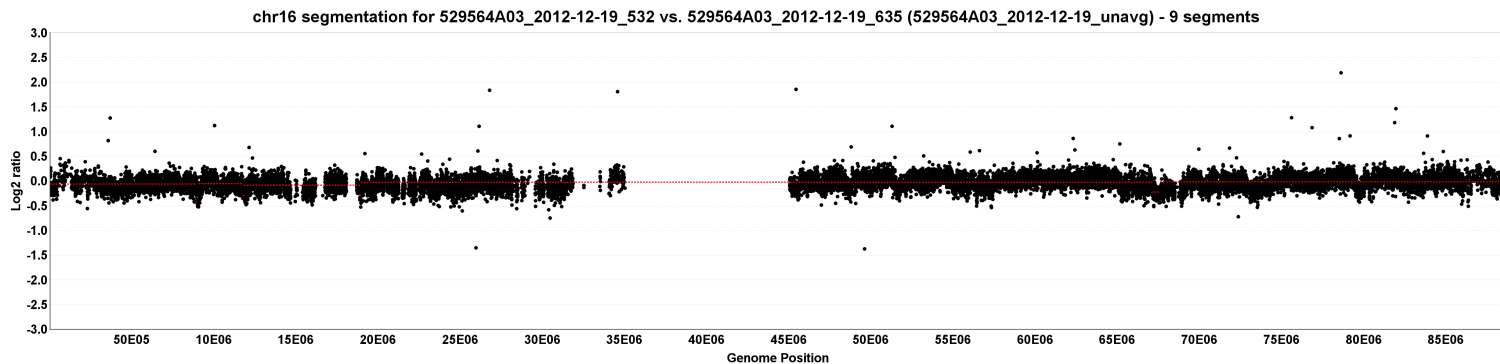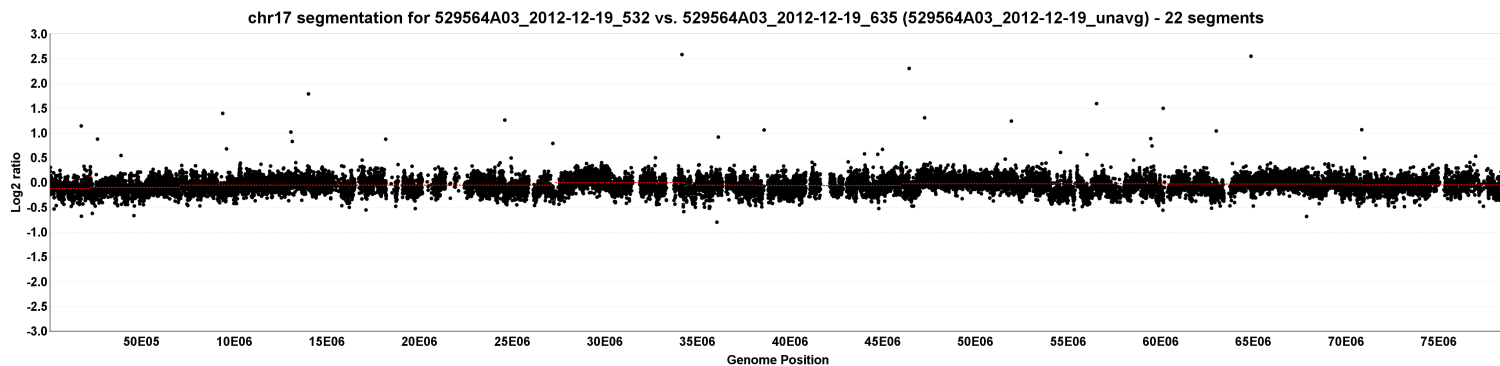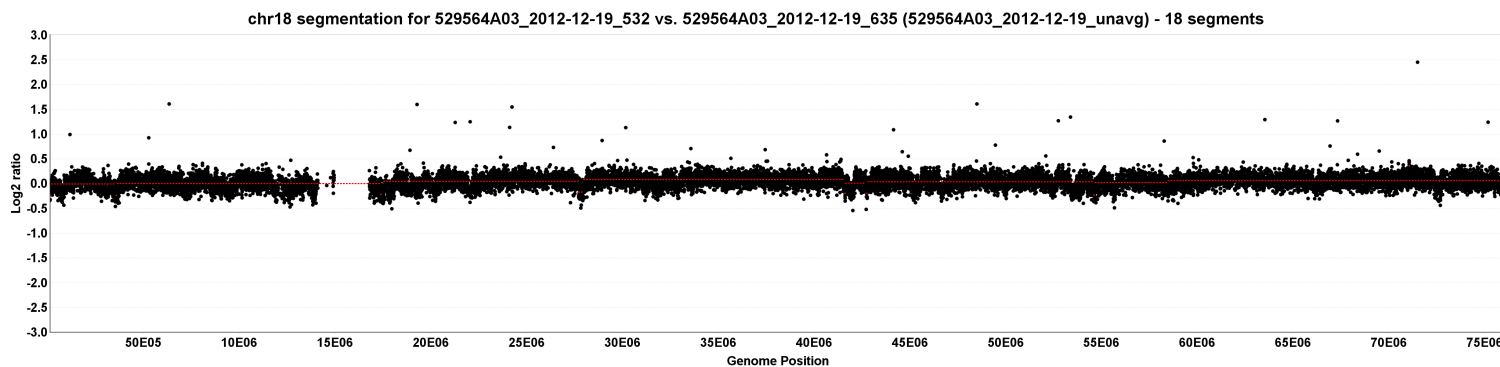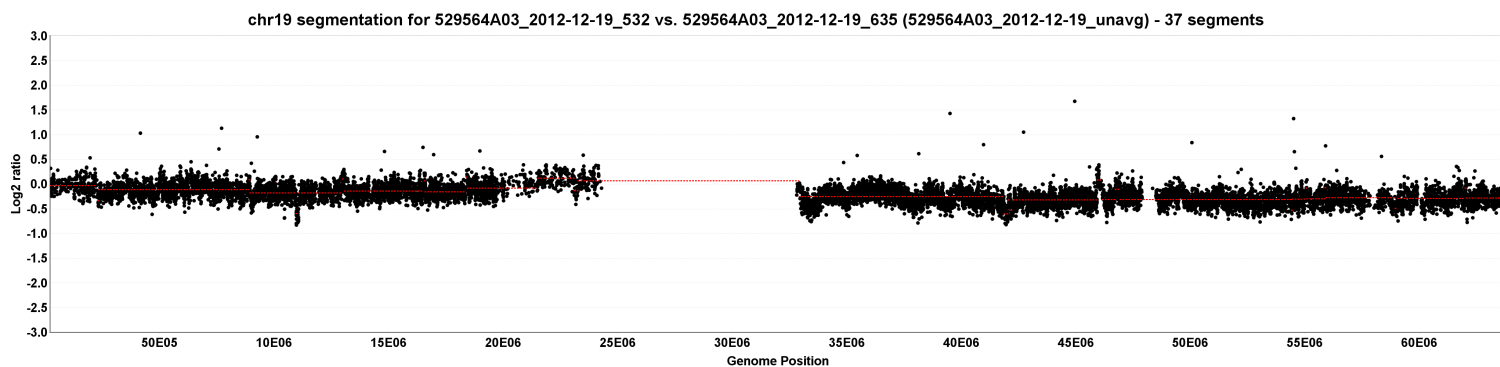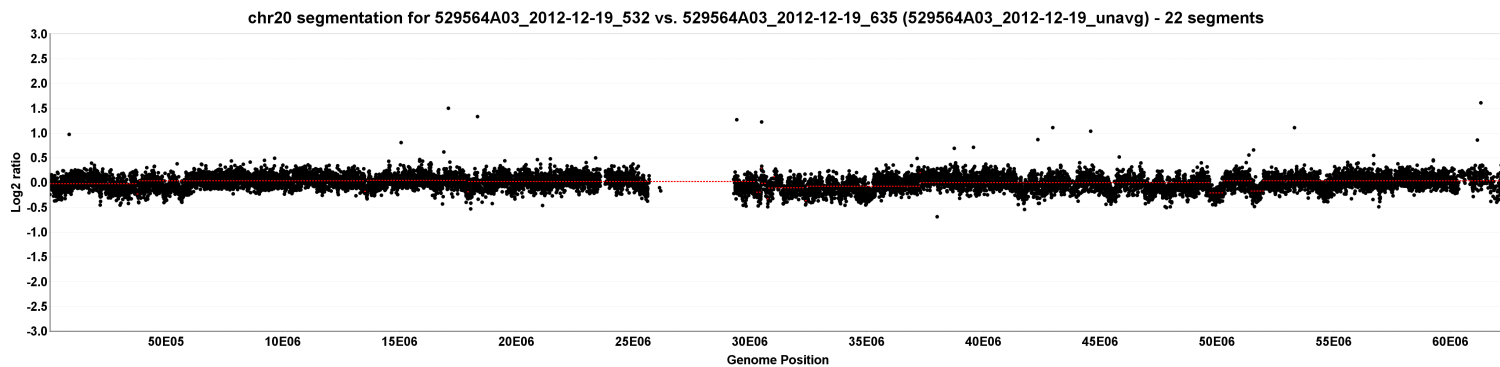

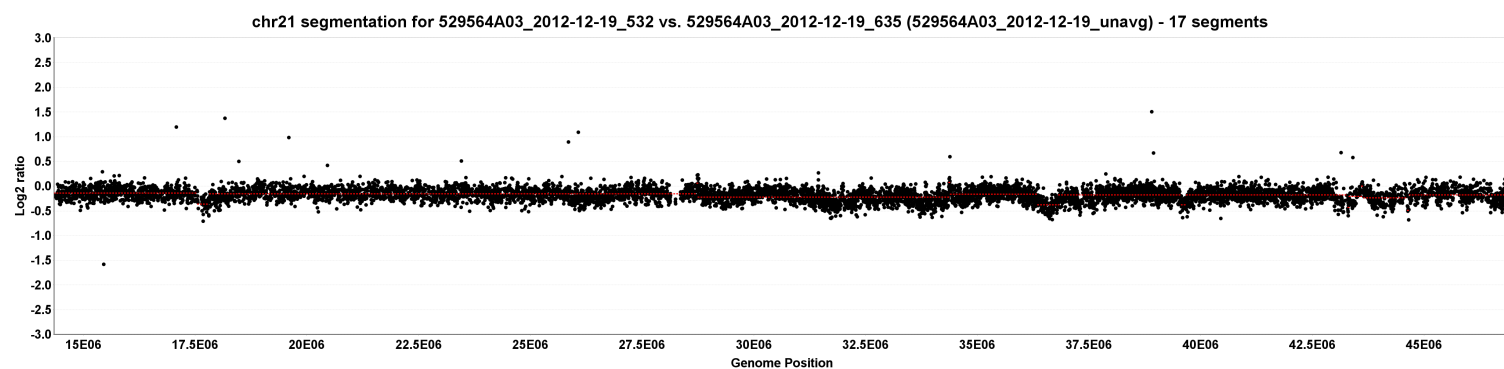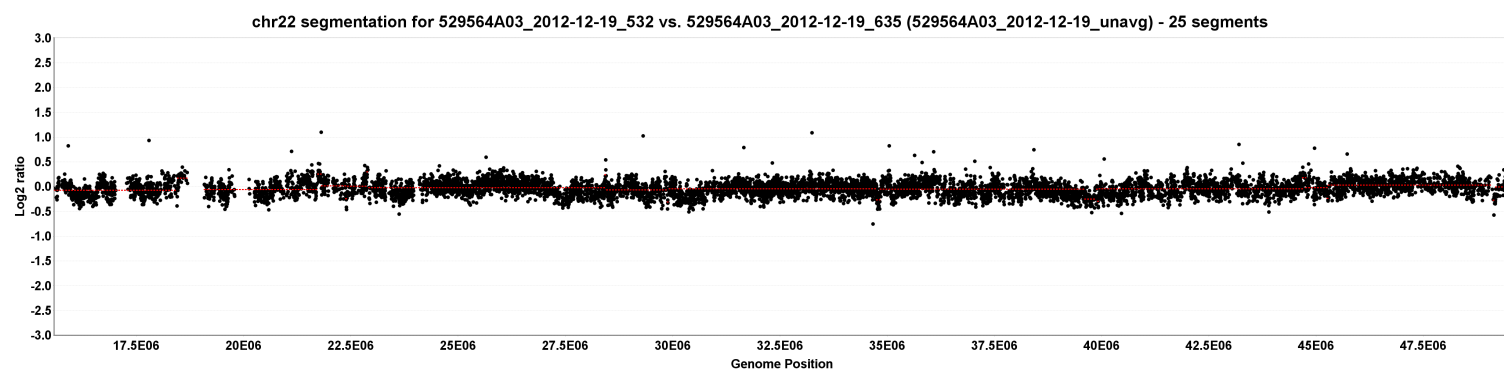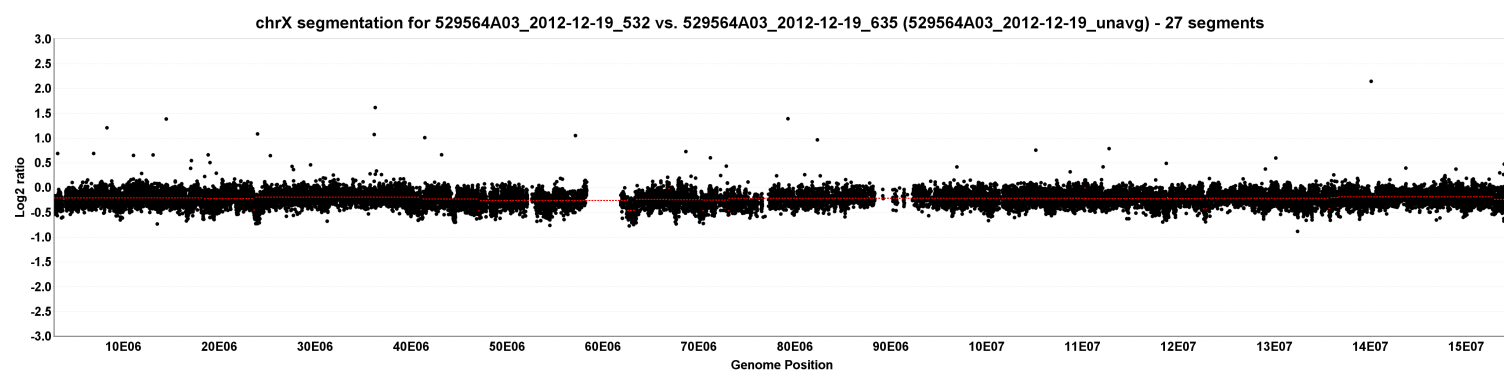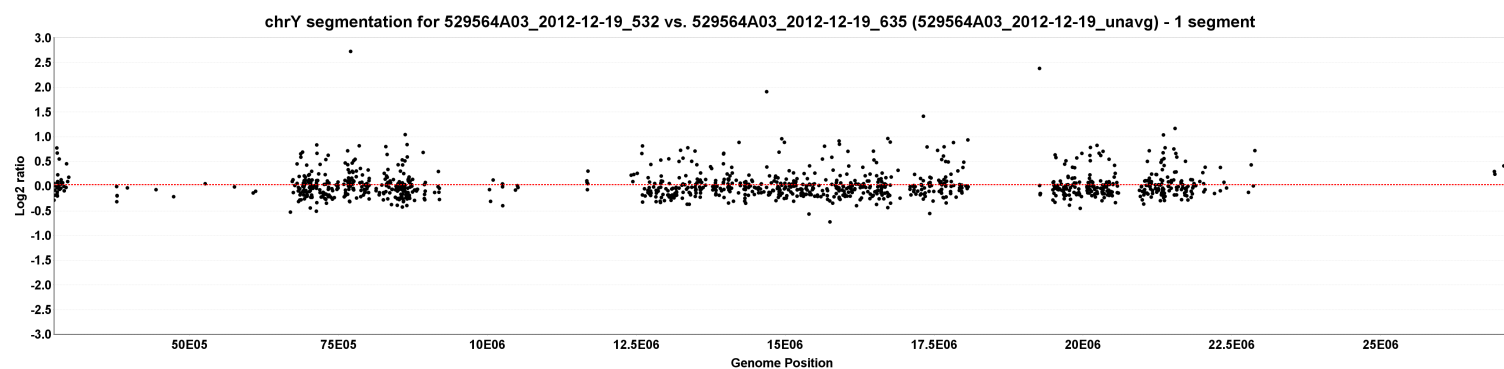

Supplement: S6 File — (PDF) [file pone.0169098.s006.pdf]

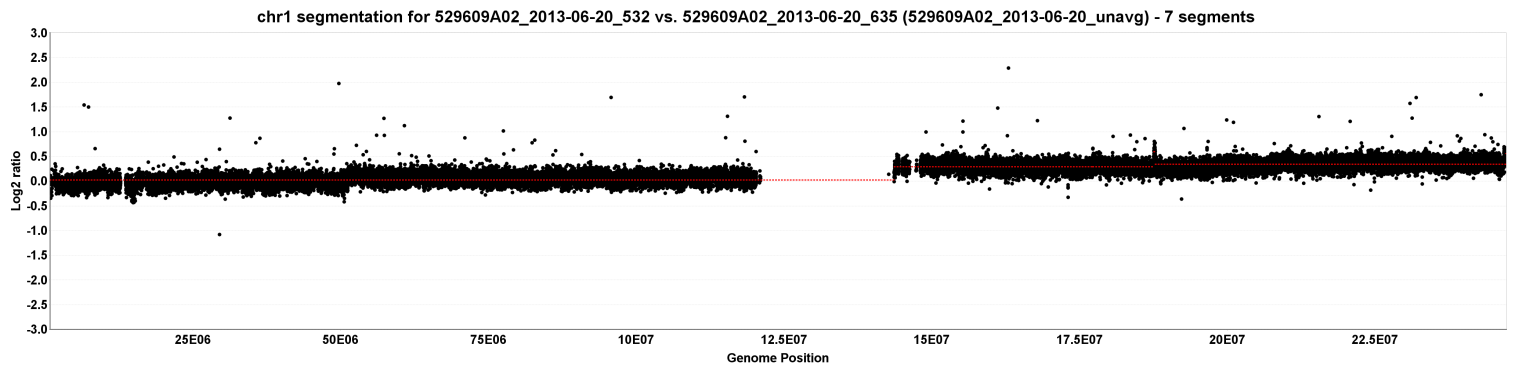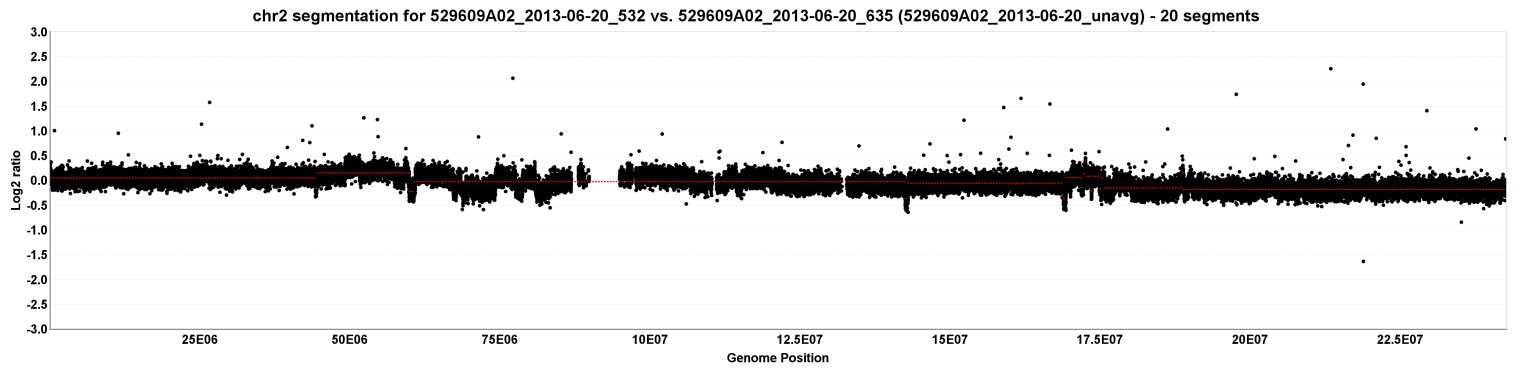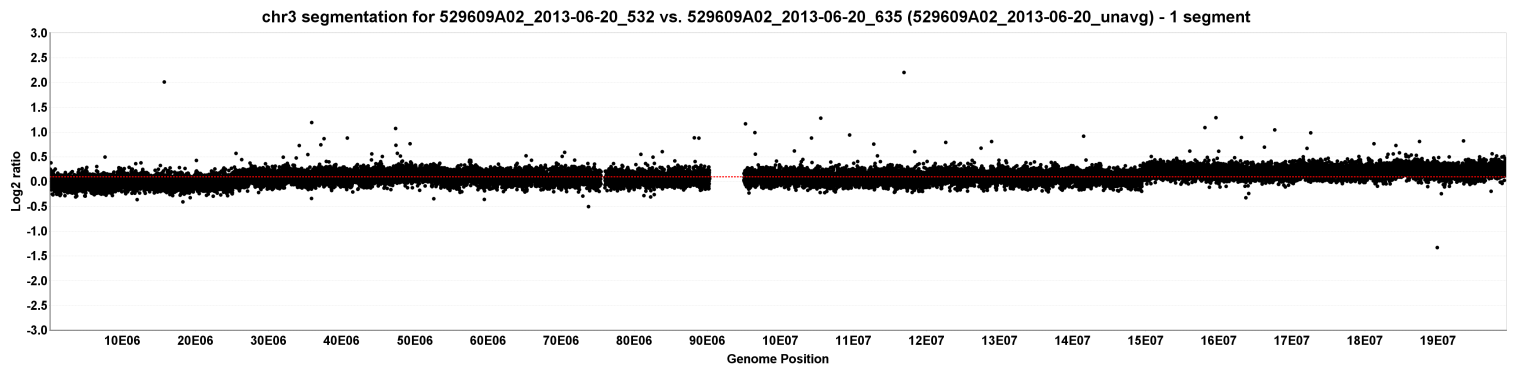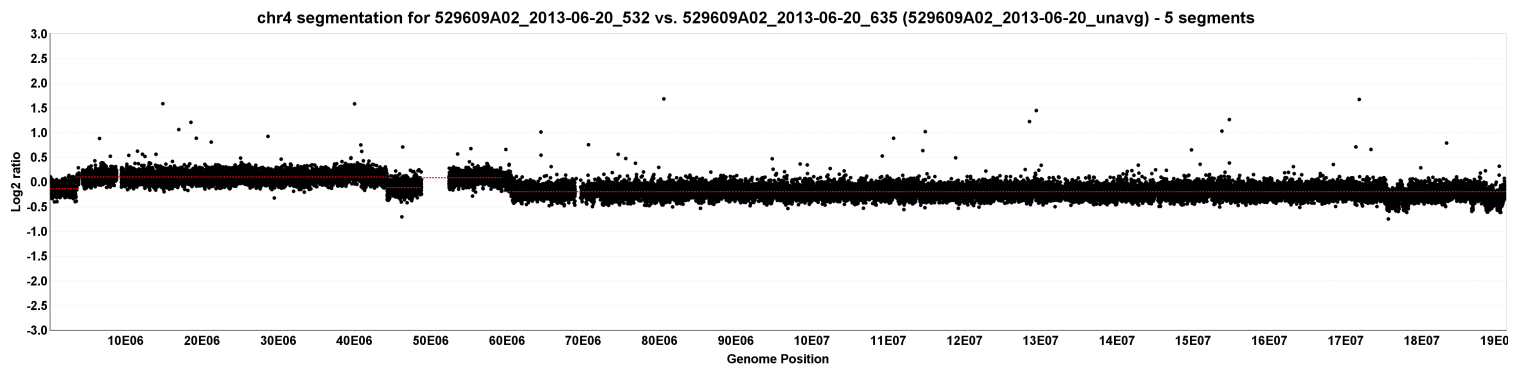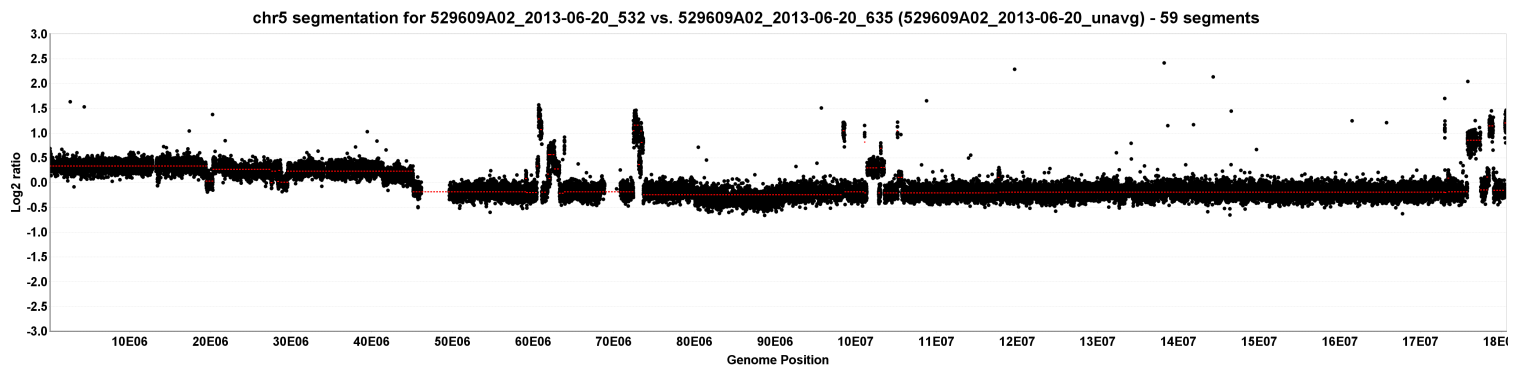

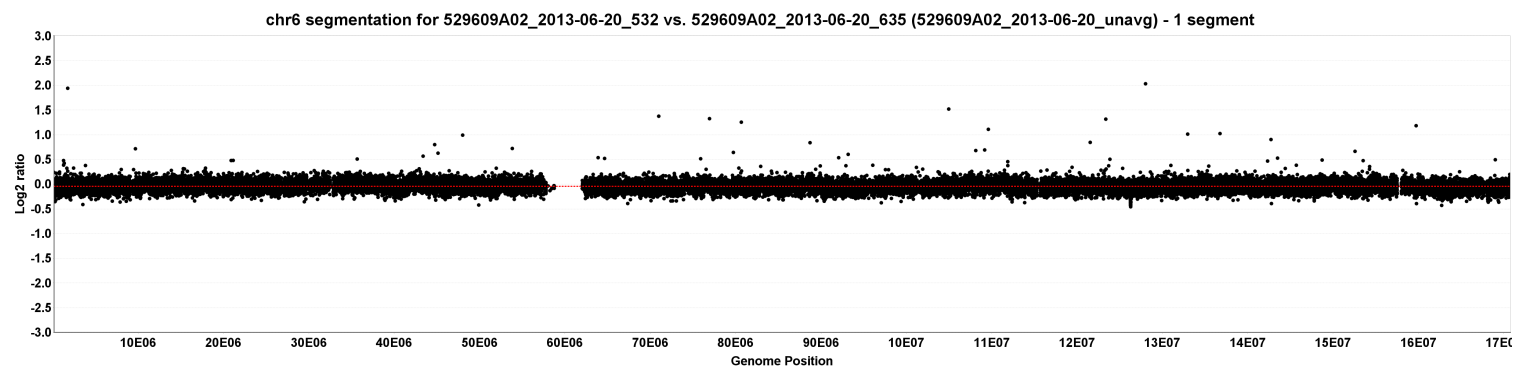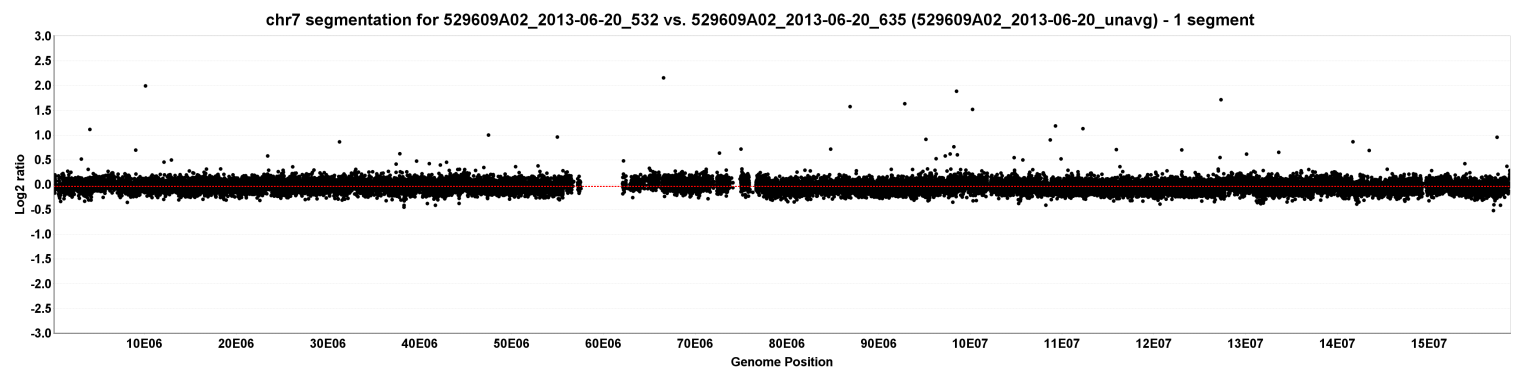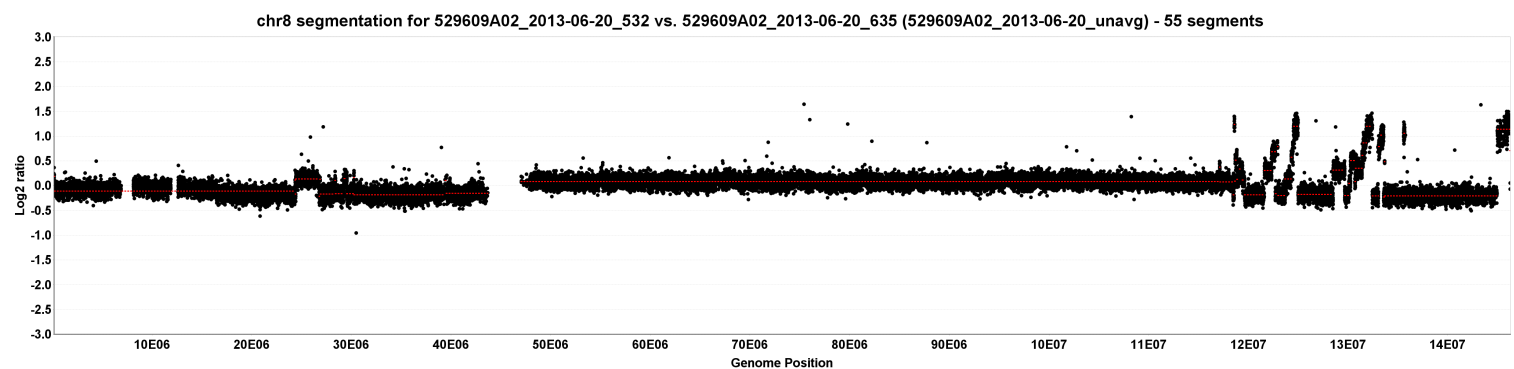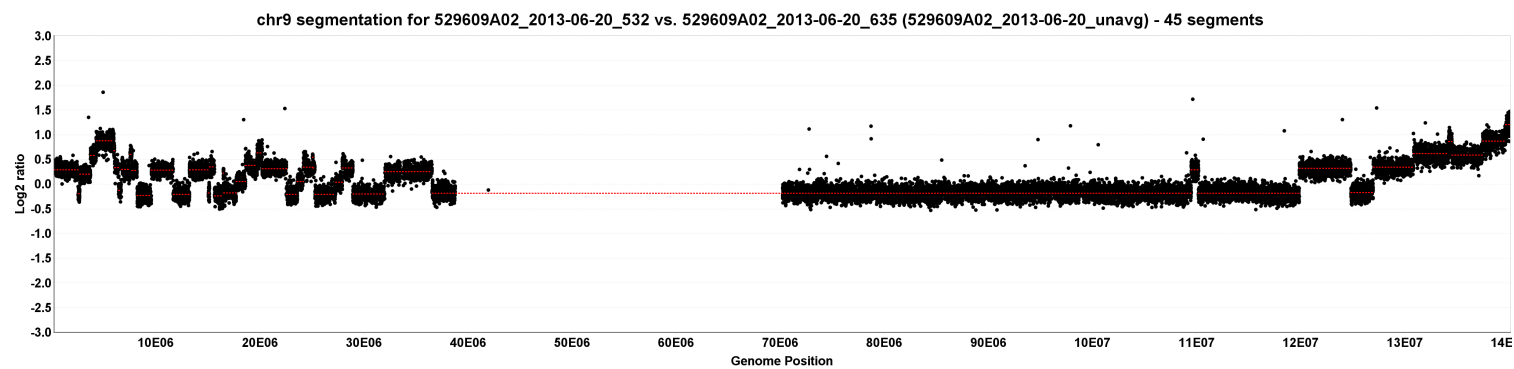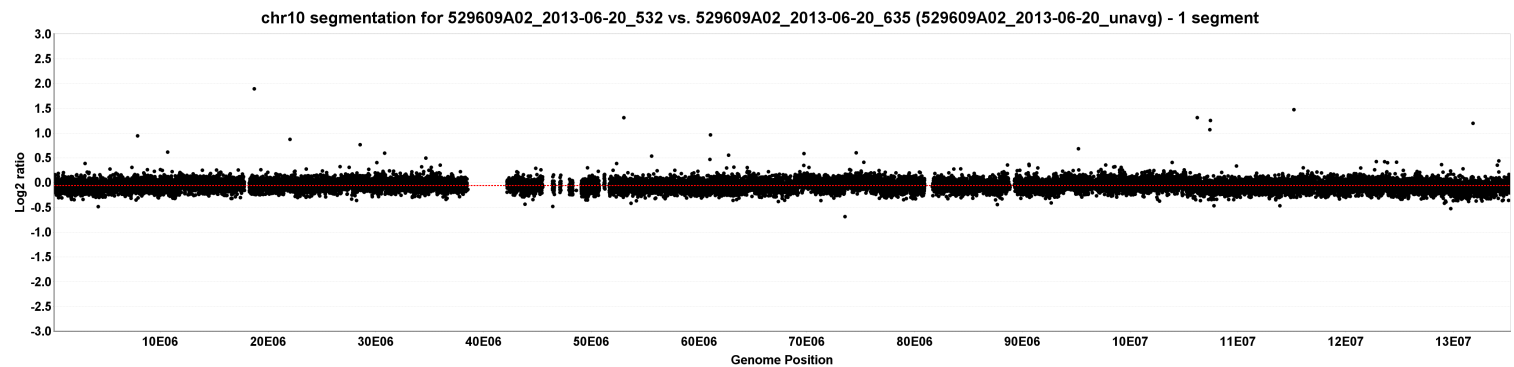

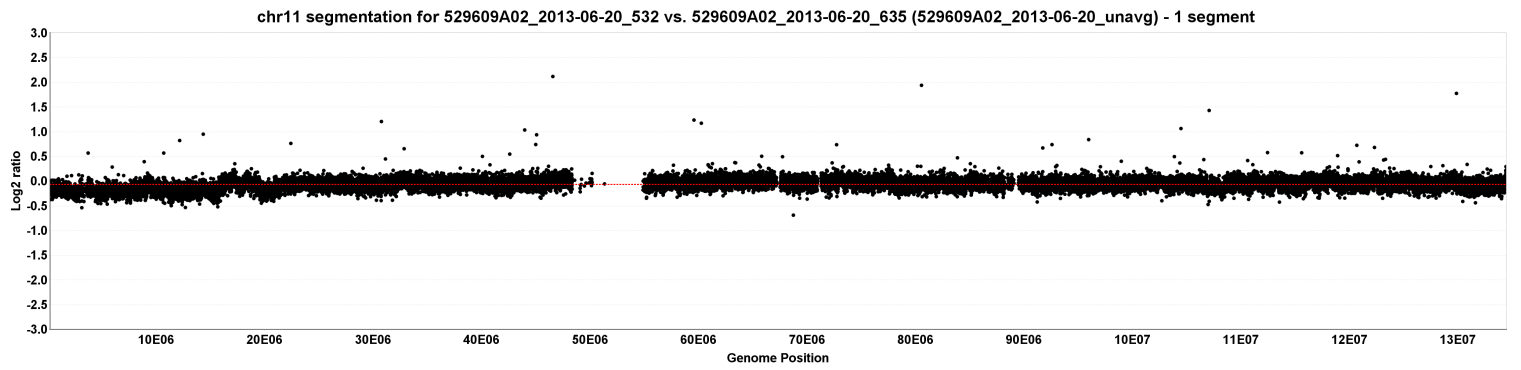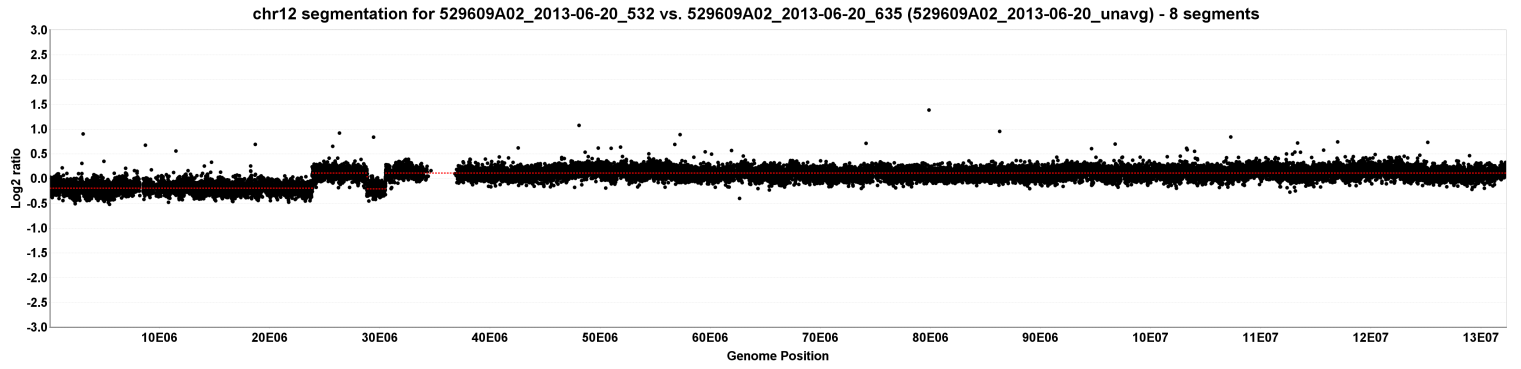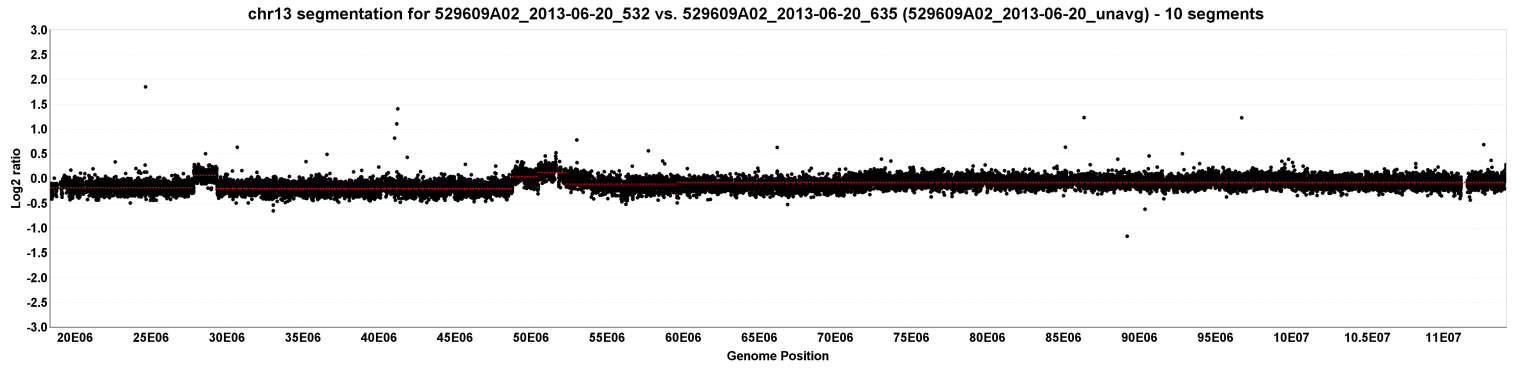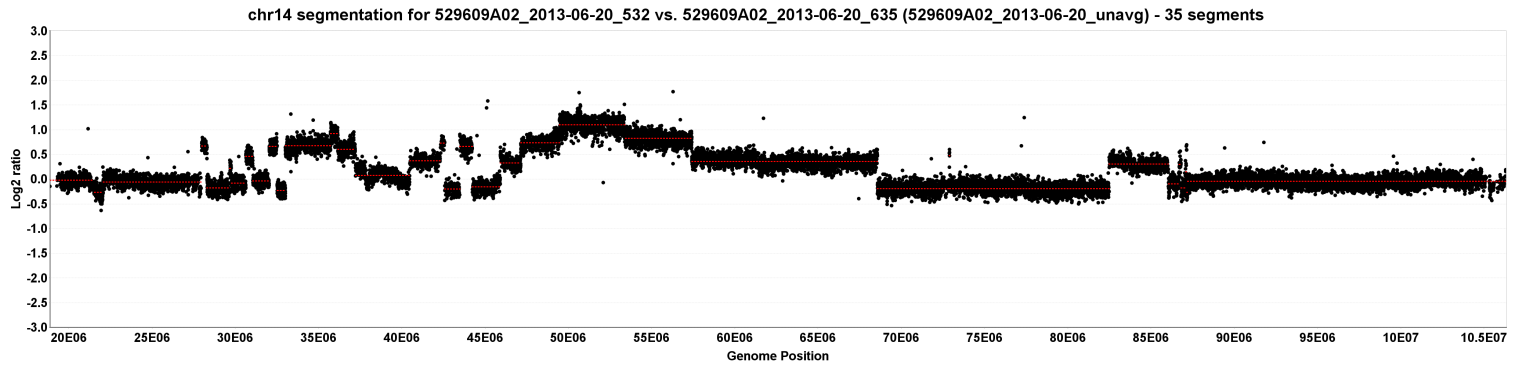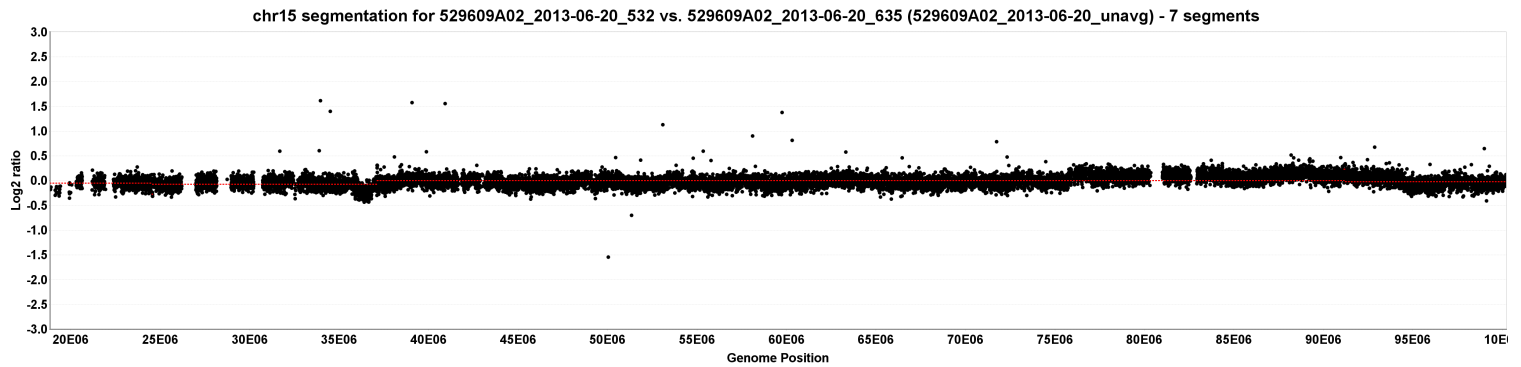

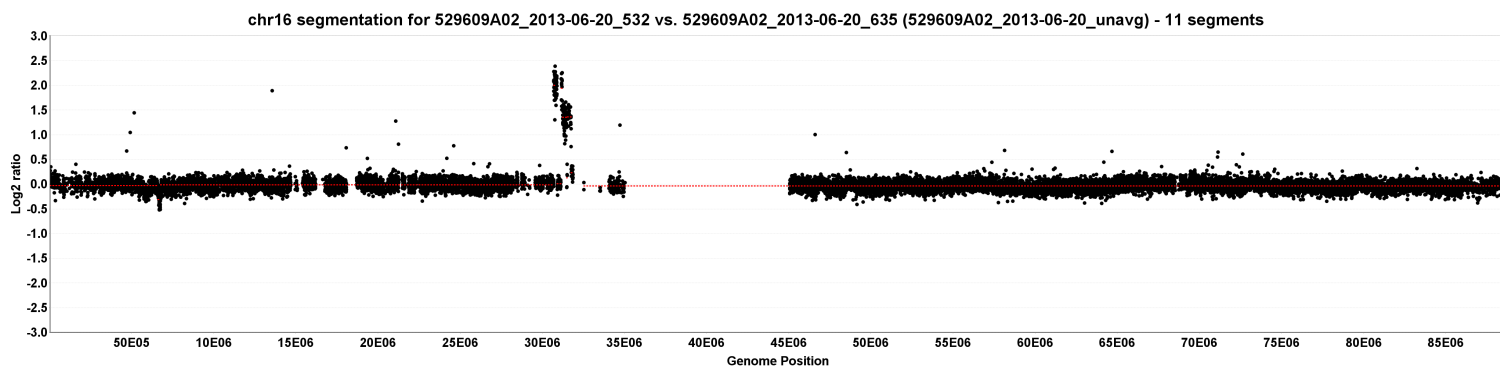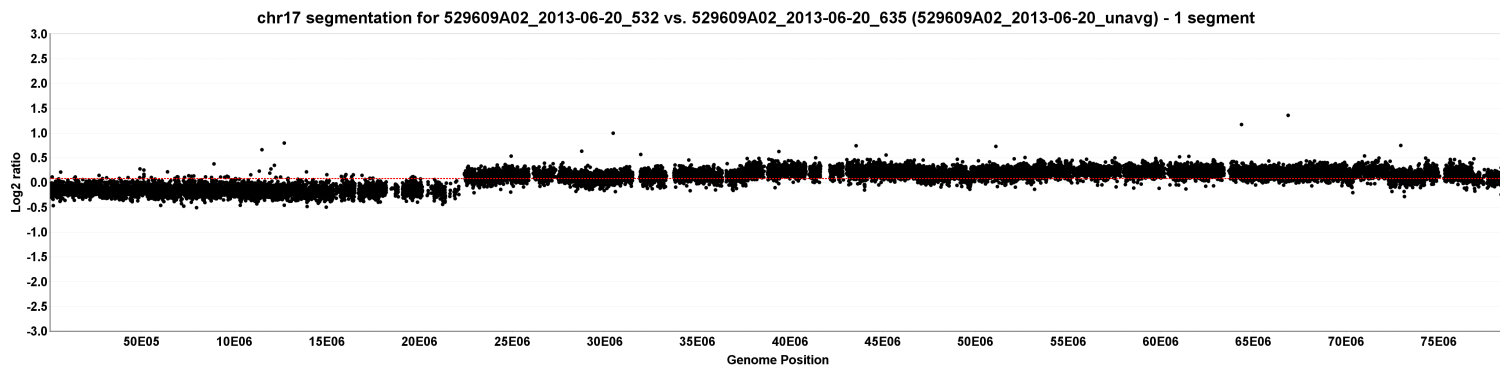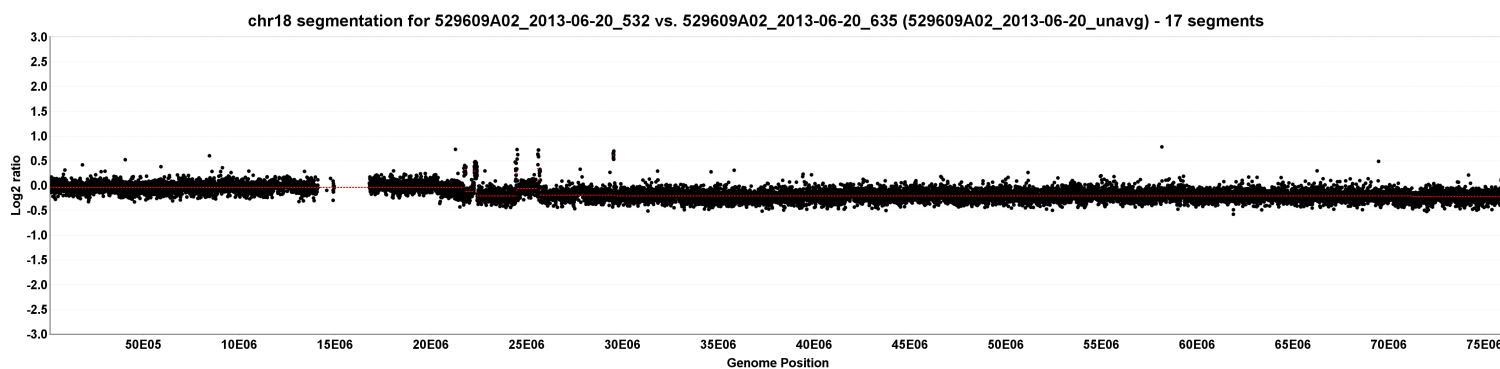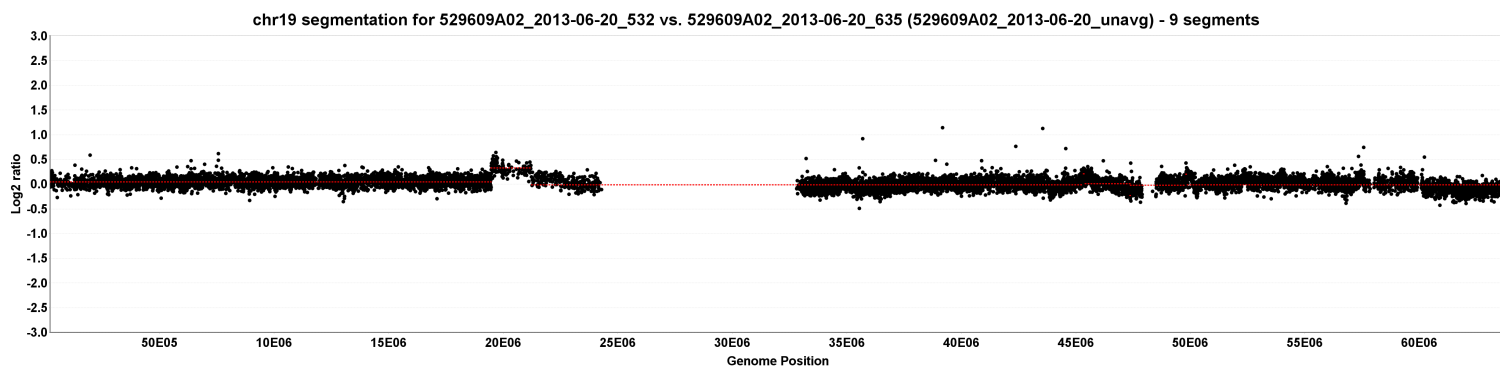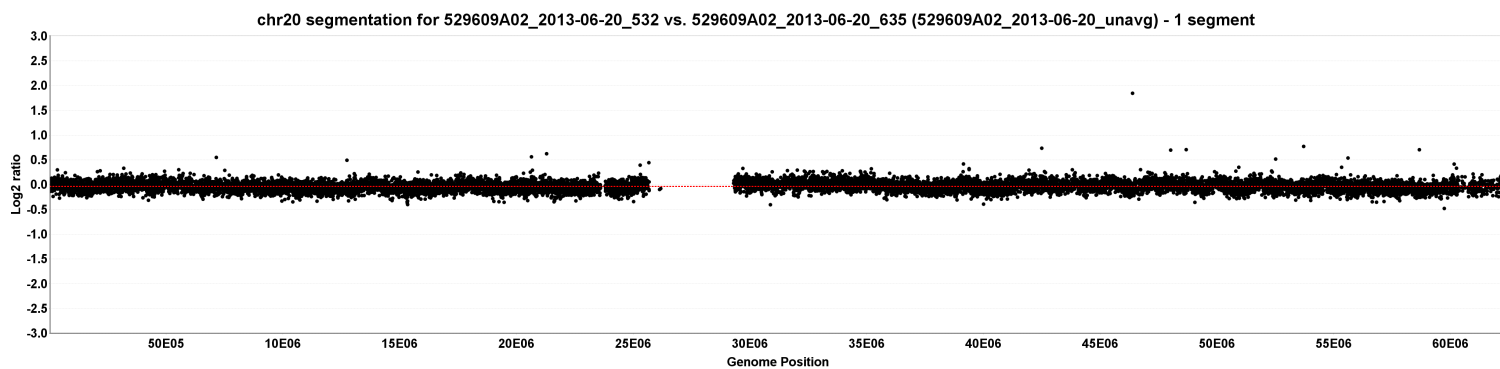

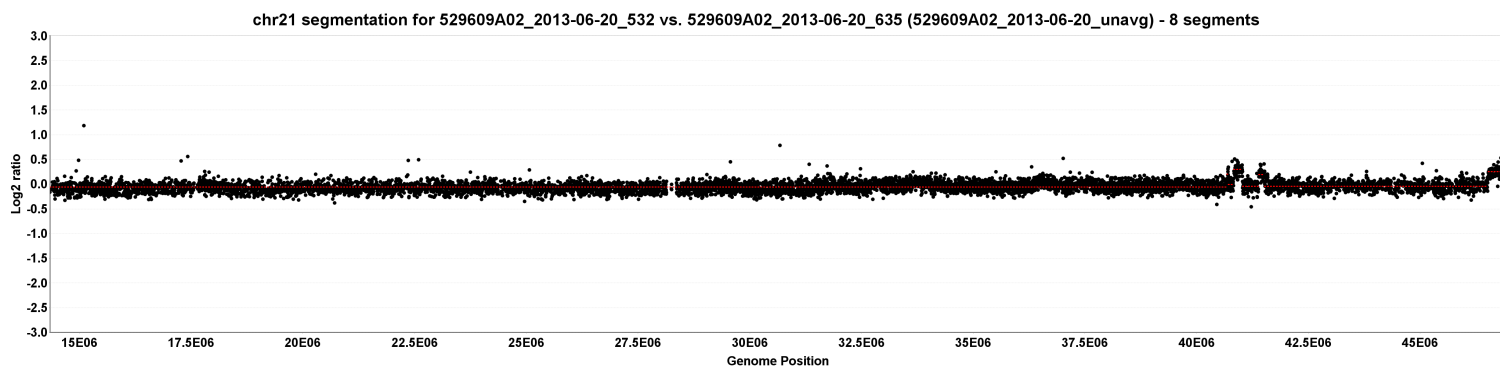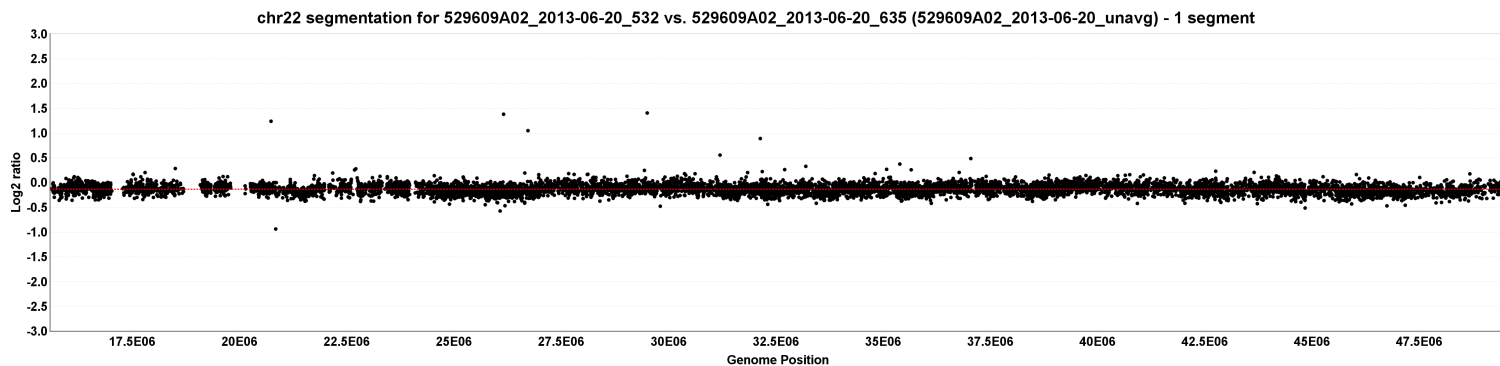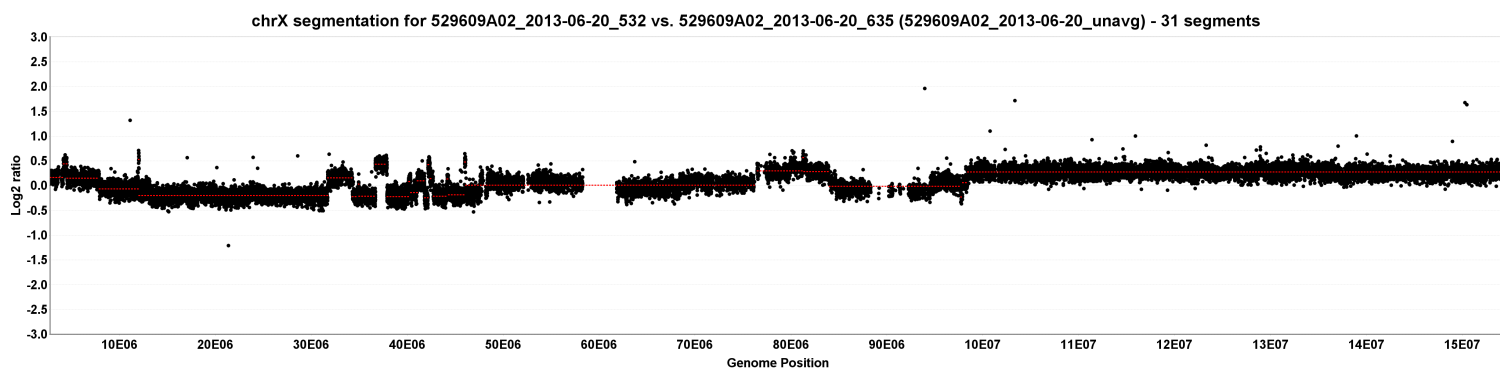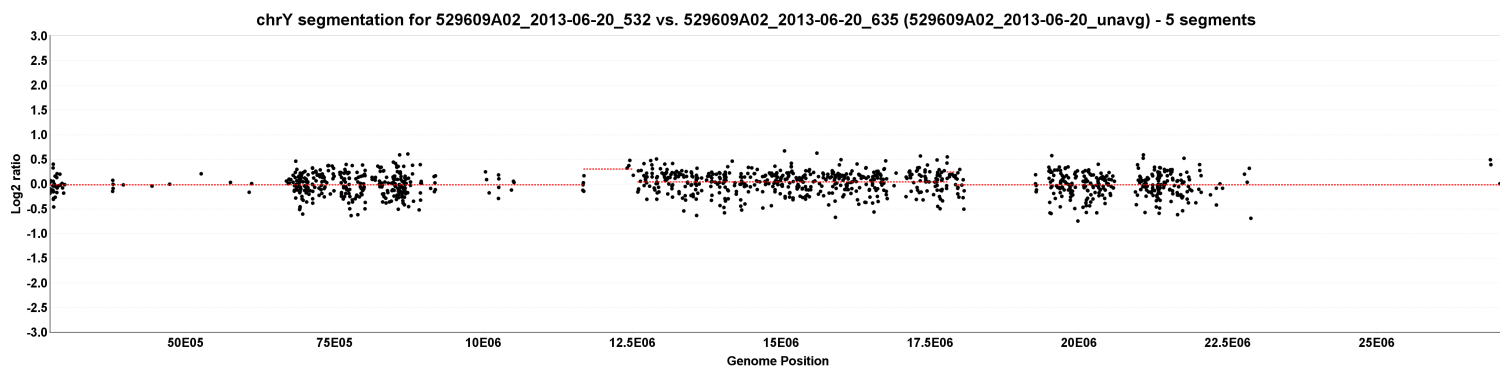

Supplement: S7 File — (PDF) [file pone.0169098.s007.pdf]

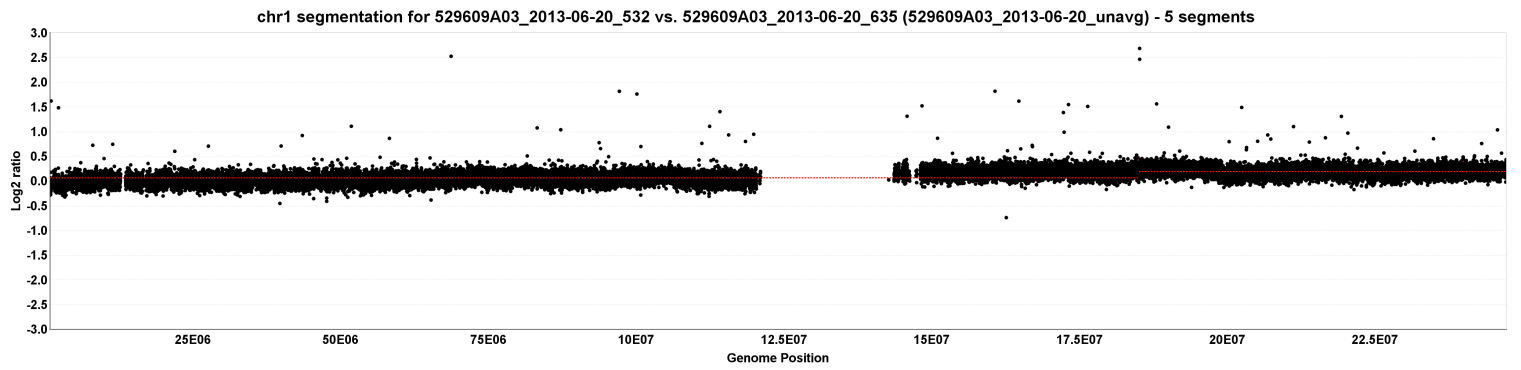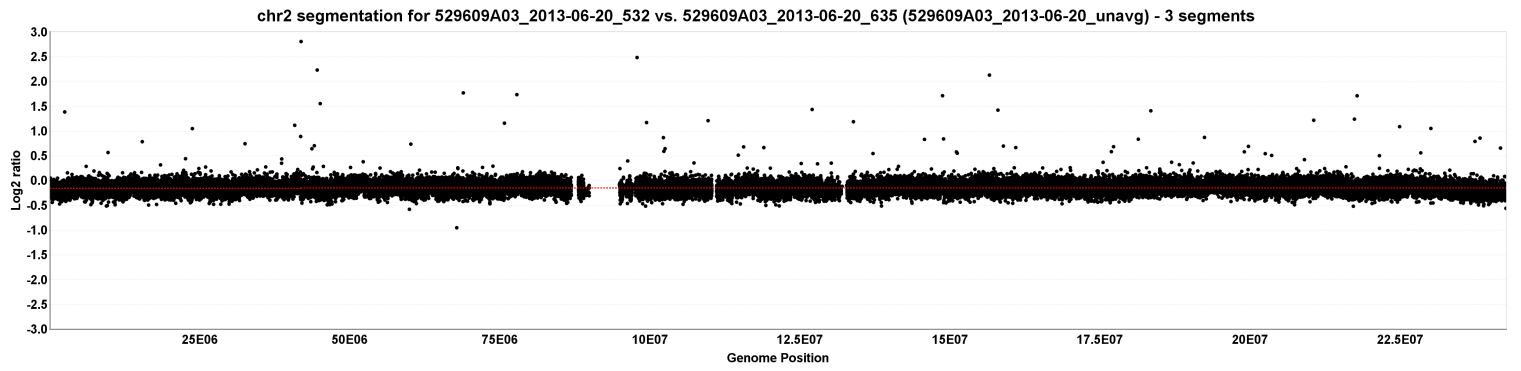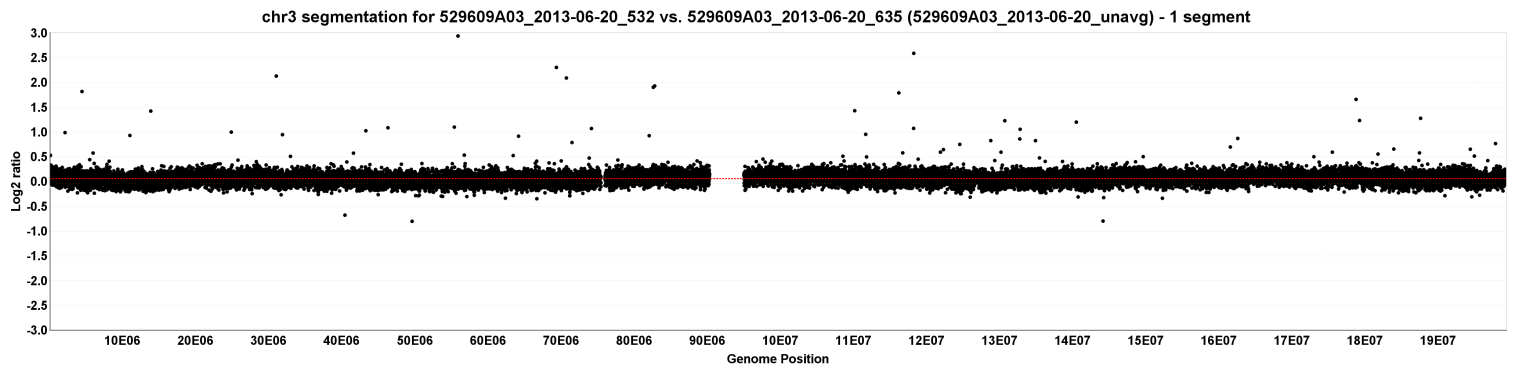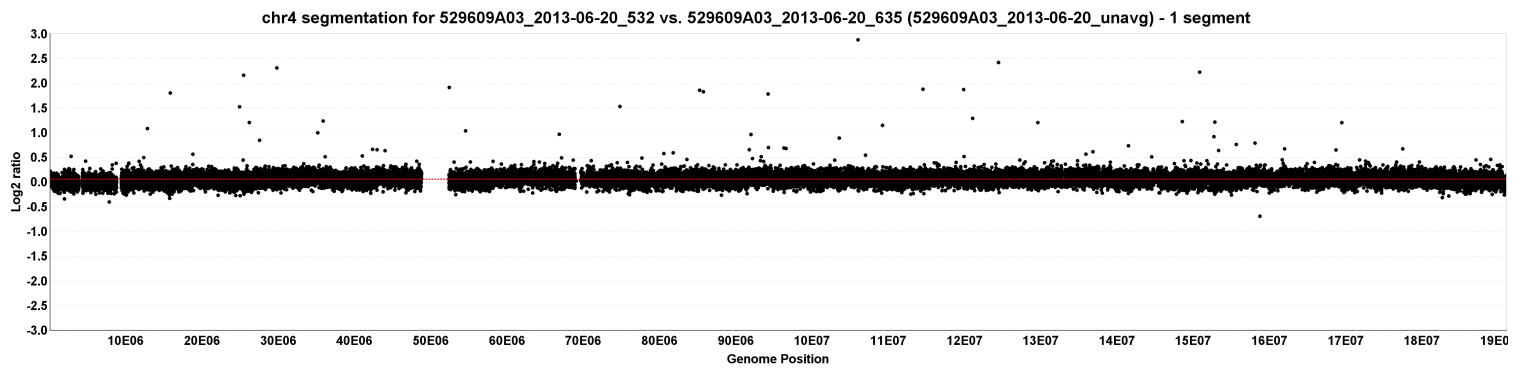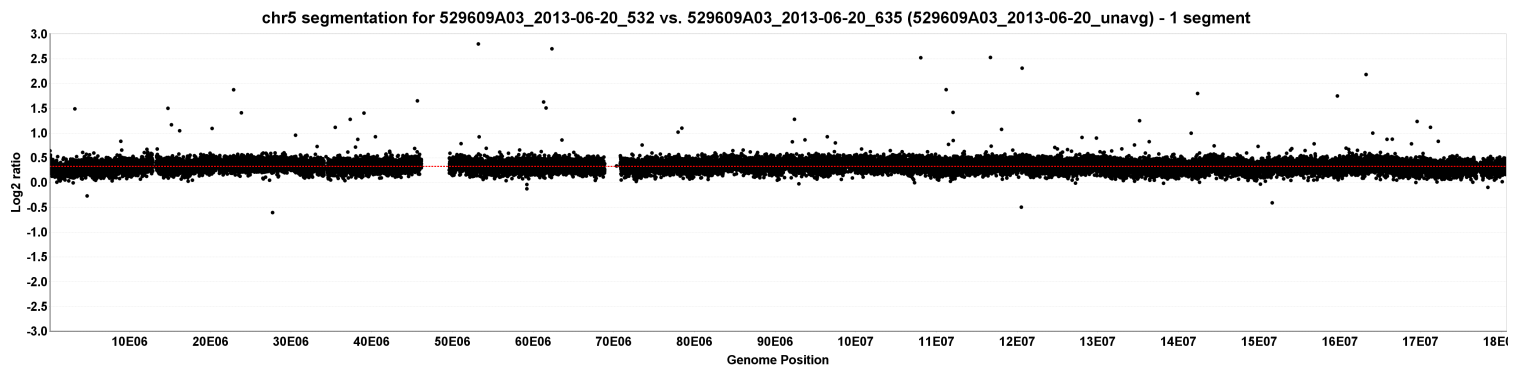

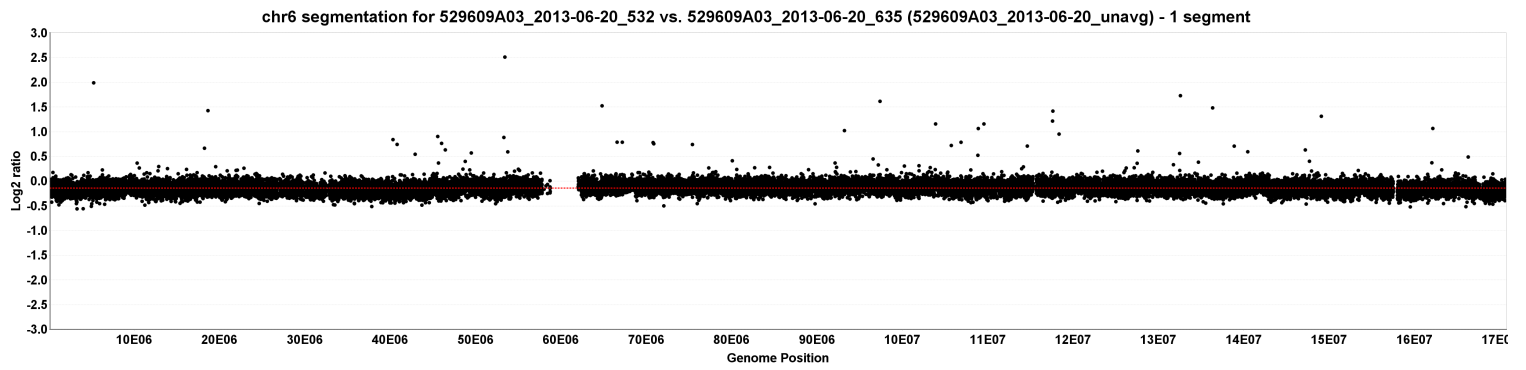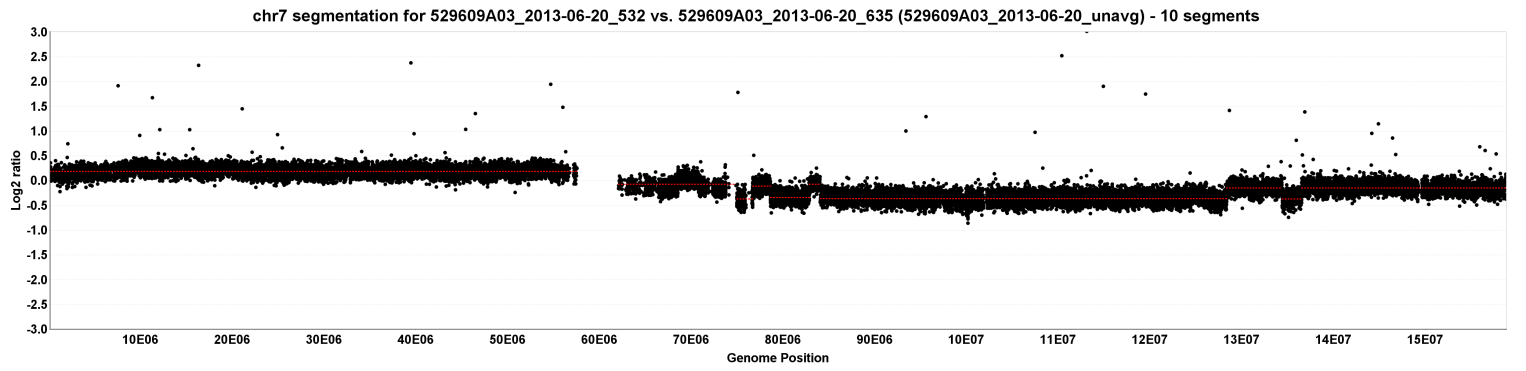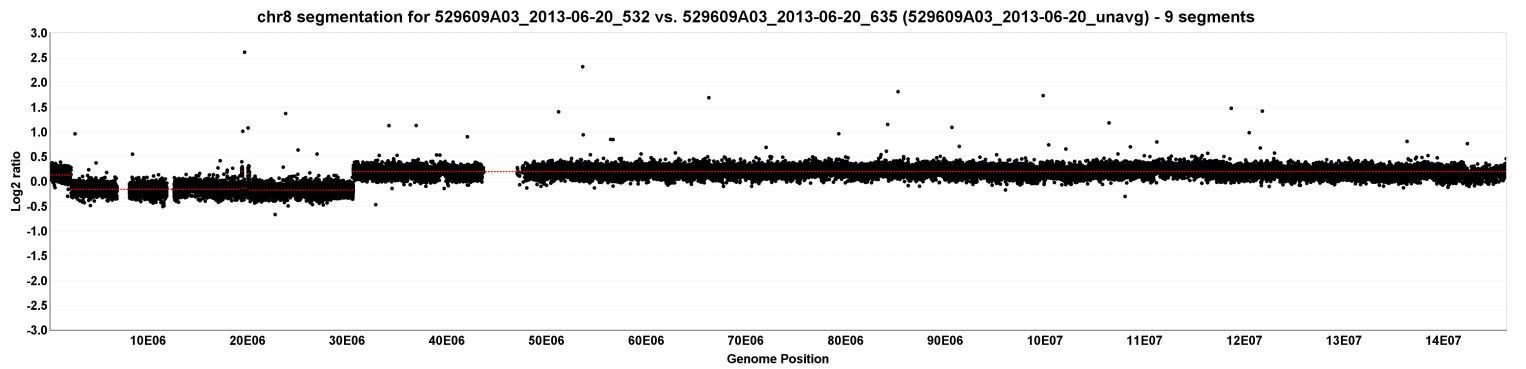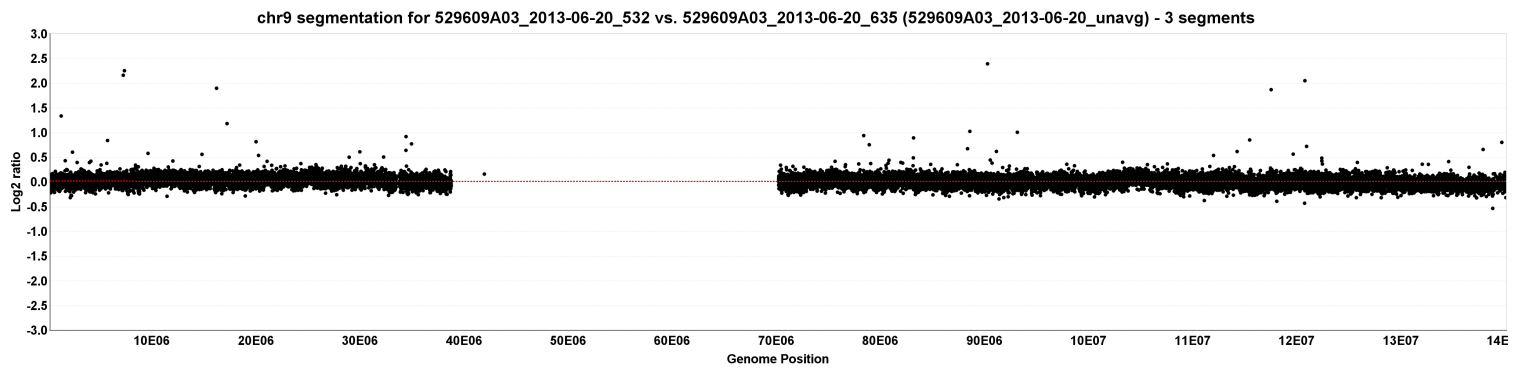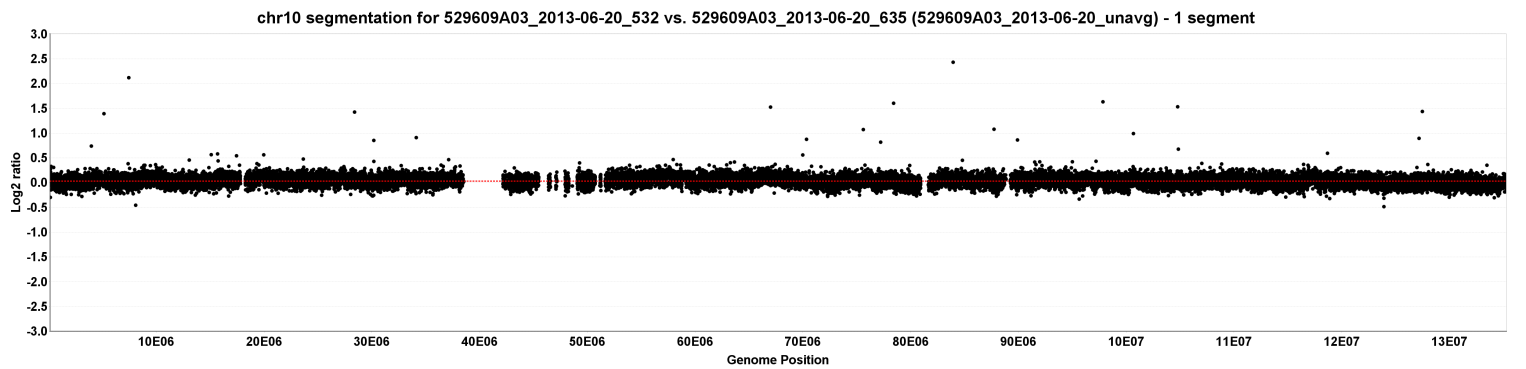

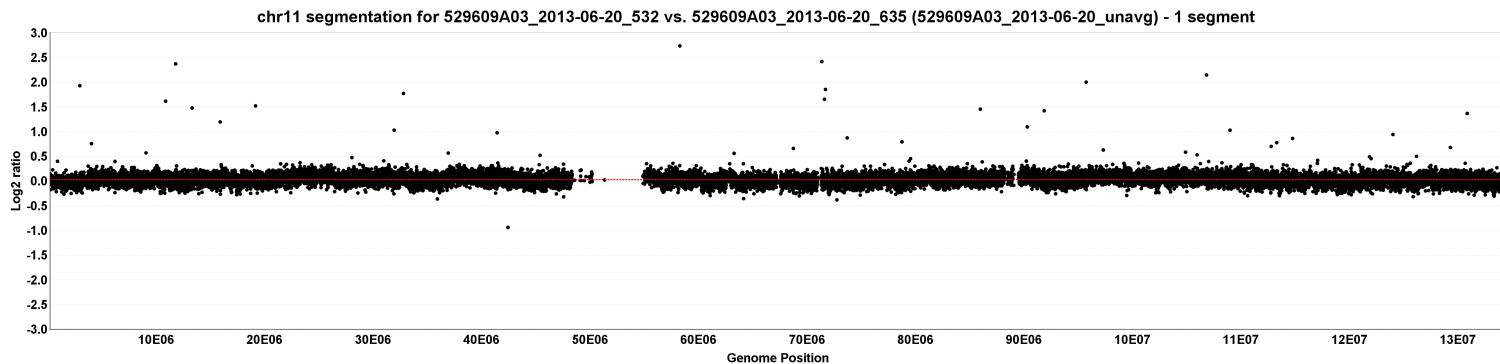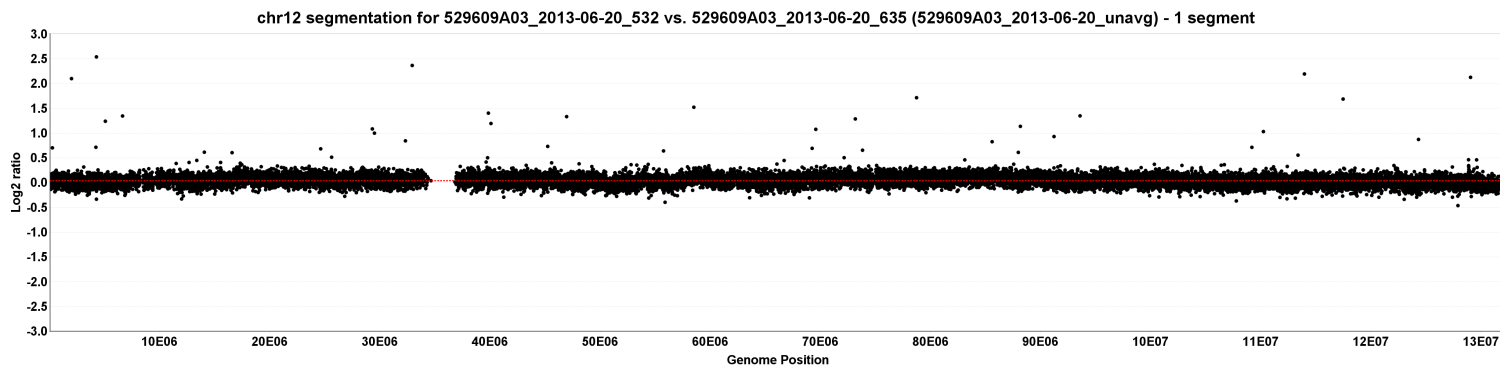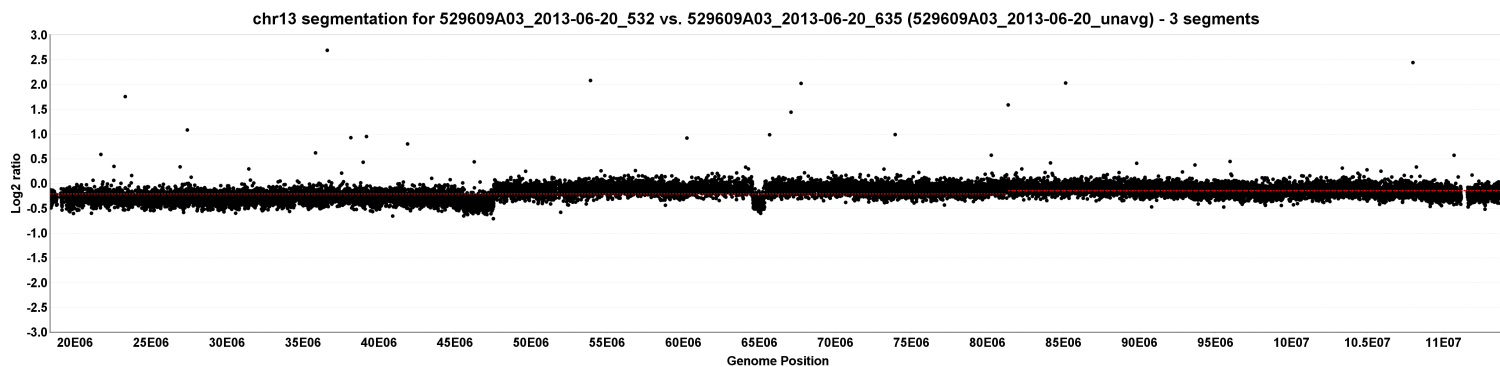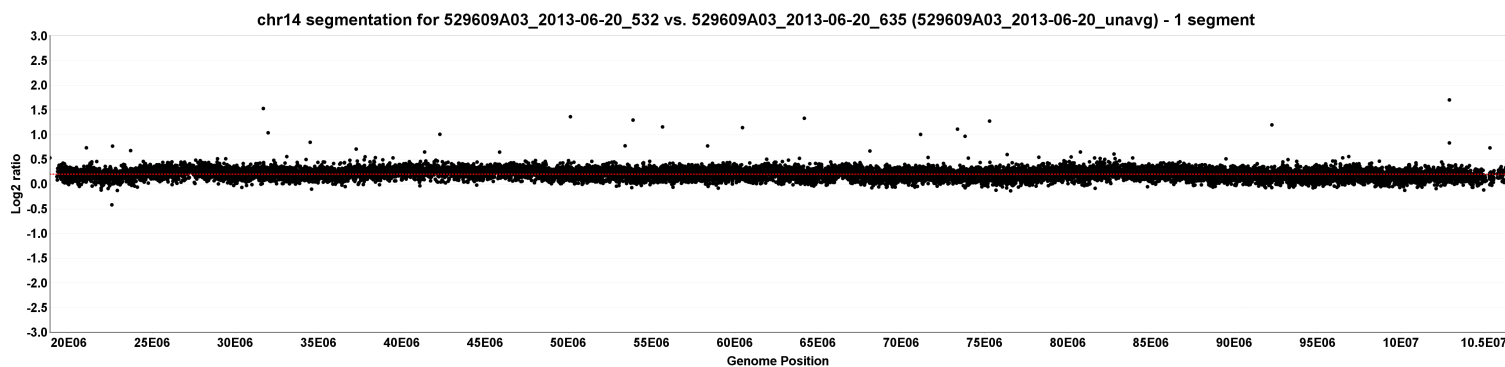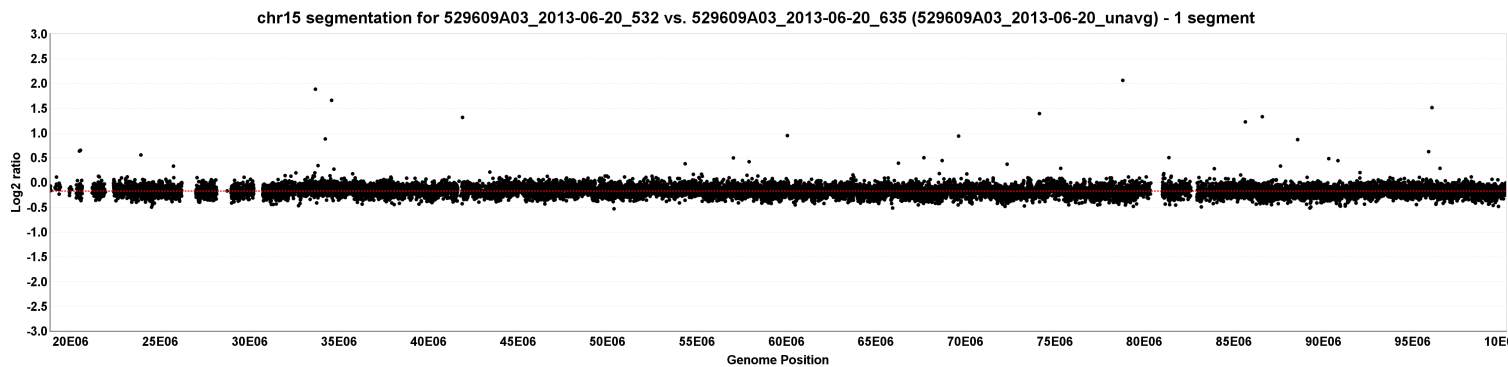

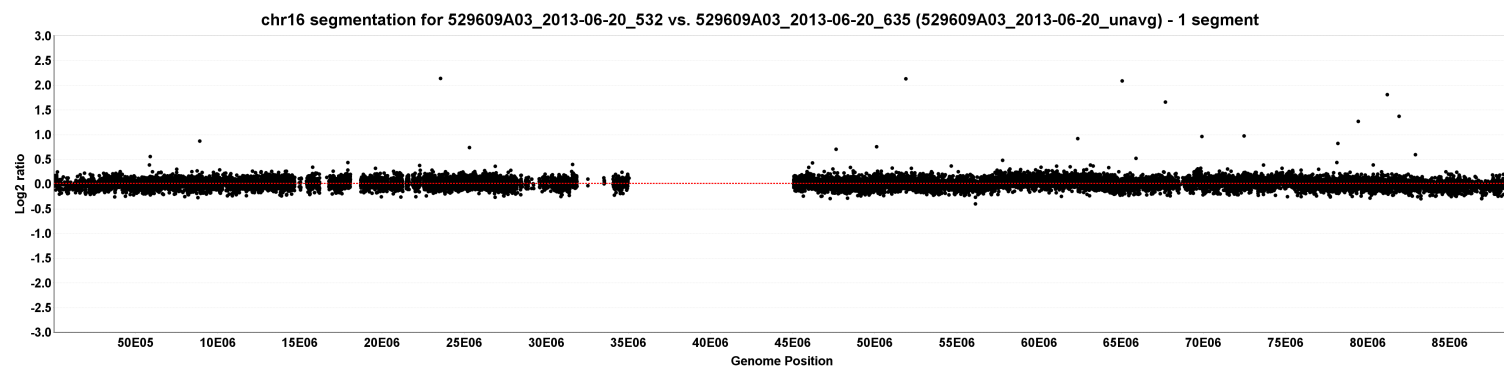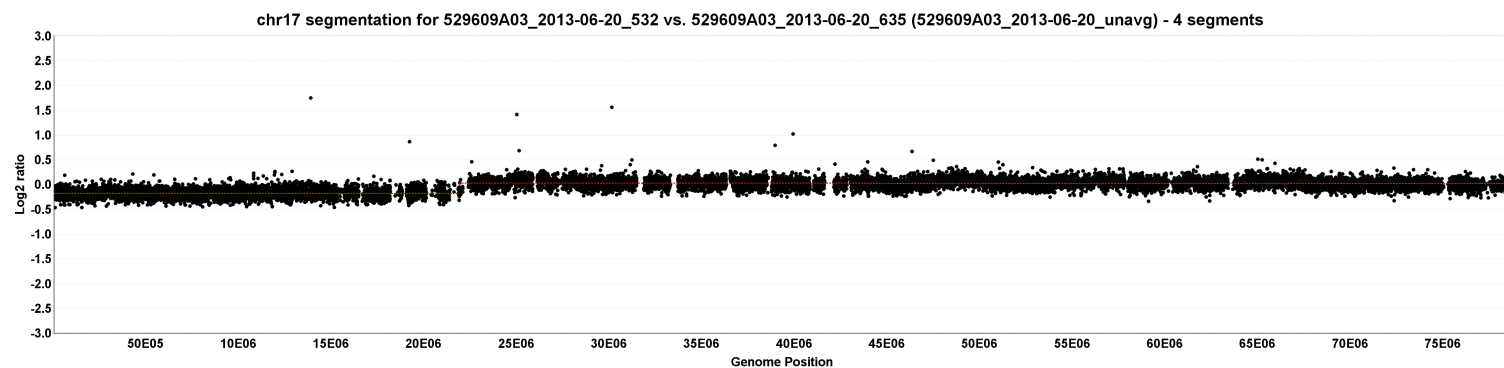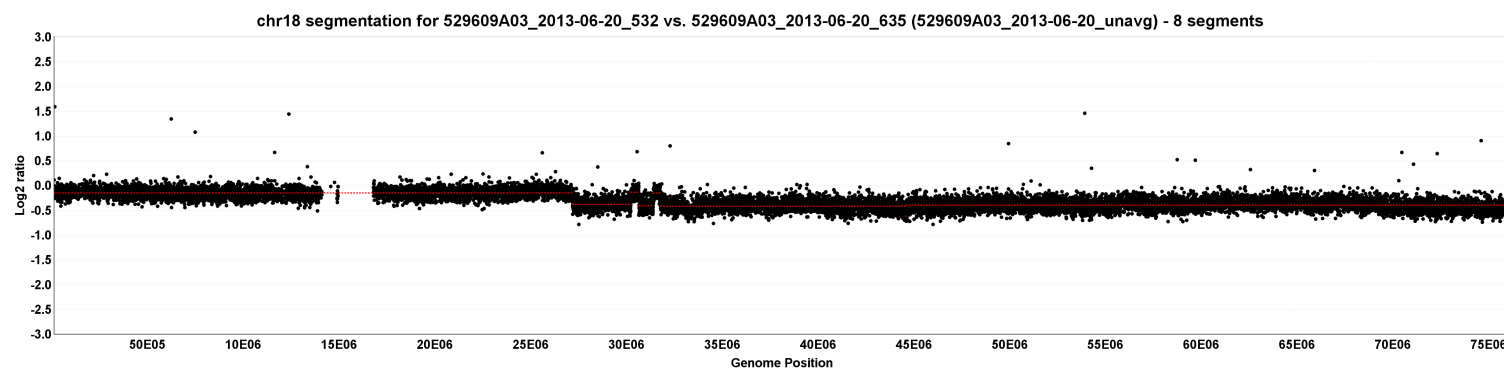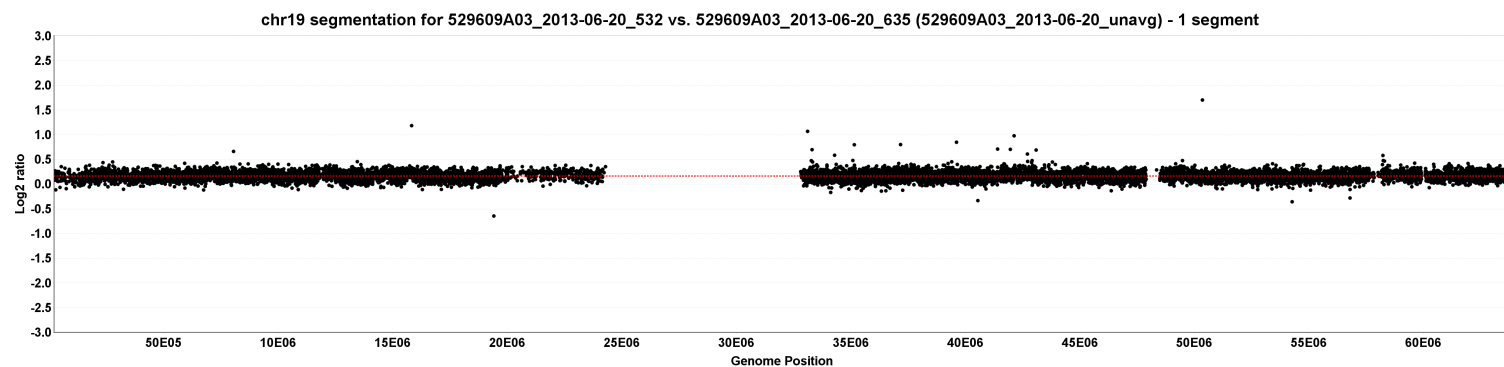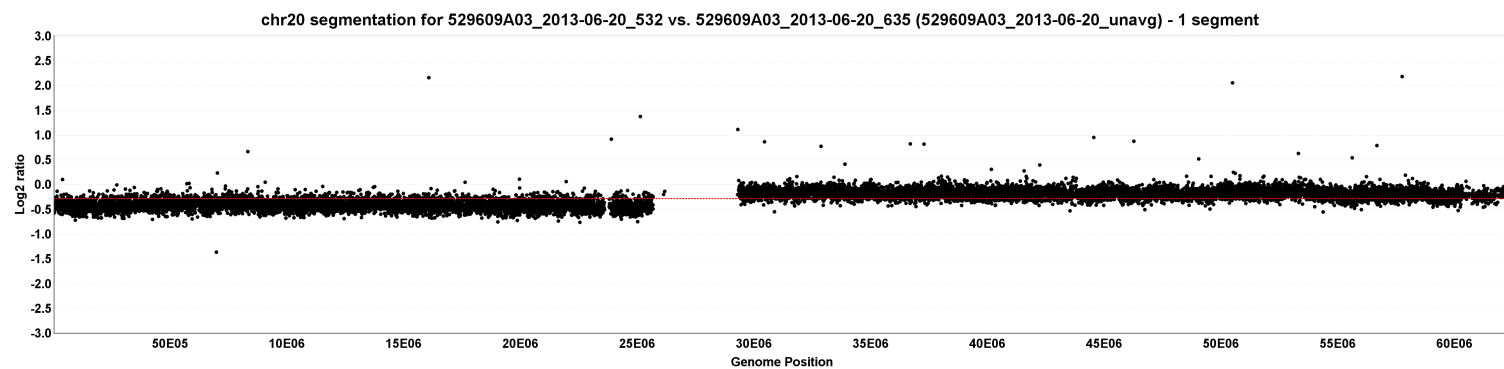

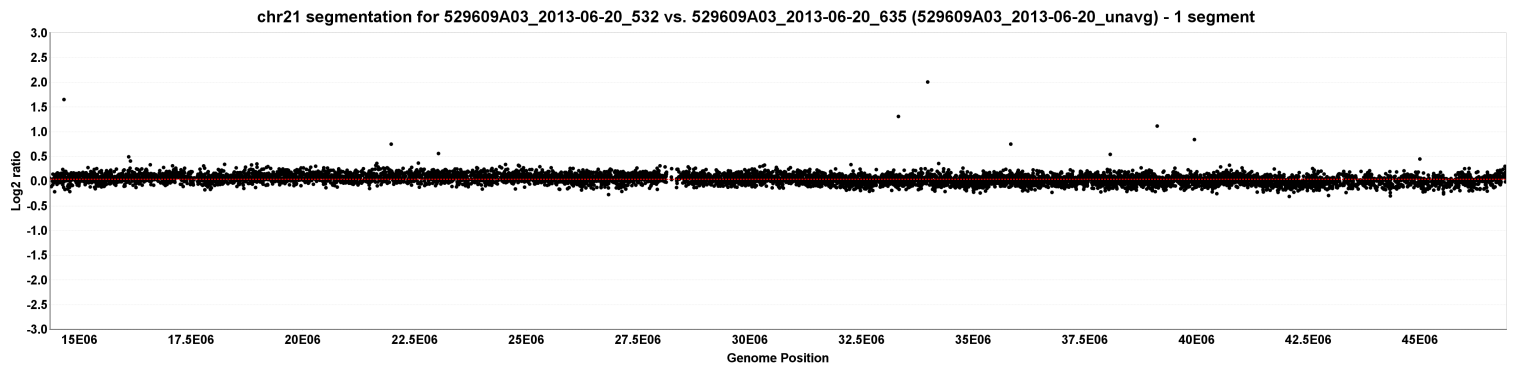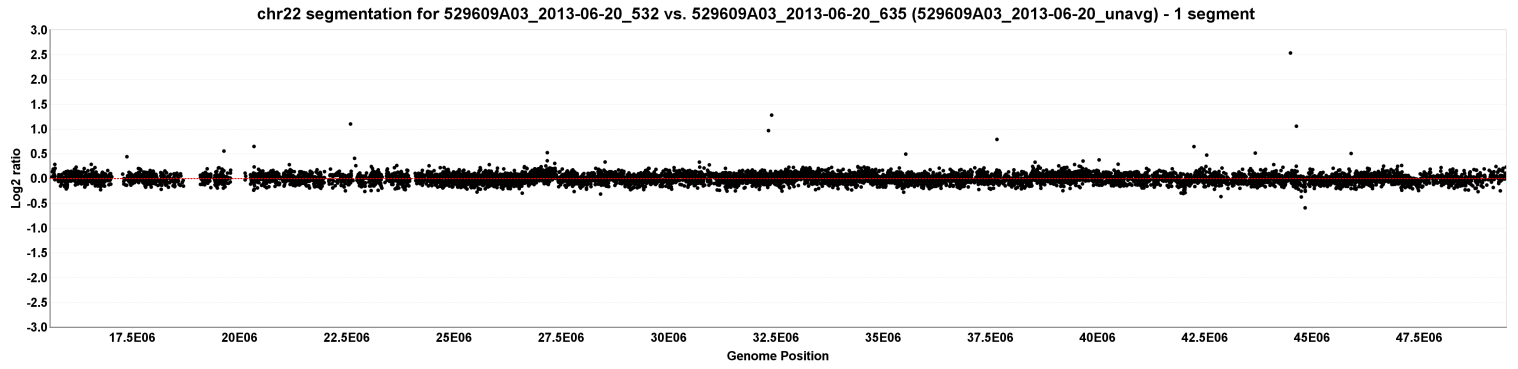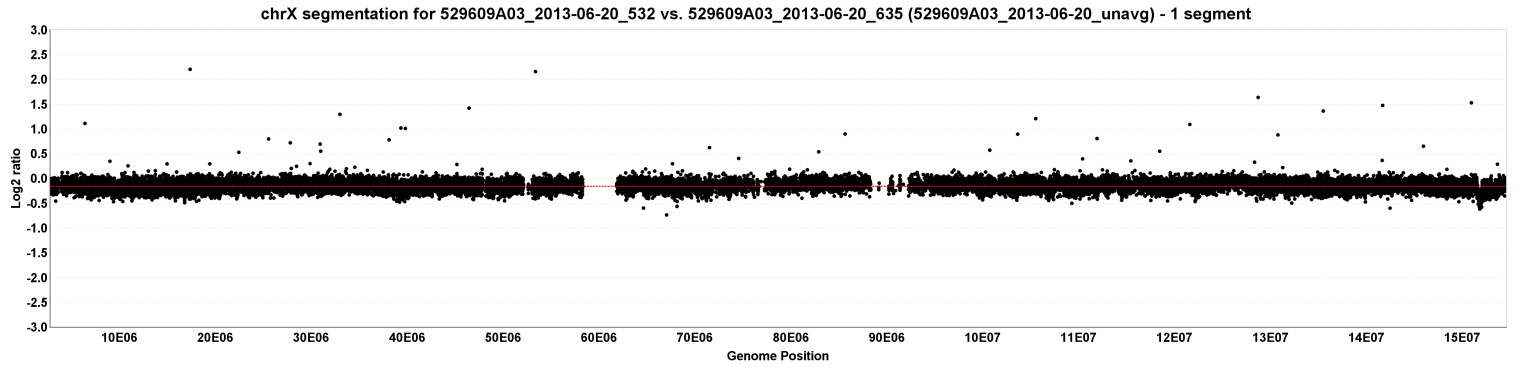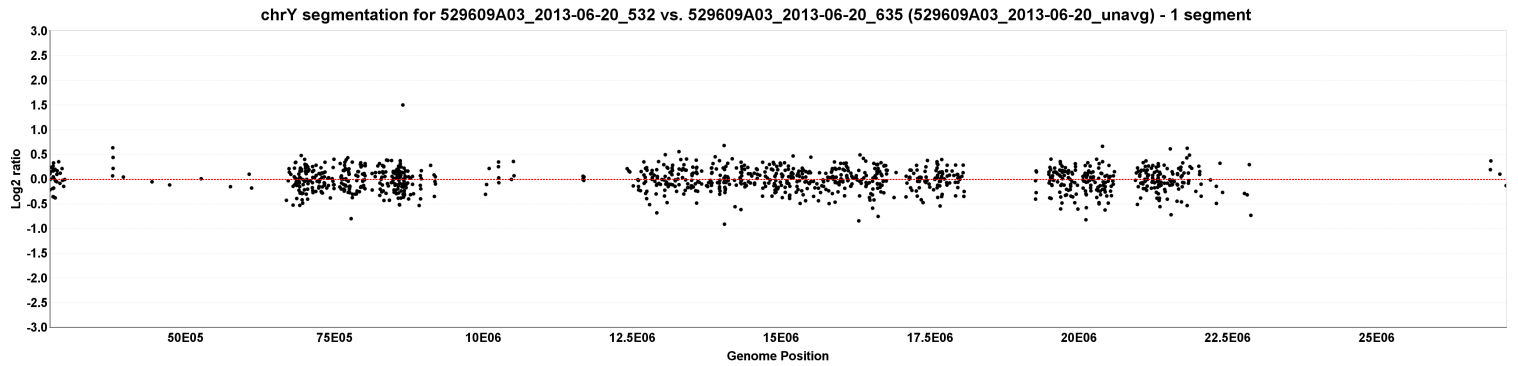

Supplement: S8 File — (PDF) [file pone.0169098.s008.pdf]

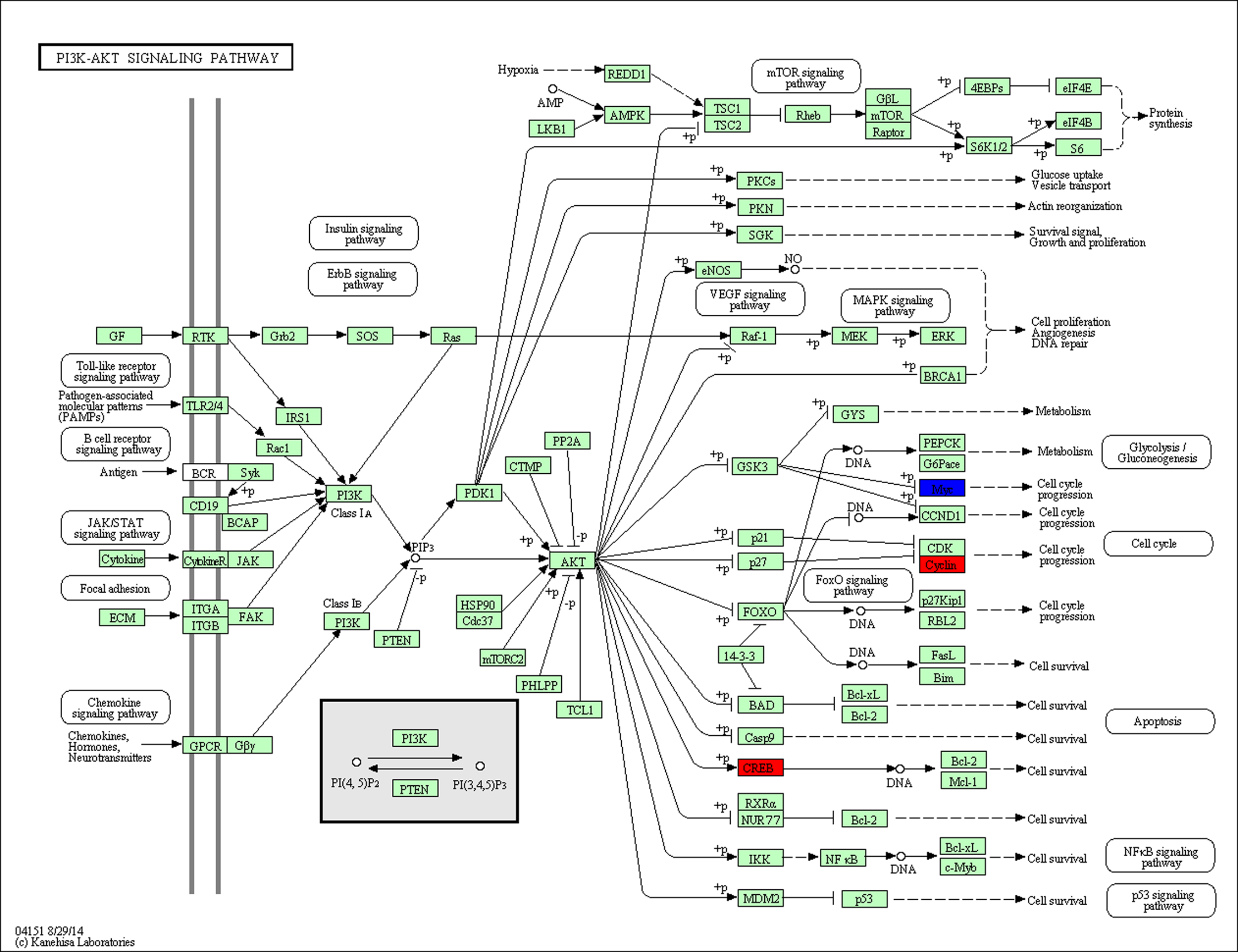

Supplement: S1 Fig — (TIF) [file pone.0169098.s009.tif]

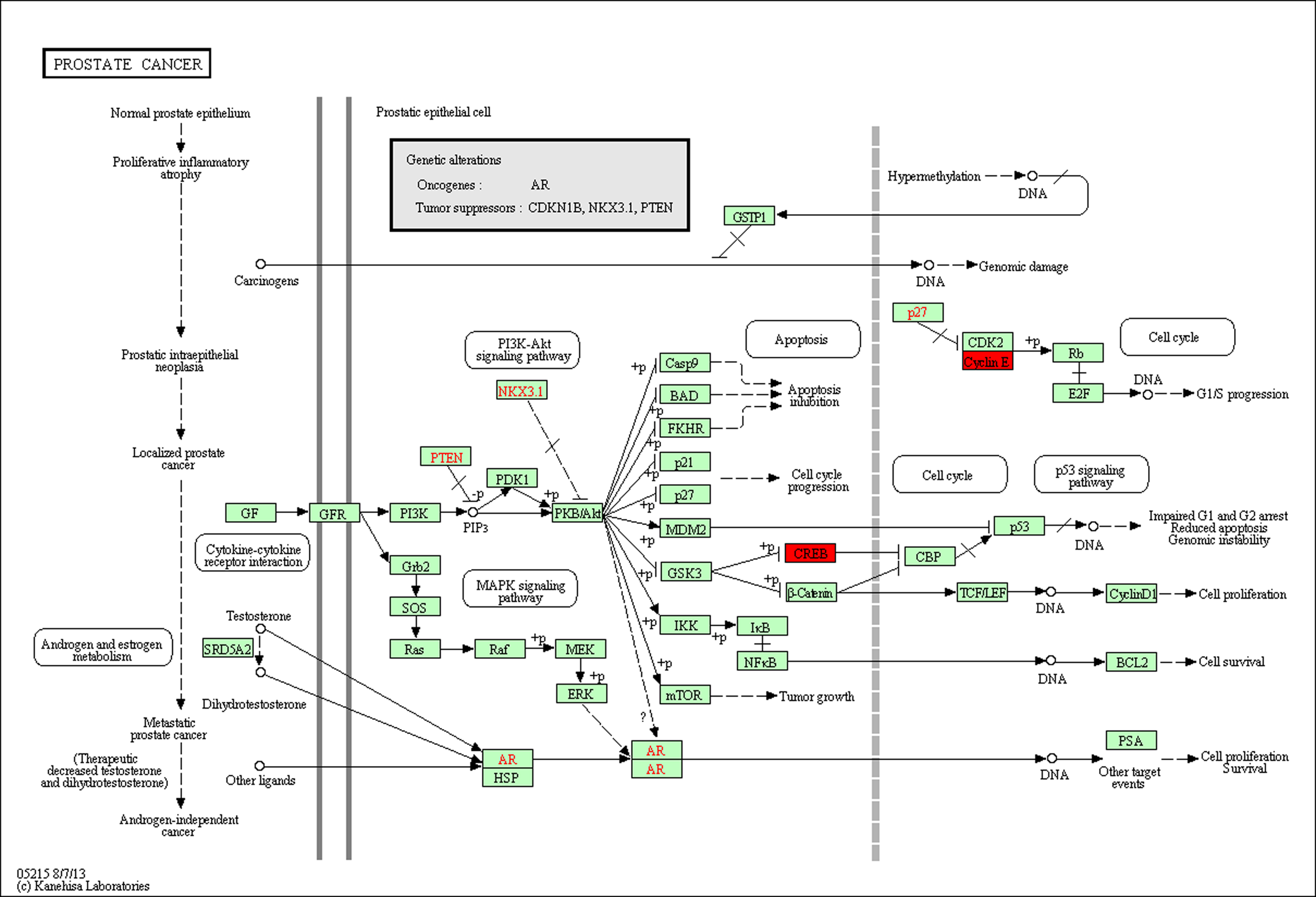

Supplement: S2 Fig — (TIF) [file pone.0169098.s010.tif]

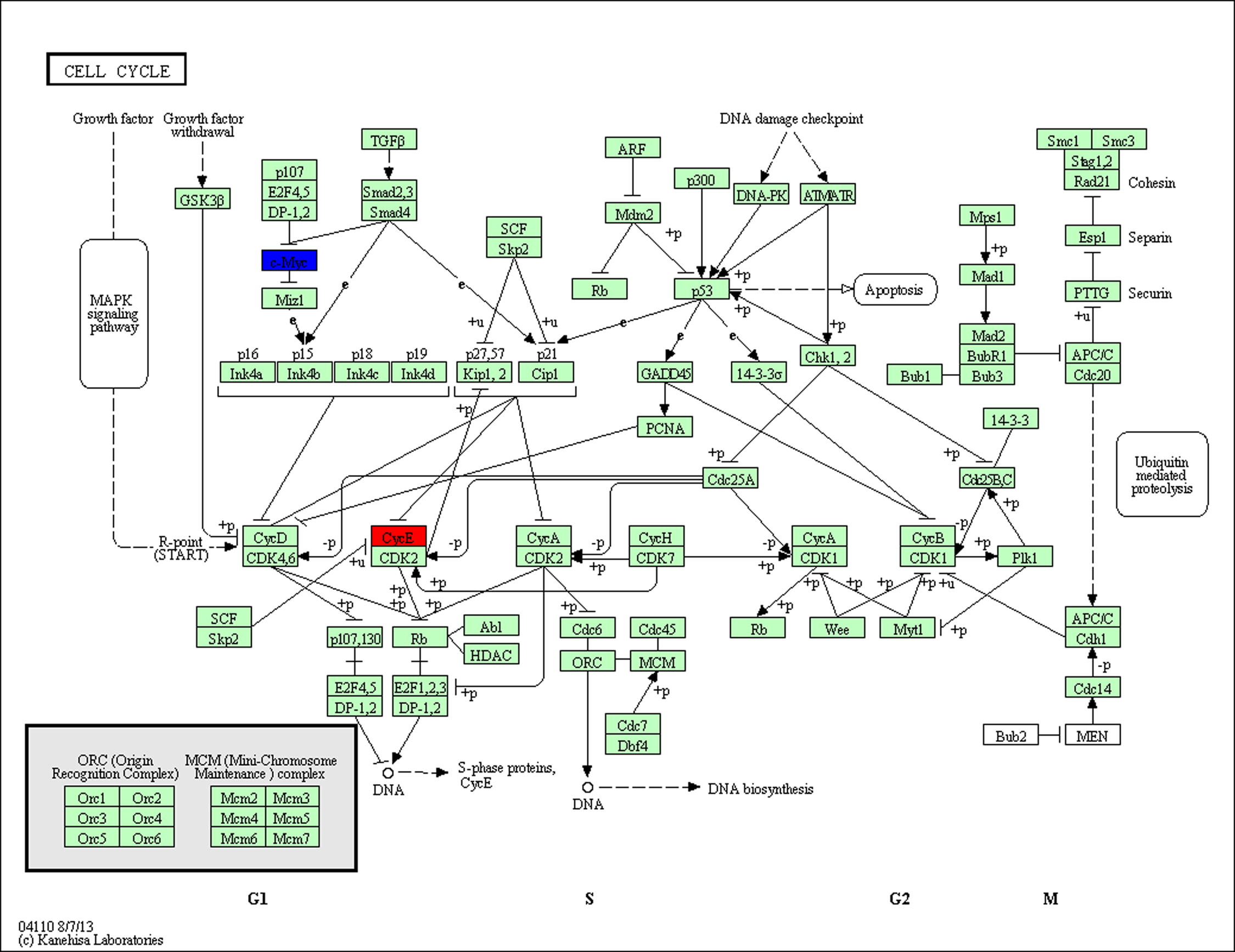

Supplement: S3 Fig — (TIF) [file pone.0169098.s011.tif]

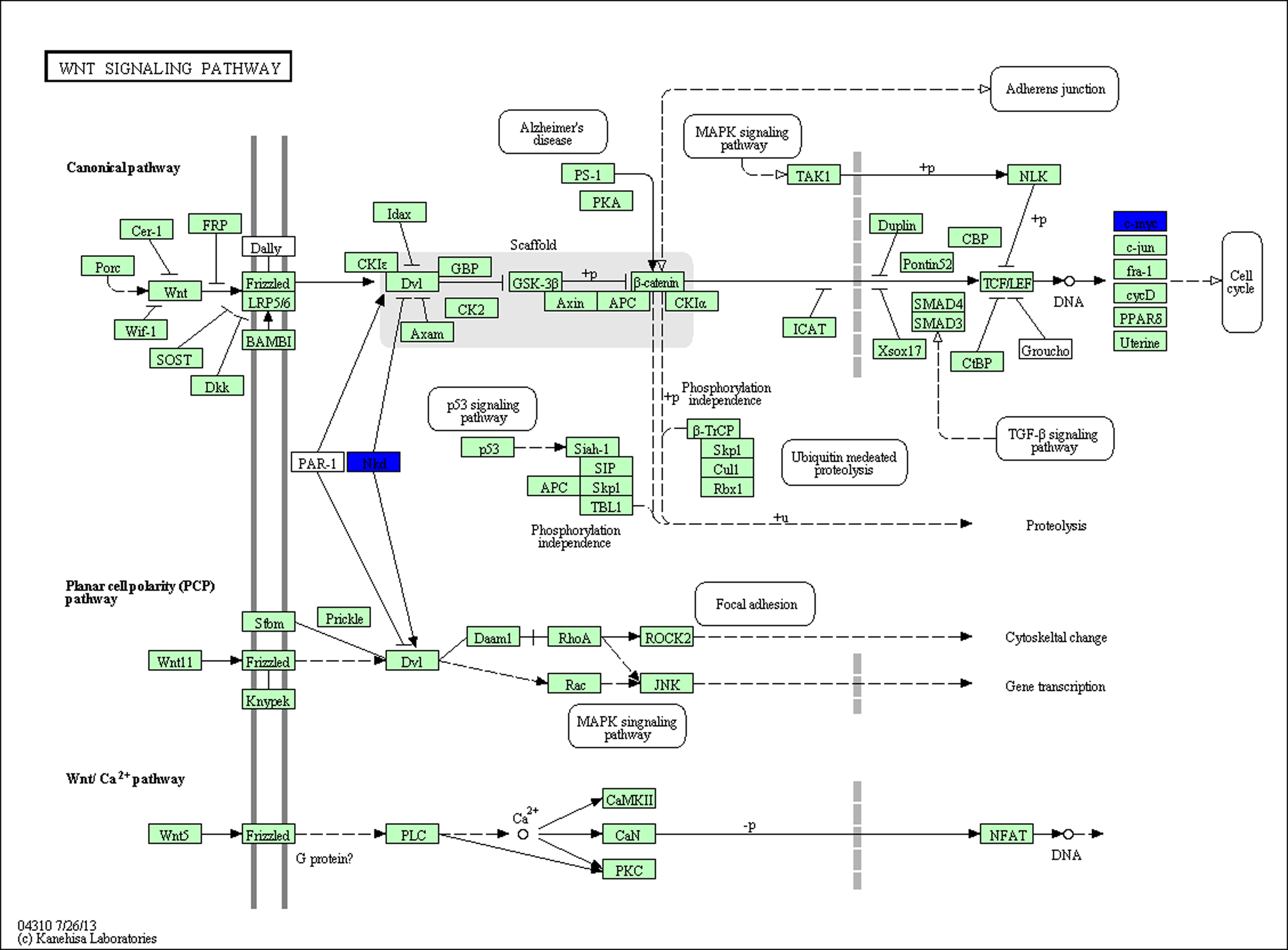

Supplement: S4 Fig — (TIF) [file pone.0169098.s012.tif]

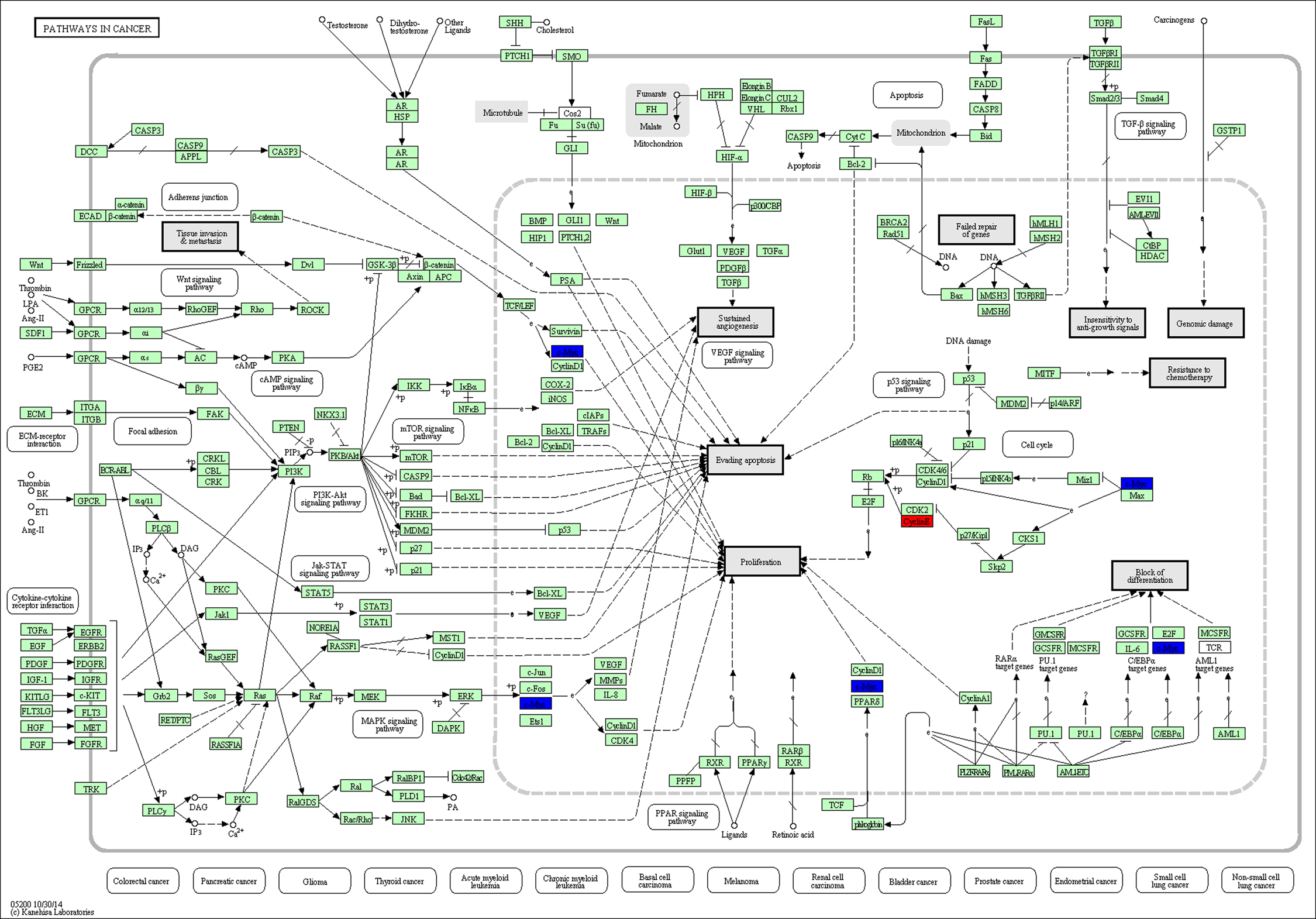

Supplement: S5 Fig — (TIF) [file pone.0169098.s013.tif]
